# Identification of a STAT5 Target Gene, *Dpf3*, Provides Novel Insights in Chronic Lymphocytic Leukemia

**DOI:** 10.1371/journal.pone.0076155

**Published:** 2013-10-14

**Authors:** Marina Theodorou, Matthaios Speletas, Antigoni Mamara, Georgia Papachristopoulou, Vassiliki Lazou, Andreas Scorilas, Eleni Katsantoni

**Affiliations:** 1 Hematology/Oncology Division, Biomedical Research Foundation, Academy of Athens, Athens, Greece; 2 Department of Immunology and Histocompatibility, Medical School, University of Thessaly, Larissa, Greece; 3 Department of Biochemistry and Molecular Biology, Faculty of Biology, University of Athens, Athens, Greece; Virginia Commonwealth University, United States of America

## Abstract

STAT5 controls essential cellular functions and is encoded by two genes, *Stat5a* and *Stat5b*. To provide insight to the mechanisms linking hematologic malignancy to STAT5 activation/regulation of target genes, we identified STAT5 target genes and focused on *Dpf3* gene, which encodes for an epigenetic factor. *Dpf3* expression was induced upon IL-3 stimulation in Ba/F3 cells, while strong binding of both STAT5a and STAT5b was detected in its promoter. Reduced expression of *Dpf3* was detected in Ba/F3 cells with *Stat5a* and *Stat5b* knock-down, suggesting that this gene is positively regulated by STAT5, upon IL-3 stimulation. Furthermore, this gene was significantly up-regulated in CLL patients, where *DPF3* gene/protein up-regulation and strong STAT5 binding to the *DPF3* promoter, correlated with increased STAT5 activation, mainly in non-malignant myeloid cells (granulocytes). Our findings provide insights in the STAT5 dependent transcriptional regulation of *Dpf3*, and demonstrate for the first time increased STAT5 activation in granulocytes of CLL patients. Novel routes of investigation are opened to facilitate the understanding of the role of STAT5 activation in the communication between non-malignant myeloid and malignant B-cells, and the functions of STAT5 target genes networks in CLL biology.

## Introduction

Signal transducers and activators of transcription (STATs) constitute a family of transcription factors that regulate important cellular processes [1]. The STAT family is comprised of seven members (STAT1, STAT2, STAT3, STAT4, STAT5a, STAT5b, STAT6). Upon cytokine, growth factor or hormone stimulation STATs are phosphorylated, dimerize and translocate from the cytoplasm into the nucleus, where they bind to specific regulatory elements to tightly regulate gene transcription. In addition to their essential normal functions, STATs have been implicated in the pathogenesis of various malignancies.

There are two STAT5 proteins, STAT5a and STAT5b, which are encoded by two distinct but chromosomally linked genes and share at least 90% homology in their amino acid sequences [2]. STAT5a and STAT5b constitute important components of key signal-transduction pathways and are activated by various cytokines and growth factors [1], [3]. Constitutive activation of STAT5 is a hallmark for hematopoietic [4] and solid malignancies [5], [6]. STAT5 is also implicated in the self-renewal of hematopoietic stem cells [7] and regulates Fas-mediated cell death [8]. Mice with a deletion of the *Stat5a* and *Stat5b* genes helped to reveal the physiological functions of both proteins. In particular, *Stat5a*-/- mice exhibit defects in mammary gland development [9] and prolactin response, whereas *Stat5b*-/- mice are defective in growth hormone response [3], [10]. In addition, mice lacking *Stat5a* or *Stat5b* show phenotypic differences in hematopoiesis [11], [12]. Overall, the knock-out studies indicate that the functional differences between STAT5a and STAT5b may result from the ability of each protein to regulate specific target gene networks.

Several studies have identified STAT5 target genes [13]–[17] and genome-wide mapping has been performed [18]–[23], however the complete map of target genes in pro-B cells has not been fully characterized yet. Therefore, many questions remain unanswered concerning how STAT5 positively or negatively influences target gene transcription. Identification of novel STAT5 target genes is crucial for understanding the role of STAT5 not only in physiological cellular processes, but also in oncogenesis. Furthermore, the mechanisms linking oncogenesis to the interplay of STAT5 activation/regulation of target genes in chronic lymphocytic leukemia (CLL) are unexplored. To this end we focused on identification of STAT5 target genes. By utilizing a newly identified target, *Dpf3,* we aimed at providing insights on the role of *DPF3*/STAT5 in CLL. Our findings highlight the STAT5 dependent transcriptional regulation of *Dpf3*, demonstrating for the first time increased STAT5 activation in non-malignant myeloid cells of CLL patients and open novel routes of investigation to facilitate the understanding of the role of activated STAT5 in non-malignant myeloid and malignant B-cells communication.

## Methods

### Constructs

The mouse *Stat5a* cDNA and the bio-TEV sequences were amplified by PCR from the pMX-puro-STAT5a and pTRE-bio-TEV vectors, respectively [with the bio-tag fused to the TEV cleavage site in the NotI-NcoI restriction sites of the pTRE vector (Clontech, Mountain View, CA, USA)]. The EF1a-bioSTAT5a construct was generated by cloning the NotI-EcoRI bio-TEV fragment, and the EcoRI-KpnI *Stat5a* cDNA fragment into the NotI-KpnI sites of the pBud-neo vector [pBudCE4.1 vector (Invitrogen, Paisley, UK) with the neo cassette cloned in the NheI site]. New restriction sites required for cloning were inserted by PCR.

### Cell lines, transfections and PB cells isolation

Ba/F3 cells [24] were maintained in RPMI 1640/10% FBS (fetal bovine serum), 1x P/S (100 U/ml penicillin and 100 µg/ml streptomycin) and 1 ng/ml recombinant murine IL-3 (PeproTech, London, UK). Ba/F3 cells were electroporated with the EF1a-BirA plasmid and stable clones were selected with puromycin (2 µg/ml). A stable BirA/Ba/F3 clone was then electroporated with EF1a-bioSTAT5a. Stable double clones were selected with puromycin (2 µg/ml) and geneticin G418 (1000 µg/ml). Stimulation of Ba/F3 cells was performed with 10 ng/ml ΙL-3 for 30 min (minutes), following a period of deprivation of IL-3 for 6 h (hours). JVM-2 [25] and EHEB [26] cells were grown in RPMI 1640/15% FBS/antibiotics.

PBMCs (peripheral blood mononuclear cells) were isolated from PB (peripheral blood) with Histopaque-1077 (Sigma, St. Louis, MO, USA) and leukocyte fractions (mononuclear cells and granulocytes) with Histopaque-1119 and Histopaque-1077 (Sigma).

### ChIP and chromatin streptavidin precipitation

Ba/F3, JVM-2 and EHEB cells were cross-linked with 1% formaldehyde added to the culture medium for 15 min at room temperature (RmT). PBMCs and granulocytes were cross-linked directly after isolation in 10 ml RPMI 1640/10% FBS with 1% formaldehyde for 15 min at RmT. ChIPs (chromatin immunoprecipitations) were carried out according to the Upstate protocol, with anti-STAT5a antibody (sc-1081X), anti-STAT5b (sc-1656X), Rabbit IgG (sc-2027) or Mouse IgG (sc-2025) (Santa Cruz Biotechnology, Inc., Santa Cruz, CA, USA).

Chromatin precipitations using streptavidin beads (M280, Dynal/Invitrogen, Paisley, UK) were performed in BirA and BirA/bioSTAT5a Ba/F3 cells. The beads were blocked in ChIP dilution buffer with 400 µg/ml yeast RNA and 500 µg/ml BSA. The chromatin was bound to the blocked beads in ChIP dilution buffer overnight at 4°C. Elution from the beads was performed using TE/1% SDS at 65°C.

For double ChIPs or ChIP followed by streptavidin precipitation, the eluate obtained from the first ChIP was used as input in a second ChIP or a chromatin streptavidin precipitation.

Calculation of specific enrichments (fold differences) of STAT5 targets versus input was performed using Real Time PCR according to Litt et al [27] (ChIP/Input = 2^Input Ct-ChIP Ct^). The most enriched (in known STAT5 target sequences) chromatin precipitated DNA was used for library generation (Methods S1 in File S1).

### Short hairpin RNA-mediated knock-down

The lentiviral particles were produced by transient co-transfection of HEK293T cells with the second-generation packaging construct pCMV-ΔR8.91 [28], the VSV-G-envelope plasmid pMDG2 [29] and the specific pLKO.1 plasmid (clone from the TRC1 Library, Sigma) using Lipofectamine 2000 (Invitrogen, Paisley, UK). Nine clones of the TRC1 Library in pLKO.1 vector were used: 1 with scrambled sequence, 4 with sequences specific for *Stat5a* (TRCN0000012549, TRCN0000012550, TRCN0000012551, TRCN0000012552) and 4 with sequences specific for *Stat5b* (TRCN0000012553, TRCN0000012554, TRCN0000012556, TRCN0000012557) (Sigma, St. Louis, MO, USA). Two and three days after transfection, the viral supernatants were collected. Transduction of Ba/F3 cells took place, and 24 h later puromycin (3 µg/ml) was added for selection for 10 days.

### Hematologic samples

The study was conducted in accordance with Helsinki declaration and approved by the Institutional Review Board of University Hospital of Larissa. All subjects gave written informed consent and the procedures followed were in accordance with the Institutional guidelines. 82 patients (male/female: 48/34, mean age: 62.8 years, range: 28–82) were recruited: 17 suffered from AML (acute myeloid leukemia) (11 de novo and 6 after transformation of myelodysplastic syndrome), 4 from ALL (acute lymphoblastic leukemia), 15 from CML (chronic myeloid leukemia) in chronic phase, 26 from JAK2-V617F-positive MPNs (myeloproliferative neoplasms) (including 16 with essential thrombocytosis, 5 with polycythemia vera and 5 with idiopathic myelofibrosis), 4 from JAK2-V617F-negative MPNs (2 with essential thrombocythemia and 2 with idiopathic myelofibrosis) and 16 from CLL. BM (bone marrow) from 4 patients with non-Hodgkin lymphoma (NHL) in complete remission and not currently undergoing therapy, and PB (peripheral blood) from 9 normal individuals were used as controls (male/female: 4/9, mean age: 44.7 years, range: 26-64). For MPNs, the detection of the JAK2-V617F mutation was performed as described [30].

### Reverse Transcription and Real Time PCR

Total RNA was extracted from Ba/F3 cells and human samples [PB (54 patients and 9 controls), BM aspirates (28 patients and 4 controls), granulocytes or monocytes] using Trizol (Invitrogen), treated with RQ1 RNase-Free DNaseI (Promega, Madison, WI, USA) and reverse transcribed with MMLV Reverse Transcriptase (Invitrogen), as described [31], [32]. Real-time PCR was performed with SYBR Green, in ABI PRISM 7000 Sequence Detection System (Applied Biosystems). The amount of template was normalized using primers for *Hprt* and *GAPDH* (Table S1 in File S1) for Ba/F3 cells and hematologic human samples, respectively. The relative quantitation was performed using the ΔΔ^Ct^ method [33].

### Immunofluorescence

Cytospins of PBMCs and granulocytes were blocked with 5% donkey serum (D9663, Sigma, St. Louis, MO, USA) in TBS/Triton 0.1% and incubated overnight at 4°C with anti-p-STAT5 antibody (sc-11761, goat polyclonal, 1:50) or isotype control (Goat IgG, sc-2028) (Santa Cruz Biotechnology). Rabbit anti-goat IgG Alexa 488 was used as secondary antibody (A21222, Molecular Probes, Invitrogen, 1:200).

For co-localization experiments anti-p-STAT5 was used with anti-DPF3 [anti-CERD4 (ab85360, Abcam, Cambridge, UK, 1∶50) or anti-BAF45c affinity purified rabbit polyclonal antibody detecting mouse BAF45c (aa 95-188) [34] (1∶1000)] in 5% donkey serum/TBS/Triton 0.5%. Donkey anti-goat IgG Texas Red (1∶100) and donkey anti-rabbit IgG Cy5 (1∶100) (Jackson ImmunoResearch Laboratories, Inc., West Grove, PA, USA) were used as secondary antibodies.

Following staining, the slides were mounted with Vectashield containing DAPI (Vector Laboratories, Burlingame, CA) and scored using a light Olympus Microscope (BX 40 with U-DO Dual View) and/or a Leica TCS SP5 Confocal Microscope with dual (Tandem) Scanner. Scoring of p-STAT5, DPF3 or both was performed in a blinded manner in granulocytes with p-STAT5 staining in the cytoplasm and/or the nucleus. Granulocytes with low to high intensity p-STAT5 nuclear staining were considered as positive for the nucleus, and with absent p-STAT5 nuclear staining and low to high intensity staining in the cytoplasm were considered positive for the cytoplasm. Granulocytes with low to high intensity DPF3 puncta/staining in the nucleus were considered as positive. For co-localization of DPF3 (red) and p-STAT5 (green) nuclear staining, granulocytes with red and green, and/or yellow puncta were scored. For each sample means and standard deviations were calculated for granulocytes scored in at least three different slides or views of the same slide, depending on granulocytes population (number of scored granulocytes 30-165/sample). Scoring in all samples was confirmed by a second independent user.

### Flow cytometry analysis

Activated STAT5 (p-STAT5) was detected by flow cytometry (Epics XL-MCL, 4 color analysis, Beckman-Coulter, Hialeah, FL, USA) in PB, using a multi-staining protocol. Different cell populations were gated using forward and side light scatter characteristics, and expression of specific markers. The following antibodies were used: anti-p-STAT5 (Tyr694, C71E5, rabbit mAb) (Cell Signaling Technology, Danvers, MA, USA) and anti-CD3 (clone UCHT1, mouse mAb), anti-CD14 (clone RMO52, mouse mAb), anti-CD19 (clone J4.119, mouse mAb), and anti-CD45 (clone J.33, mouse mAb) (Beckman-Coulter, Hialeah, FL, USA). The K562 cell line, characterized by STAT5 activation, served as positive control. PB cells (1×10^5^) were washed in PBS after erythrocyte lysis (Versalyse, BC) and labeled with monoclonal antibodies against CD3 (PE conjugated) or CD19 (PE conjugated) or CD14 (PE conjugated) and CD45 (PE-Cy5 conjugated), in the dark at RmT for 15 min. Afterwards, the cells were fixed in Fix & Perm A buffer (Life Technologies Corporation, Carlsbad, CA, USA) for 15 min, were labeled with anti-p-STAT5 (Alexa Fluor conjugated) at RmT for 15 min in dark, and washed once with PBS before analysis. The percentage of fluorescent cells and the mean fluorescence intensity (MFI) were determined and corrected in each case for background fluorescence using FITC (Alexa Fluor), PE and PE-Cy5-labelled control IgG1 antibodies (Beckman-Coulter).

### Statistical analysis

Student’s t-tests were performed for ChIP, expression analysis in cells and immunofluorescence. Association of the *DPF3* expression levels between controls and disease sub-groups in human samples was estimated using the Mann-Whitney U or the Kruskall-Wallis H non-parametric tests. The differences in p-STAT5 levels in PB subpopulations between CLL patients and controls were estimated using the Mann-Whitney U test. Statistical analyses were performed using SPSS (version 10.0, Chicago, IL, USA).

## Results

### Optimization of ChIPs, library generation and identification of STAT5 target genes

**Figure 1:**
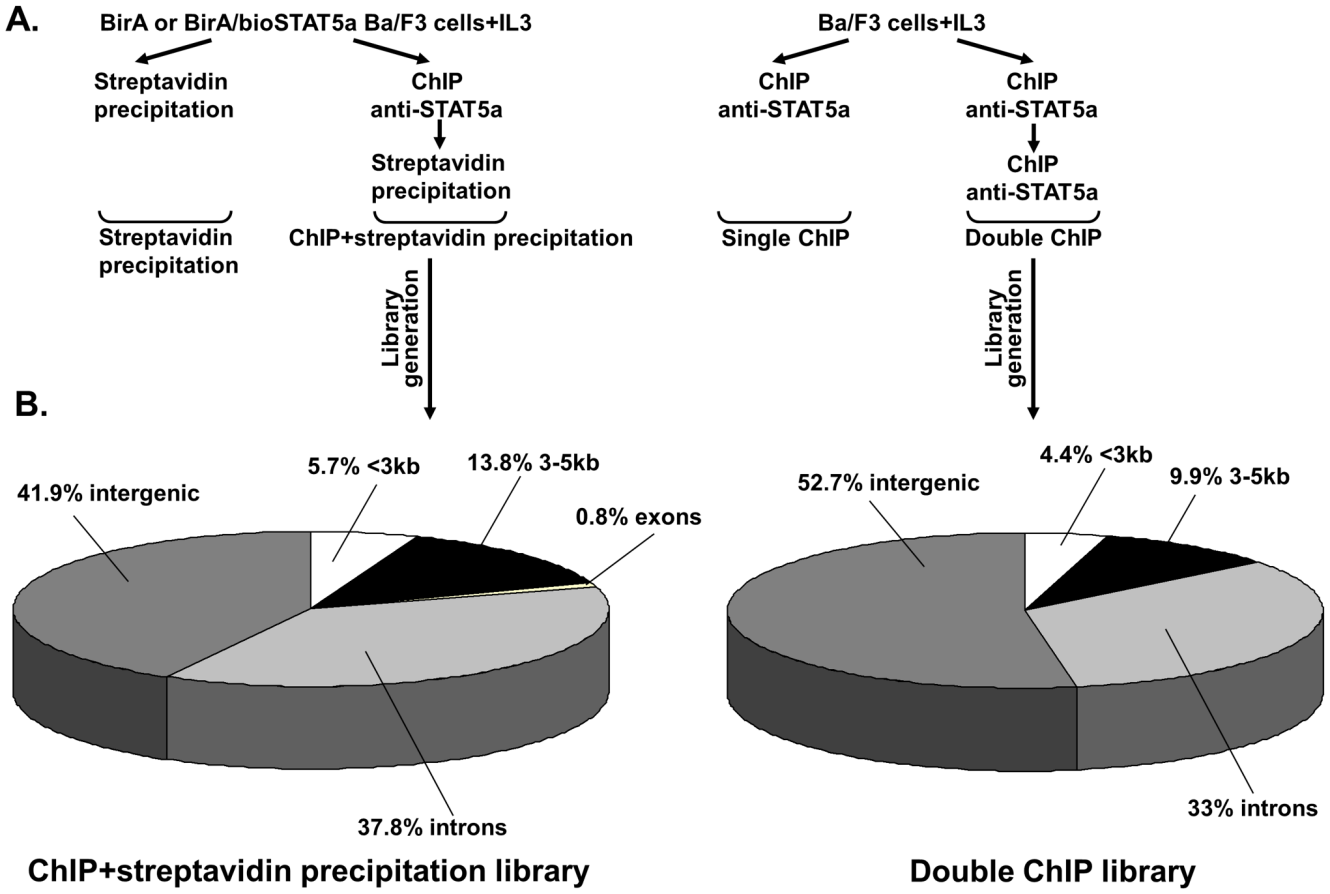
Methodologies and genome distribution of the sequences. **(A) Scheme of the Methodologies followed:** Streptavidin chromatin precipitations and ChIPs followed by streptavidin precipitations were performed in Ba/F3 cells expressing BirA or BirA/bioSTAT5a (left). Single and double ChIPs with anti-STAT5a antibody were performed in Ba/F3 cells (right). All cells were deprived of IL-3 for 6 h and subsequently stimulated with IL-3 for 30 min. **(B) Genome distribution of the sequences:** Genome distribution of the sequences from the ChIP followed by streptavidin precipitation library is shown on the left and from the double ChIP library on the right. The pie charts show the genome distribution of the sequences relative to the nearest neighbor gene, located within <3 kb and 3–5 kb relative to TSS (transcription start site), introns, exons and intergenic regions.

**Figure 2:**
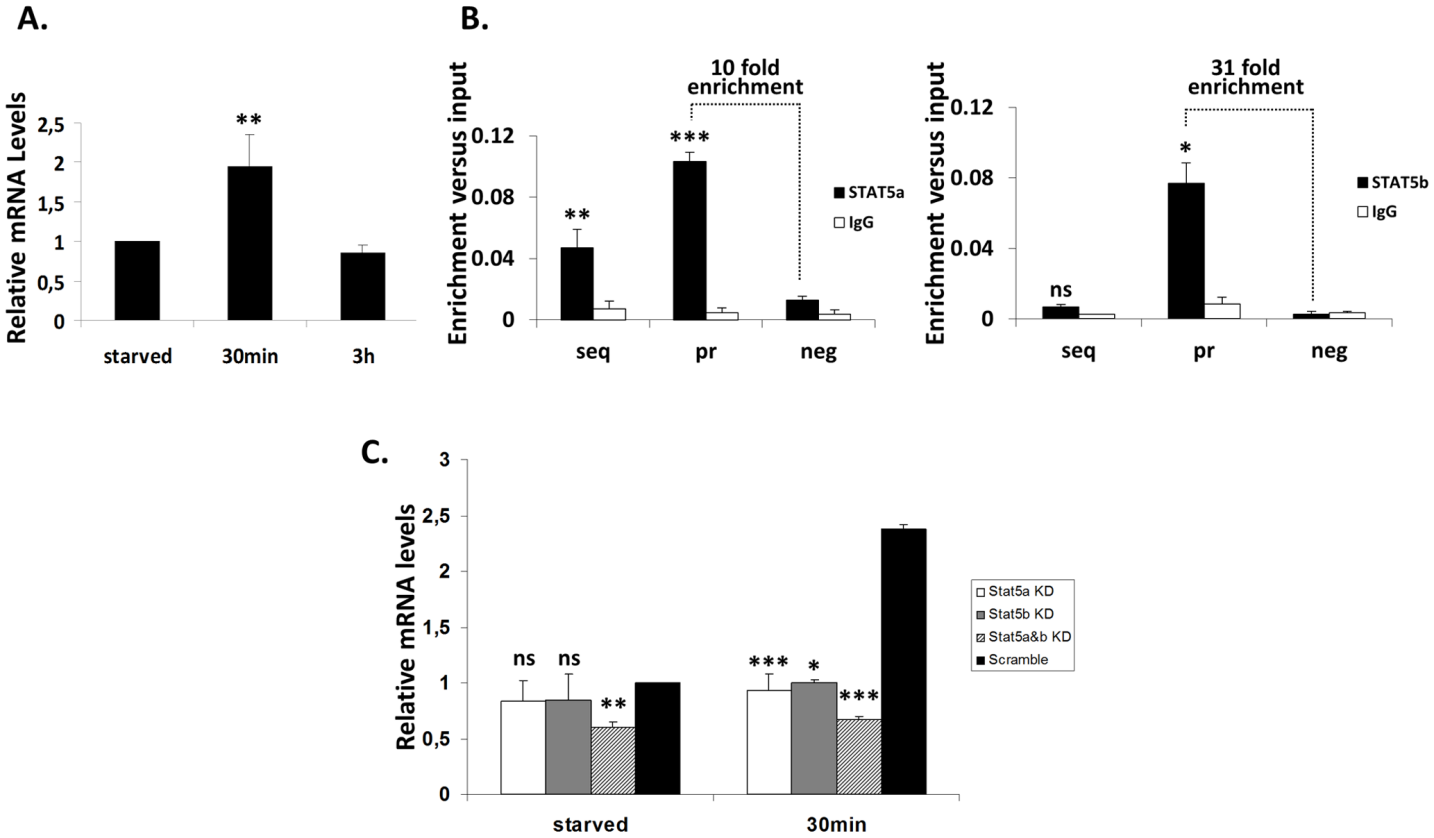
The novel STAT5 target gene *Dpf3:* Expression levels (in wild type and knock-down cells) and STAT5a, STAT5b binding. **(A) Expression levels of *Dpf3*:** Expression levels (mRNA) were measured by real time PCR in Ba/F3 cells deprived of IL-3 for 6 h (starved) and stimulated with IL-3 for 30 min and 3 h. The deprived of IL-3 cells were set as 1. Bars demonstrate mean and standard deviation (SD) values obtained at least in three independent experiments. The statistical significance of the difference in expression between 30 min stimulated and deprived of IL-3 cells is indicated with asterisks (**P = 0.005). **(B)** **STAT5a and STAT5b binding to** ***Dpf3*** **:** Cross-linked chromatin from Ba/F3 cells deprived of IL-3 for 6 h and stimulated with IL-3 for 30 min was used in ChIPs with anti-STAT5a or anti-STAT5b antibody. Three sets of primers were used for each gene. One set specific for the amplification of the isolated genomic region/cloned sequence (seq) containing at least one TTCN_3/4_GAA motif, one set specific for the promoter of each gene (pr) containing at least one TTCN_3_GAA motif and one set of negative control primers (neg) for amplification of a region lacking TTCN_3/4_GAA motifs. IgG was utilized in parallel with anti-STAT5a or anti-STAT5b antibodies, as control. Bars demonstrate mean and SD values of specific enrichments (fold differences) versus input obtained at least in three independent experiments. The statistical significance of enrichment versus the negative control region is indicated with asterisks (ns: not significant, *P = 0.01, **P = 0.002, ***P<0.001). **(C)** **Expression levels of** ***Dpf3*** **in cells with knock-down of STAT5a, STAT5b or both:** Expression levels (mRNA) of *Dpf3* were measured in Ba/F3 cells with knock-down of STAT5a, STAT5b or both. The cells were deprived of IL-3 for 6 h (starved) and stimulated with IL-3 for 30 min. Expression levels were measured by real time PCR and compared with Ba/F3 cells in the deprived of IL-3 state, transduced with scrambled shRNA and set as 1. Bars demonstrate mean and SD values obtained at least in three independent experiments. The statistical significance of the difference in expression between knock-downs and the respective scrambled control is indicated with asterisks (ns: not significant, *P = 0.02, **P = 0.005, ***P<0.001).

We used an IL-3 dependent mouse pro-B cell line (Ba/F3), where the STAT5 signaling cascade is activated upon addition of IL-3. To enrich potential STAT5 targets, we performed single or double ChIPs with anti-STAT5a antibody (in Ba/F3 cells), and chromatin streptavidin precipitations or ChIP followed by chromatin streptavidin precipitations (in Ba/F3 cells stably expressing the bacterial biotin transferase BirA and the biotin-tagged STAT5a) (Figure 1A). Double ChIPs and ChIP followed by streptavidin precipitations resulted in higher enrichments of known STAT5 targets (Results S1 and Figure S1 in File S1) and the respective ChIP DNAs were used for the generation of libraries (Results S1, Sequences S1 and S2 in File S1). STAT5 target genes were selected based on their distance from the sequences, IL-3 induced expression profiles, STAT5 binding, number of STAT5 motifs in the promoter and biological functions (Figure 1B, Figure 2A, B and Table S2, Results S1, Figures S2, S3A in File S1). Here we focused on the *Dpf3* gene, based on its IL-3 induced expression, the strong STAT5 binding to its promoter (described below) and its biological function, as it is known to be a key epigenetic factor. DPF3 protein is a subunit of the BAF chromatin remodeling complex [35] and the *DPF3* gene and/or protein is deregulated in various human malignancies [36]–[38].

### Expression of *Dpf3* gene is induced upon IL-3 stimulation

To investigate if STAT5 is implicated in *Dpf3* function, we tested mRNA levels in Ba/F3 cells deprived of IL-3 for 6 h and subsequently stimulated with IL-3 for 30 min and 3 h. *Dpf3* mRNA levels were increased following IL-3 stimulation with a maximum expression 30 min after IL-3 addition, following the expression pattern of known STAT5 targets (Figure 2A). This suggests that *Dpf3* might be regulated by STAT5 or/and might be involved in STAT5 physiological function.

### STAT5a and STAT5b bind to *Dpf3* gene promoter

ChIPs confirmed the direct binding of STAT5a and STAT5b to the *Dpf3* promoter region and therefore the direct role of both factors in driving *Dpf3* expression. A significant enrichment of STAT5a binding was detected in IL-3 stimulated cells, in the isolated genomic region, as well as the promoter (10 fold enrichment versus a negative control region) and also of STAT5b binding to the promoter region (31 fold enrichment versus a negative control region) (Figure 2B).

ChIPs in Ba/F3 cells deprived of IL-3, demonstrated very weak binding of STAT5a and STAT5b to the promoter and the isolated genomic region, with the enrichment being borderline and much smaller than the one of the IL-3 stimulated state (Figure S3B in File S1).

Analysis of the mouse *Dpf3* promoter region revealed two consensus motifs (TTCN_3_GAA) and one non-consensus, containing one mismatch (TCCN_3_GAA) (Figure S3C in File S1). The non-consensus motif was in a tandem arrangement with one of the two consensus motifs, separated by seven nucleotides, suggesting that cooperation through STAT5 tetramerization might be essential for transcriptional activation of *Dpf3* gene. To further investigate the functionality of these sites to directly bind STAT5 *in vitro*, we performed an EMSA (Figure S3D in File S1), which confirmed a direct *in vitro* binding (Results S1 in File S1).

Taken together the ChIP and EMSA findings confirm *in vivo* and *in vitro*, that STAT5 binds directly to the promoter of the *Dpf3* gene.

### STAT5a and STAT5b down-regulation alters the expression of *Dpf3* gene

To further validate whether *Dpf3* is a STAT5a and STAT5b target gene, we down-regulated STAT5a or STAT5b (Results S1 and Figure S4 in File S1) and assessed *Dpf3* expression levels. In the IL-3 deprived state, knock-down of STAT5a or STAT5b did not significantly influence the expression of *Dpf3* (Figure 2C). When examined 30 min following IL-3 stimulation, knock-down of STAT5a or STAT5b almost completely abolished the IL-3 induced expression of *Dpf3.* At this time point, *Dpf3* gene was down-regulated upon STAT5a knock-down (61% down-regulation versus scrambled shRNA) and STAT5b knock-down (58% down-regulation versus scrambled shRNA) (Figure 2C). Our results suggest that *Dpf3* is positively regulated by STAT5a and STAT5b 30 min following IL-3 stimulation.

To further validate our results, we then generated double STAT5a and STAT5b knock-down cells (Results S1 and Figure S4 in File S1). In the IL-3 deprived state, down-regulation of both STAT5a and STAT5b resulted in a relatively small down-regulation of the expression of *Dpf3* (40.1% down-regulation versus scrambled shRNA). Thirty min following IL-3 stimulation, the *Dpf3* gene was significantly down-regulated (72% down-regulation versus scrambled shRNA) (Figure 2C), further confirming that *Dpf3* is positively regulated by both STAT5a and STAT5b.

### High *DPF3* mRNA levels in CLL

In an effort to understand the mechanisms linking oncogenesis to the interplay of STAT5 activation/regulation of *DPF3* gene, we moved to human pathologic conditions that demonstrate activated STAT5. We investigated *DPF3* mRNA levels in hematologic malignancies and focused on CLL, where the role of STAT5 and its target genes is unexplored.

**Figure 3:**
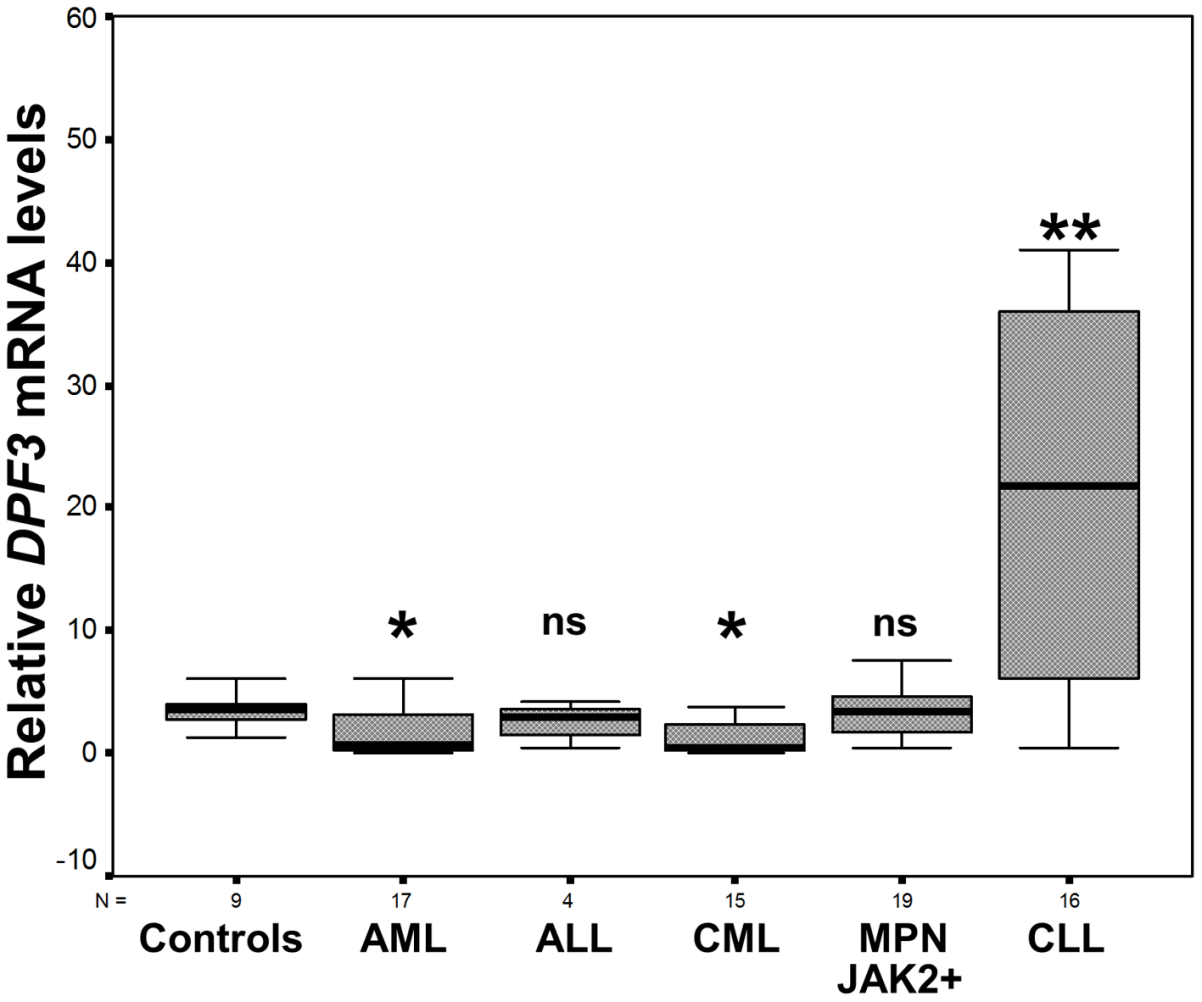
Expression levels of *DPF3* in hematologic malignancies. *DPF3* expression levels (mRNA) in patients with hematologic malignancies [AML (PB and/or BM), ALL (PB and/or BM), CML (PB and/or BM), MPN-JAK2-positive (+) (PB) and CLL (PB)] are shown and compared with the expression in PB of healthy controls. The boxes represent the interquartile range that contains the 50% of values. The whiskers are lines that extend from the box to the highest and lowest values, excluding outliers. A line across the box indicates the median value for each patient cohort. In this graph outliers and extremes have been omitted. P-values versus healthy controls were calculated by the Mann-Whitney U test and are shown in Table 1 (ns: not significant, *0.01≤P<0.05, **P = 0.009).

The Kruskall-Wallis H test revealed a significant difference of *DPF3* mRNA levels between the different disease and control sub-groups analyzed (P<0.001). In particular, CLL patients displayed a significant up-regulation of *DPF3* mRNA levels in PB compared to controls, while AML and CML patients displayed lower *DPF3* mRNA levels compared to its expression in the PB and BM of controls (Table 1, Figure 3). Some patients with AML and CML exhibited a strong down-regulation and others an up-regulation of *DPF3* mRNA levels; however, no clear differences in their clinical-laboratory profiles were observed. All patients with MPNs (both JAK2-V617F-positive and -negative) displayed high *DPF3* mRNA levels, which was more profound in BM compared to PB. However, the differences were not significant (Table 1). Similarly, no significant differences in *DPF3* mRNA levels were observed between ALL patients and controls or between *DPF3* expression and age (P = 0.129).

**Table 1:** Expression of *DPF3* gene in patients with hematologic malignancies.

	No	mRNA levels	*P_1_* valuê	*P_2_* valuê
		Mean ± SE (Range)		
**Healthy controls**				
**PB**	9	3.58±0.47 (1.28–6.08)		
**BM**	4	21.30±10.35 (0.97–46.36)		
**AML (PB and/or BM)**	17	2.77±0.99 (0.02–13.83)	**0.049**	**0.032**
**de novo**	11	2.48±1.22 (0.02–13.83)	**0.037**	**0.037**
**secondary**	6	3.29±1.85 (0.07–11.39)	0.289	0.088
**ALL (PB and/or BM)**	4	2.64±0.77 (0.55–4.19)	0.355	0.149
**CML (PB and/or BM)**	15	1.94±0.70 (0.03–8.39)	**0.016**	**0.021**
**MPN-JAK2-V617F(+)**	26	6.04±1.12 (0.51–22.16)		
**PB**	19	4.06±0.85 (0.51–16.73)	0.806	
**BM**	7	11.43±2.62 (3.21–22.16)		0.571
**MPN-JAK2-V617F(**–**) (BM)**	4	16.41±12.11 (2.91–52.71)		0.773
**CLL (PB)**	16	29.52±7.60 (0.44–93.70)	**0.009**	

Means of *DPF3* expression levels (relative mRNA levels), standard errors (SE) and range for each patient cohort and healthy controls are shown. ?Statistical significance refers to comparison with the expression levels in the PB (P_1_) or BM (P_2_) of healthy controls (Mann-Whitney U test). Statistically significant P values are depicted in bold. Considering that in AML, ALL and CML, a rather similar infiltration of PB and BM by neoplastic cells is observed, a comparison with both groups of healthy controls (PB and BM) was performed.

### High DPF3 expression in CLL is linked with increased STAT5 activation in non-malignant myeloid cells (granulocytes)

**Figure 4:**
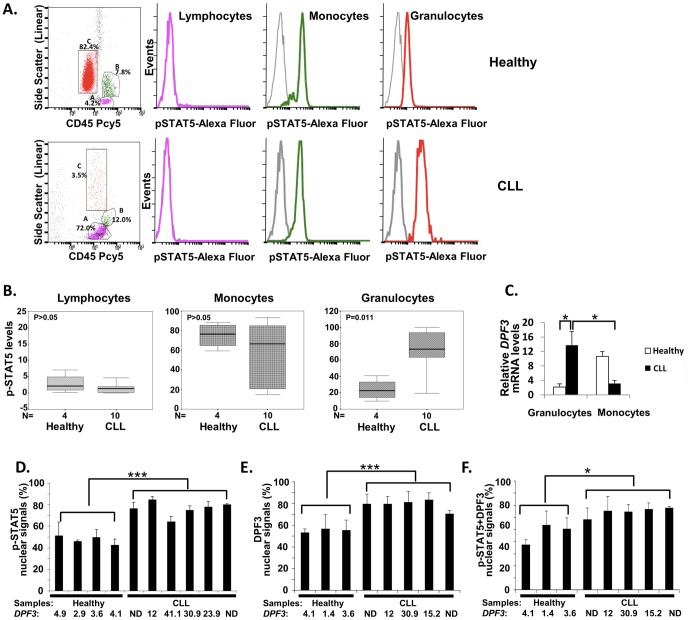
Detection of activated STAT5 (p-STAT5), DPF3 staining and mRNA levels in CLL. **(A) Flow cytometry analysis:**Indicative plots of peripheral blood cells analysis are shown (healthy individual with low *DPF3* expression levels (3.6) in upper panels and CLL patient with high *DPF3* expression levels (19.69) in lower panels). Dot plots on the left indicate percentages of gated cells used on the analysis (A: lymphocytes in pink, B: monocytes in green and C: granulocytes in red). Histograms on the right indicate p-STAT5 levels in lymphocytes (pink), monocytes (green) and granulocytes (red). Isotype control (IgG1) is depicted in grey. **(B) Levels of p-STAT5 in the peripheral blood cell subpopulations analyzed by flow cytometry:** The boxes represent the interquartile range containing 50% of values in lymphocytes, monocytes and granulocytes of healthy individuals (n = 4) and CLL patients (n = 10). The whiskers are lines that extend from the box to the highest and lowest values, excluding outliers. A line across the box indicates the median value for each group. The statistical significance of difference is noted on the charts. **(C)** ***DPF3*** **expression levels (mRNA) in granulocytes and monocytes:** *DPF3* expression levels were measured in granulocytes and monocytes of CLL patients (n = 5) and healthy individuals (n = 4) by real time PCR. Bars demonstrate mean and standard error of the mean (SEM) values of expression. The statistical significance of the differences in expression is indicated with an asterisk (*0.01≤P<0.05). **(D) Immunofluorescence detection of nuclear p-STAT5:** Granulocytes with low to high intensity nuclear p-STAT5 staining were scored as positive for the nucleus. Granulocytes with absent nuclear staining and low to high intensity cytoplasmic staining were considered positive for the cytoplasm (***P<0.001). **(E) Immunofluorescence detection of nuclear DPF3:** Scoring of nuclear DPF3 staining (detected as puncta in the nucleus) was performed on granulocytes positive for p-STAT5 staining in the cytoplasm and/or the nucleus. Granulocytes with low to high intensity puncta in the nucleus were considered positive (***P<0.001). **(F) Immunofluorescence detection of both nuclear p-STAT5 and DPF3:** For co-localization of DPF3 (red) and p-STAT5 (green) nuclear staining, granulocytes with green and red, and/or yellow staining puncta (representing p-STAT5 and DPF3 co-localization) were scored (*P = 0.05). In D, E and F: Scoring was performed in granulocytes showing p-STAT5 staining in the nucleus, the cytoplasm or both; bars demonstrate mean and SD values obtained at least in three different slides or regions of the same slide; the statistical significance of the difference between samples with low (healthy individuals) and high (CLL patients) *DPF3* expression is shown; *DPF3* expression levels (mRNA) are shown below the samples; ND: not determined.

To explore the interplay and link/correlation between STAT5 activation and DPF3 gene regulation/expression in CLL, we performed flow cytometry experiments in PB of CLL patients and healthy individuals. Using this analysis, we found evidence of STAT5 activation in PB cell subpopulations. In healthy individuals, p-STAT5 expression was observed mainly in monocytes and to a lesser extent in granulocytes, while lymphocytes were characterized by little or absent p-STAT5 expression (Figure 4A, B). Interestingly, patients with CLL displayed a significant increase of p-STAT5 in granulocytes compared to controls (mean±SD: 72.1±24.1% versus 23.9±13.1%, P = 0.011), while its expression in monocytes and lymphocytes was similar to controls (mean±SD: 58.2±30.1% versus 75.1±12.9%, P = 0.480, and 1.9±2.3% versus 2.9±2.9%, P = 0.320, respectively) (Figure 4B).

To further confirm whether STAT5 activation is linked with high *DPF3* expression, we measured *DPF3* mRNA levels in monocytes and granulocytes from CLL patients and healthy individuals. A significant 6.5 fold up-regulation of *DPF3* expression was observed in granulocytes of CLL samples versus healthy controls. Furthermore, *DPF3* expression was up-regulated more than 4 fold in granulocytes versus monocytes in the CLL samples (Figure 4C), which confirmed that STAT5 activation is linked with high *DPF3* expression in granulocytes.

As active p-STAT5 translocates from the cytoplasm to the nucleus to tightly regulate transcription, we used immunofluorescence to further evaluate whether high *DPF3* expression correlates with high STAT5 activation/nuclear p-STAT5 localization (Figure 4D, E, F and Figure S5 in File S1). We detected an increased percentage of nuclear p-STAT5 staining in granulocytes of CLL patients with higher *DPF3* expression versus healthy individuals with lower *DPF3* expression (Figure 4D). We also scored granulocytes for DPF3 immunostaining to investigate whether the increase in *DPF3* gene expression/p-STAT5 activation is linked also with an increase in the percentage of granulocytes with DPF3 staining. We observed an increase in positive DPF3 nuclear immunostaining in CLL patients (Figure 4E). We then counted cells with positive nuclear immunostaining for both p-STAT5 and DPF3 and observed higher percentages of granulocytes with nuclear staining in CLL patients compared to healthy controls (Figure 4F).

Taken together these data provide evidence of a significant increase of STAT5 activation in granulocytes of CLL patients. Higher *DPF3* expression levels were linked to increased STAT5 activation/nuclear p-STAT5 and DPF3 localization.

### STAT5 binds strongly to the promoter of human *DPF3* in CLL granulocytes

**Figure 5:**
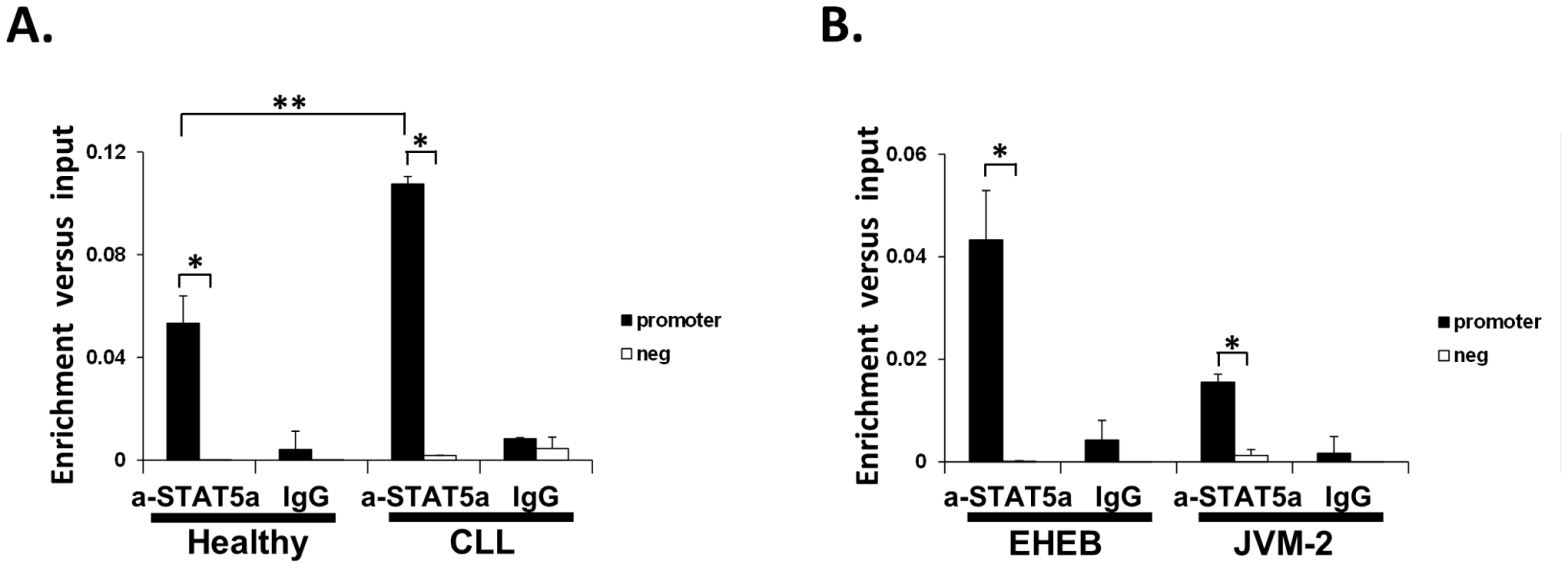
STAT5 binding to human *DPF3* promoter. **(A) STAT5a binding to human *DPF3* promoter in granulocytes:** Cross-linked chromatin from granulocytes of a CLL patient and a healthy individual was used in ChIPs with anti-STAT5a antibody. **(B) STAT5a binding to human** ***DPF3*** **promoter in CLL cell lines:** Cross-linked chromatin from EHEB and JVM-2 cells was used in ChIPs with anti-STAT5a antibody. In A and B, IgG was utilized in parallel with anti-STAT5a antibody, as control. Two sets of primers were used: One set specific for the amplification of the *DPF3* promoter containing a STAT5 motif and one set of negative control primers (neg) for amplification of a region lacking STAT5 motifs. Bars represent mean and SD values of specific enrichments (fold differences) versus input obtained at least in three independent experiments. The statistical significance of enrichment for promoter sequences versus the negative control region (*0.01≤P<0.03) and for patient versus healthy individual (**P = 0.007) is indicated; a-STAT5a: anti-STAT5a.

To investigate the mechanisms underlying the increased *DPF3* expression in CLL, we performed ChIPs in granulocytes, total PBMCs, and CLL cell lines to evaluate *in vivo* binding of STAT5 to the promoter of *DPF3*. Motif analysis of the human *DPF3* promoter revealed two STAT5 consensus motifs (TTCN_3_GAA) and one non-consensus motif (TTCN_3_GCA) (Figure S6 in File S1). ChIPs in granulocytes from a CLL patient with high *DPF3* mRNA levels (83.51) and a healthy individual with low *DPF3* (3.85) mRNA levels showed that STAT5 was bound to the promoter of *DPF3* in both samples; however the enrichment was significantly higher in the CLL patient (Figure 5A). ChIPs in total PBMCs confirmed this finding and demonstrated higher enrichment in the CLL patient in comparison to the healthy individual (data not shown).

We then used two human chronic B cell leukemia EBV-transformed cell lines (ΕΗΕΒ and JVM-2) to further investigate whether STAT5 binding to the promoter of *DPF3* gene was less efficient in these cases. ChIPs demonstrated binding of STAT5 to the promoter of *DPF3* gene in both cell lines (Figure 5B), with the enrichment being lower than the one detected in granulocytes, as expected based on the low STAT5 activation in lymphocytes detected by FACS.

Taken together these results provide further evidence of a direct role of STAT5 in the regulation of human *DPF3* gene via binding to its promoter and suggest that the increased *DPF3* expression in PBMCs and granulocytes of CLL patients is the result of an increase in STAT5 binding to the promoter of *DPF3.*

## Discussion

Our study describes modifications of the classical ChIP protocol introducing two sequential antibody affinity steps and the combination of ChIP with chromatin streptavidin precipitation, after applying *in vivo* biotinylation method [39], [40], resulting in higher enrichments of known STAT5 targets in comparison to the single step ChIP. Thus, these methods can be applied successfully in future studies for genome-wide identifications of STATs and other transcription factor target genes.

The induction of expression of *Dpf3* and the other STAT5 target genes identified showed similarities to the ones reported for other known STAT5 target genes [13], suggesting a potential involvement in STAT5 physiological function. ChIPs and EMSAs confirmed STAT5 binding to the *Dpf3* promoter, and our knock-down experiments demonstrated that *Dpf3* gene is positively regulated by STAT5a and STAT5b, 30 min following IL-3 stimulation.

The novel STAT5 target gene *Dpf3*, identified here, is known to be a key epigenetic factor. DPF3 protein is a subunit of the BAF chromatin remodeling complex and is characterized by a double PHD finger, which interacts with acetylated and methylated histone tail residues [35]. In cancer, there is a selective pressure for alterations in SWI/SNF complex function and in the expression of various BAF subunits of SWI/SNF [41]. Multiple observations point towards a role of various BAF subunits in tissue-specific tumor prevention [41], opening the way for investigations on the functional role of other subunits, including DPF3 (BAF45C). The SWI/SNF complex was also shown to interact with STAT5 [42].

The *DPF3* expression analysis in hematologic malignancies with activated STAT5 presented here was performed in an effort to understand the mechanisms linking oncogenesis to the interplay of STAT5 activation and regulation of its target genes. These mechanisms in CLL are unexplored. Constitutive activation of STAT5 has been associated with hematologic and solid malignancies [4], [43], [44], leading to deregulation of its target genes. In this study, we observed *DPF3* expression in the majority of hematologic malignancies analyzed. Moreover, we identified expression of *DPF3* in healthy controls, which possibly relates to STAT5 activation participating in the proliferation, maturation and survival of normal progenitor cells and/or lymphocytes [45].

Until now the role of STAT5 and its targets genes in CLL has not been explored. We now show, for the first time, up-regulation of *DPF3* expression and STAT5 activation in myeloid cells of CLL patients. STAT5 activation has been reported in CLL cells after exposure to IL-15, resulting in malignant cell proliferation and inhibition of apoptosis [46]. However, we demonstrated an increased *DPF3* expression in CLL patients that was linked with a significant increase of STAT5 activation in myeloid lineage cells (granulocytes) and not in neoplastic B cells. The reasons for this lineage bias remains to be further elucidated in future studies. Various reports have highlighted the close communication of myeloid and tumor-lymphoid cells, as well as the contribution of myeloid cells in the proliferation and survival of neoplastic cells in B cell lymphoproliferative disorders [47], [48]. Moreover, cytokines (such as APRIL) produced by neutrophils in patients with lymphoproliferative disorders increase tumor aggressiveness [48], while others (i.e. IFN-γ, -*α*, IL-4, -8) can inhibit apoptosis of malignant B cells *in vitro* [49]–[52]. These cytokines may be released by the leukemic cells to exert an autocrine control of CLL cell survival, or by accessory non-malignant leukocytes, which produce regulatory signals for modulating the survival of leukemic cells. In this context, monocytes and NK cells were also found to inhibit spontaneous apoptosis of leukemic B cells [53]. How monocytes, granulocytes and NK cells control CLL survival is largely unknown. Our findings open novel routes of investigation to facilitate the understanding of the role of granulocytes in important processes (i.e. proliferation, cell cycle, apoptosis) of CLL cells. Whether granulocytes produce cytokines or other factors capable of modulating such processes, and whether STAT5 activation/regulation of target genes is involved in these processes remains to be investigated in future studies. Our preliminary results show that CLL granulocyte culture supernatants alter proliferation and apoptosis of CLL cell lines in comparison to healthy supernatants (data not shown). Such experiments in cell lines, in combination with primary CLL cell cultures will shed light on the role of granulocytes and activated STAT5 in CLL.

Among the cytokines produced by myeloid cells contributing to the homeostasis of normal B cells and possibly participating in the survival, proliferation and apoptosis of malignant CLL cells, APRIL plays a pivotal role. APRIL was found elevated in CLL patients [54], [55], can be produced by granulocytes in PB and interestingly we identified STAT5 motifs in its promoter/first intron (data not shown). If there is a direct link between STAT5 activation in non-malignant myeloid cells and high expression of APRIL in CLL patients it still remains to be determined.

Overall, our hypothesis for future testing speculates that activated STAT5 in non-malignant myeloid cells by differential binding on target genes, might tightly control regulatory signals and secretion of factors/cytokines, which are crucial for the proliferation, apoptosis, cell cycle or other biological processes of the leukemic cells.

Until now, the contribution of both STAT5 activation and *DPF3* expression in patients with CLL was completely unknown and for the first time our study highlights the up-regulation of a STAT5 target gene and activation of STAT5 pathway in myeloid cells of CLL patients. Our findings open novel routes of investigation towards the understanding of the mechanisms inducing STAT5 signaling in myeloid cells, the role of STAT5 activation in the interplay between non-malignant myeloid and malignant B cells and the functions of STAT5 target genes networks in CLL biology.

## Supporting Information

File S1 **Supporting Information (Methods, Results, Discussion, References, Tables, Figures and Sequences). Methods S1:** Library generation and analysis of sequences. Electrophoretic mobility shift DNA binding assays. Immunoblot Analysis. **Results S1:** Comparison of the methods used for optimization of ChIP and library generation. Comparison of the libraries generated for STAT5 target genes identification. Induction of expression of potential STAT5 target genes upon IL-3 stimulation. Confirmation of STAT5 binding to the selected target genes. Assessing the efficiency of STAT5 knock-downs. **Discussion S1. References S1. Table S1:** Primers-oligos used in the study. **Table S2:** Selected potential STAT5 target genes. **Figure S1:** Comparisons of efficiency of the methodologies used. **Figure S2:** Expression levels of selected STAT5a target genes. **Figure S3:** STAT5 binding to the novel target genes. **Figure S4:** Efficiency of STAT5a and STAT5b knock-downs. **Figure S5:** Immunofluorescence detection of activated STAT5 (p-STAT5) and DPF3 in CLL. **Figure S6:** Sequence of the human *DPF3* promoter. **Sequences S1:** Sequences from the ChIP followed by streptavidin precipitation library. **Sequences S2:** Sequences from the double ChIP library.
(DOCX)
