# Supplementary material for: Identification of a STAT5 Target Gene, Dpf3, Provides Novel Insights in Chronic Lymphocytic Leukemia
Source: PLoS One. 2013 Oct 14;8(10):e76155. doi: 10.1371/journal.pone.0076155 (PMC3796511; doi:10.1371/journal.pone.0076155)
Supplement: File S1 — Supporting Information (Methods, Results, Discussion, References, Tables, Figures and Sequences). Methods S1: Library generation and analysis of sequences. Electrophoretic mobility shift DNA binding assays. Immunoblot Analysis. Results S1: Comparison of the methods used for optimization of ChIP and library generation. Comparison of the libraries generated for STAT5 target genes identification. Induction of expression of potential STAT5 target genes upon IL-3 stimulation. Confirmation of STAT5 binding to the selected target genes. Assessing the efficiency of STAT5 knock-downs. Discussion S1. References S1. Table S1: Primers-oligos used in the study. Table S2: Selected potential STAT5 target genes. Figure S1: Comparisons of efficiency of the methodologies used. Figure S2: Expression levels of selected STAT5a target genes. Figure S3: STAT5 binding to the novel target genes. Figure S4: Efficiency of STAT5a and STAT5b knock-downs. Figure S5: Immunofluorescence detection of activated STAT5 (p-STAT5) and DPF3 in CLL. Figure S6: Sequence of the human DPF3 promoter. Sequences S1: Sequences from the ChIP followed by streptavidin precipitation library. Sequences S2: Sequences from the double ChIP library. (DOCX) [file pone.0076155.s001.docx]

**File S1**

**Supporting Information (Methods, Results, Discussion, References, Tables, Figures and Sequences)**

**Identification of a STAT5 target gene, *Dpf3*, provides novel insights in chronic lymphocytic leukemia**

**Marina Theodorou, Matthaios Speletas, Antigoni Mamara, Georgia Papachristopoulou, Vassiliki Lazou, Andreas Scorilas and Eleni Katsantoni**

**Methods S (Supplemental Methods)**

***Library generation and analysis of sequences***

Chromatin precipitated DNA was digested with Sau3AI (New England Biolabs, Beverly, MA, USA) and ligated in the BamHI site of the pBluescript II SK+ vector (Stratagene, La Jolla, CA, USA). Libraries were transformed in DH10BT phage resistant electrocompetent cells (Invitrogen, Paisley, UK) and random clones were selected and sequenced with an ABI PRISM 3100 Genetic Analyzer (Applied Biosystems) using universal primers. The sequences were analyzed using Genomatix (MatInspector, http://[www.genomatix.de](http://www.genomatix.de)) and/or TESS (http://www.cbil.upenn.edu/cgi-bin/tess/tess) for the detection of STAT5/STAT motifs. Mapping to the mouse genome (NCBI build 36, 37, Ensembl version 47) was performed with TF Target Mapper [[1](#_ENREF_1)] and with Blast/Blat of Ensembl (http//[www.ensembl.org](http://www.ensembl.org)) and/or UCSC (http://genome.ucsc.edu/). Repeat Masker (http://[www.repeatmasker.org](http://www.repeatmasker.org)) detected repetitive elements within the sequences. The genes in a region 100kb upstream and downstream of each sequence were extracted and their functions were analyzed in various databases including Ingenuity (http://[www.ingenuity.com](http://www.ingenuity.com)) and Gene Cards (<http://www.genecards.org/>). GENECODIS (http://genecodis.cnb.csic.es/analysis) [[2](#_ENREF_2)] was used to analyze GO (Gene Ontology) annotations in the gene lists of different ChIP strategies.

***Electrophoretic mobility shift DNA binding assays***

Complementary annealed oligonucleotides (Table S1) were labeled with T4 Polynucleotide kinase (New England Biolabs) using γ^32^P-ATP, according to manufacturer’s instructions. The labeled oligonucleotides were separated from unincorporated radionucleotides by passing through a G-25 column. Binding reactions were performed in 12% glycerol, 20 mM Tris-HCl (pH 8), 1 mM EDTA, 1 mM DTT, 100 mM KCl, 300 μg/ml BSA and 0.5 μg poly dI/dC (27-7880-03, Pharmacia) for 20 min at RmT (room temperature). STAT5 recombinant protein (S56-54H, SignalChem, Richmond, BC, Canada) was added last to the binding reaction after addition of the labeled oligonucleotide. For cold competition experiments, a 5-fold and 10-fold molar excess of cold competitor (unlabeled oligonucleotide) was used. For supershift experiments, 2 μl of anti-STAT5a antibody (sc-1081X, Santa Cruz Biotechnology, Inc., Santa Cruz, CA, USA) was added to the bandshift reaction for 20 min after addition of the recombinant protein. Reactions were run in a non-denaturing 6% acrylamide gel in 0.5X TBE for 2 hours at 300 V. After drying, the gels were exposed in a Phosphorimager cassette and then scanned on a Typhoon Scanner (Amersham/Molecular Dynamics).

***Immunoblot Analysis***

Total cell extracts (≥5 μg/lane) (from STAT5a and STAT5b knock-down cells) were prepared with whole cell lysis buffer [10mM Tris-HCl, 150 mM NaCl, 1% Triton X-100, 1 mM Na_3_VO_4_, 10 mM NaF, 5 mM EDTA, 1 mM PMSF and ‘complete’ protease inhibitor cocktail (Roche)] for 30 min at 4^o^C. Extracts were resolved by SDS/PAGE in an 8% gel and blotted onto Immobilon-P membrane (Millipore, Bedford, MA, USA). Membranes were blocked for 1 h in 5% milk/1 x TBS/0.05% NP40*,* incubated overnight at 4C with anti-STAT5a (L-20) rabbit polyclonal antibody (Santa Cruz Biotechnology, sc-1081, 1:1000), anti-STAT5b (G-2) mouse monoclonal antibody (Santa Cruz Biotechnology, sc-1656, 1:1000) or anti-β-actin mouse antibody (A2228, Sigma, St. Louis, MO, USA, 1:1000), washed in 1 x TBS/0.5 M NaCl/0.3% Triton X-100 and incubated with secondary anti-rabbit or anti-mouse HRP conjugated antibodies, respectively. Bands were detected using enhanced chemiluminescence (Amersham Pharmacia, Uppsala, Sweden). Densitometric quantitation of the signals was performed with Quantity One Software (Biorad).

**Results S (Supplemental Results)**

***Comparison of the methods used for optimization of ChIP and library generation***

We used an IL-3 dependent mouse pro-B cell line (Ba/F3 cells), where the STAT5 signaling cascade is activated upon addition of IL-3. We performed and optimized different ChIP protocols (Figure 1A) to obtain high enrichments of STAT5 target sequences. Chromatin streptavidin precipitations and ChIP followed by chromatin streptavidin precipitations were performed in BirA/bioSTAT5a Ba/F3 cells, where the BirA ligase recognized and specifically biotinylated the bio-tagged STAT5a protein. The biotin, by virtue of its tight binding to streptavidin, provided a sensitive affinity tag for the purification of target genes associated with biotinylated STAT5a. Details on the generation of BirA/bioSTAT5a cells and the efficiency of biotinylation will be presented elsewhere (manuscript in preparation).

We first compared the efficiency of the different ChIP protocols used. By PCR we verified the enrichment of known STAT5 target genes (*BclX*, *Id1* and *Cis*) in the chromatin precipitated DNA (data not shown). We observed significant binding of STAT5a to the promoters of the known target genes used, but not in a negative control region lacking STAT5 motifs. The specificity of STAT5a binding was also verified by the use of rabbit IgG in all ChIP experiments. BirA expressing Ba/F3 cells were used as a negative control in streptavidin precipitations and in ChIPs followed by streptavidin precipitations, and showed absent or lower binding in comparison to the BirA/bioSTAT5a expressing Ba/F3 cells.

To quantitate the efficiency of each method and allow further comparisons, we calculated the enrichments of the chromatin precipitated DNAs versus input for *BclX*, *Cis* and *Socs3* target sequences by Real Time PCR (Figure S1). For *BclX* the highest enrichment was observed using ChIP followed by streptavidin precipitation, whereas for *Cis* and *Socs3* the highest enrichment was seen using double ChIP. Single ChIPs or streptavidin precipitations exhibited lower enrichments. Enrichments of DNAs immunoprecipitated with IgG, streptavidin precipitated DNAs of BirA control cells or negative control regions were very low or absent. Thus we concluded that the highest enrichment of known STAT5 target sequences/genes is obtained using double ChIP and ChIP followed by streptavidin precipitation. We subsequently used these samples for the generation of libraries. To ensure equal representation of all sequences and minimize bias in library generation, we did not amplify the chromatin precipitated DNA by PCR, but performed direct digestions with a common restriction enzyme present in the mouse genome (Sau3AI) and cloning in the vector pBluescript.

***Comparison of the libraries generated for STAT5 target genes identification***

Random library clones were sequenced and then aligned automatically with the mouse genome using TF Target Mapper [[1](#_ENREF_1)] and/or with BLAST/BLAT of Ensembl or UCSC. In total 357 sequences were analyzed. 175 sequences were derived from ChIP followed by streptavidin precipitation (Sequences S1) and 182 sequences from double ChIP experiments (Sequences S2). To compare the libraries and the efficiency of the two methods used, we searched the sequences for the presence of STAT/STAT5 motifs, repetitive elements and vicinity to genes.

The detection of GAS (INF-γ-activated sequence) motifs [for STAT5 (TTCn_3_gaa) and other STAT (ttCn_3/4_GAA)] was performed with MatInspector (Genomatix). In a high percentage of our sequences we detected the ttCn_3/4_GAA motif [[3](#_ENREF_3),[4](#_ENREF_4)]. ChIP followed by streptavidin precipitation isolated similar percentage of sequences with ttCn_3/4_GAA motifs (44%), compared to double ChIP (39%). The remaining sequences did not contain any ttCn_3/4_GAA motifs, indicating that STAT5a is bound to these sequences via other factors or that the sites were lost by Sau3AI digestion before library generation.

With Repeat Masker, we checked for the presence of repetitive elements in our sequences. Some sequences contained one or more repetitive elements (partly or fully covering the sequences) and this percentage was higher in ChIP followed by streptavidin precipitation (54.8% out of which 31.4% contained partly and 23.4% fully one or more repetitive elements) than in double ChIP (40.6% out of which 22.5% contained partly and 18.1% fully one or more repetitive elements). Sequences containing fully repetitive elements were excluded from further analysis for target genes in the vicinity.

ChIP followed by streptavidin precipitation isolated slightly more sequences (19.5%) closer to genes (≤5kb) than double ChIPs (14.3%) (Figure 1B). The remaining sequences were located in introns and intergenic regions (Figure 1B) confirming the notion that STAT5, like other transcription factors, might regulate transcription from distance.

Furthermore, using GENECODIS [[2](#_ENREF_2)] we analyzed GO annotations that co-occur in the gene lists corresponding to the different experimental strategies applied (ChIP/streptavidin precipitations and double ChIP experiments). This revealed several common annotations (such as regulation of transcription, DNA-dependent (BP) and protein binding (MF)) corresponding in the most frequent and larger groups of genes, in both gene lists (data not shown).

Taken together these results suggest that both methods efficiently isolated sequences that represent potential STAT5 targets.

We then focused on genes located up to 15kb upstream and downstream of the sequences. Our findings concerning 10 selected genes are presented here (Table S2). Analysis with Genomatix/MatInspector of the corresponding cloned sequences showed that all contained ttCn_3/4_GAA motifs. ttCn_3_GAA motifs were also detected in a region 4kb upstream of the transcription start site (TSS) containing the promoter of each gene, with the only exception being *Smarcc2* (Table S2).

To further confirm that the cloned sequences corresponding to the 10 selected genes are true STAT5 targets, we used PCR to test their presence in both libraries generated (from ChIP followed by streptavidin precipitation and from double ChIP). Primers specific for amplifying the cloned sequences were used and verified their presence in both libraries (data not shown).

***Induction of expression of potential STAT5 target genes upon IL-3 stimulation***

To investigate if STAT5 is implicated in the physiological function of the selected target genes, we first assessed their expression levels in Ba/F3 cells deprived of IL-3 for 6 h and subsequently stimulated with IL-3 for 30 min and 3 h (Figure 2A, Figure S2). *Dpf3*, *Lrg1*, *Rnf41*, *Traf1* and *Mta3* were significantly induced upon IL-3 stimulation with a maximum level of expression 30 min after stimulation and a decrease in expression 3 h after stimulation. This follows the expression pattern of other known STAT5 target genes and suggests that these genes might be regulated by STAT5. *Jmjd1a* and *Smarcc2* expression showed a marginal, but not significant, induction in cells stimulated for 30 min, followed by a decrease. Three of the genes tested (*Ddr2*, *Glis3* and *Pdgfc*) were not expressed or were expressed below the detection limit of our Real Time PCR method and, thus, were excluded from further analysis.

***Confirmation of STAT5 binding to the selected target genes***

We performed ChIPs to confirm the direct binding of STAT5a and therefore the direct role of STAT5a in driving the expression of the selected genes. We detected significant enrichments of STAT5a binding versus input, for the isolated genomic regions and/or the corresponding promoters of *Dpf3*, *Lrg1*, *Mta3*, *Rnf41* and *Traf1* genes (Figure 2B, Figure S3A). *Jmjd1a* and *Smarcc2* did not show a significant enrichment of the isolated genomic regions and/or the corresponding promoters, when compared to negative control regions (data not shown) and thus were excluded from further testing. *Lrg1, Mta3* and *Rnf41* exhibited the highest enrichment within the isolated genomic regions (cloned sequences) and *Traf1* within both the cloned sequence and the promoter. *Dpf3* gene was selected for further testing, as it demonstrated the most significant binding of STAT5 in the promoter region, as well as a significant increase in IL-3 induced expression.

To further investigate if the STAT5 motifs in the *Dpf3* promoter directly bind STAT5 *in vitro*, we designed oligonucleotides containing wild type or mutated motifs (Table S1, Figure S3C) and performed EMSA assays (Figure S3D). Oligonucleotide 1 (containing one consensus motif) formed a complex with STAT5 recombinant protein, which was not detected in the presence of an excess of unlabeled oligonucleotide 1 competitor, thus confirming the specificity of STAT5-specific DNA binding site interaction. Upon addition of STAT5a antibody, a supershift of the ternary complex was detected (Figure S3D). Oligonucleotide 2 (containing a nonconcensus, and one consensus motif) formed two complexes with STAT5 recombinant protein, potentially indicating complexes with STAT5 monomer, dimer or tetramer (Figure S3D). Both complexes were not detected in the presence of an excess of unlabeled oligonucleotide 2 competitor. Overall, the EMSA data confirm that STAT5 binds directly to the promoter of *Dpf3* *in vitro*.

***Assessing the efficiency of STAT5 knock-downs***

We tested 4 shRNA clones for STAT5a and 4 clones for STAT5b. We used Ba/F3 cells transduced with the most efficient clones A0 and B3, which demonstrated the highest down-regulation of *Stat5a* (66%) and *Stat5b* (83%) expression respectively, 30 min after IL-3 stimulation, as quantitated by real time PCR and confirmed by Western blot (Figure S4-A, B, C, D). The selected Ba/F3 cells transduced with the clones A0 or B3 showed also down-regulation of *Stat5a* (65%) or *Stat5b* (78%) expression in the deprived of IL-3 state, as quantitated by real time PCR and confirmed by Western blot (Figure S4-G, H, I, J).

The double STAT5a and STAT5b knock-down cells were generated by transducing STAT5b knock-down cells (transduced with clone B3) with viral supernatant of STAT5a knock-down cells (transduced with clone A0). Down-regulation of *Stat5a* (59%) and *Stat5b* (74%) 30 min after IL-3 stimulation and of *Stat5a* (65%) and *Stat5b* (71%) in the deprived of IL-3 state was quantitated by real time PCR and confirmed by Western blot (Figure S4-E, F, K, L).

**Discussion S (Supplemental Discussion)**

Our study shows that modifications of the classical ChIP protocol, by introducing two sequential antibody affinity steps (double ChIPs) or the combination of ChIP with chromatin streptavidin precipitation (ChIP followed by streptavidin precipitation), following *in vivo* biotinylation [[5](#_ENREF_5)], resulted in higher enrichments of genuine STAT5 targets. This is accompanied by a decrease in non-specific, background sequences in comparison to the single step ChIP or chromatin streptavidin precipitation. Both methods isolated with almost similar efficiency sequences with STAT5/STAT motifs in close proximity to genes. Slightly more sequences with STAT motifs adjacent to genes were isolated using ChIP followed by streptavidin precipitation experiments, confirming the potential of the combination of the two methods for efficient identification of target genes. A high percentage of the sequences contained the general TTCN_3/4_GAA motif [[4](#_ENREF_4),[6](#_ENREF_6)] confirming the broad repertoire of potential STAT5 motifs and the existence of natural suboptimal GAS sites reported by others [[3](#_ENREF_3),[4](#_ENREF_4)]. STAT5 motifs/binding sites have been found close to the transcription start site [[7](#_ENREF_7),[8](#_ENREF_8)] or within introns [[9](#_ENREF_9)]. Approximately one fifth of our sequences were found to be close to genes on regulatory elements (including promoters). The remaining sequences were located within introns in accordance to previous reports [[9](#_ENREF_9)], as well as other intergenic regions and repetitive elements. Both methods isolated repetitive elements, leaving open the possibility that STAT5 might bind to such elements to control transcription from a distance, as shown to be the case for other transcription factors, i.e. C/EBPa [[10](#_ENREF_10)]. Furthermore, our observation of the binding of activated STAT5 protein to repetitive elements bolsters similar reports (i.e. for STAT1 [[11](#_ENREF_11)], p53 [[12](#_ENREF_12)]) and supports the notion of the involvement of repeats in the regulation of gene transcription.

Expression of *Dpf3* and some of the other STAT5 target genes identified was induced upon IL-3 stimulation, suggesting a potential involvement in STAT5 physiological function. ChIPs confirmed direct binding of STAT5a to the isolated sequences and/or the promoters of *Dpf3*, *Lrg1*, *Mta3*, *Rnf41* and *Traf1* genes, indicating that these genes are directly regulated by STAT5a.

Our findings in the various hematologic malignancies are in accordance with the deregulation of STAT5 target genes in cancer samples reported by others [[13](#_ENREF_13)]. Furthermore, we observed deregulation of *DPF3* in a solid malignancy (breast cancer) and association of higher *DPF3* mRNA levels with an advanced grade (data not shown).

Activated STAT5 in these malignancies might result in over-expression of the *DPF3* gene and modified DPF3 protein levels, leading to deregulated interaction with acetylated lysines of histones 3 and 4 and mono- and dimethylated lysine of histone 3, driving abnormal activation or repression of genes involved in neoplastic transformation. Taken into consideration that binding of DPF3 to chromatin can be regulated by different histone modifications during the initiation of gene transcription [[14](#_ENREF_14)], our data speculate that STAT5 is potentially implicated in this process.

The analysis of *DPF3* and the other STAT5 target genes identified in larger groups of hematologic (or other malignancy) patients highlights their potential use as biomarkers and will provide useful tools for diagnosis, prognosis and further classification of various forms of cancer, since particular expression profiles/signatures [[15](#_ENREF_15)] correspond to specific stages of neoplastic transformation.

In addition, the completion of the STAT5 target genes map will help characterize the mechanisms of STAT5 dependent transcriptional regulation and complement current diagnostic predictions of tumor behavior based on protein and mRNA analyses [[16](#_ENREF_16)].

**References S**

1. Horsman S, Moorhouse MJ, de Jager VC, van der Spek P, Grosveld F, et al. (2006) TF Target Mapper: a BLAST search tool for the identification of Transcription Factor target genes. BMC Bioinformatics 7: 120.

2. Carmona-Saez P, Chagoyen M, Tirado F, Carazo JM, Pascual-Montano A (2007) GENECODIS: a web-based tool for finding significant concurrent annotations in gene lists. Genome Biol 8: R3.

3. Soldaini E, John S, Moro S, Bollenbacher J, Schindler U, et al. (2000) DNA binding site selection of dimeric and tetrameric Stat5 proteins reveals a large repertoire of divergent tetrameric Stat5a binding sites. Mol Cell Biol 20: 389-401.

4. Ehret GB, Reichenbach P, Schindler U, Horvath CM, Fritz S, et al. (2001) DNA binding specificity of different STAT proteins. Comparison of in vitro specificity with natural target sites. J Biol Chem 276: 6675-6688.

5. de Boer E, Rodriguez P, Bonte E, Krijgsveld J, Katsantoni E, et al. (2003) Efficient biotinylation and single-step purification of tagged transcription factors in mammalian cells and transgenic mice. Proc Natl Acad Sci U S A 100: 7480-7485.

6. Grimley PM, Dong F, Rui H (1999) Stat5a and Stat5b: fraternal twins of signal transduction and transcriptional activation. Cytokine Growth Factor Rev 10: 131-157.

7. Rascle A, Lees E (2003) Chromatin acetylation and remodeling at the Cis promoter during STAT5-induced transcription. Nucleic Acids Res 31: 6882-6890.

8. Brockman JL, Schroeder MD, Schuler LA (2002) PRL activates the cyclin D1 promoter via the Jak2/Stat pathway. Mol Endocrinol 16: 774-784.

9. Nelson EA, Walker SR, Alvarez JV, Frank DA (2004) Isolation of unique STAT5 targets by chromatin immunoprecipitation-based gene identification. J Biol Chem 279: 54724-54730.

10. Liu X, Wu B, Szary J, Kofoed EM, Schaufele F (2007) Functional sequestration of transcription factor activity by repetitive DNA. J Biol Chem 282: 20868-20876.

11. Schmid CD, Bucher P (2010) MER41 repeat sequences contain inducible STAT1 binding sites. PLoS One 5: e11425.

12. Wang T, Zeng J, Lowe CB, Sellers RG, Salama SR, et al. (2007) Species-specific endogenous retroviruses shape the transcriptional network of the human tumor suppressor protein p53. Proc Natl Acad Sci U S A 104: 18613-18618.

13. Basham B, Sathe M, Grein J, McClanahan T, D'Andrea A, et al. (2008) In vivo identification of novel STAT5 target genes. Nucleic Acids Res 36: 3802-3818.

14. Zeng L, Zhang Q, Li S, Plotnikov AN, Walsh MJ, et al. (2010) Mechanism and regulation of acetylated histone binding by the tandem PHD finger of DPF3b. Nature 466: 258-262.

15. Golub TR, Slonim DK, Tamayo P, Huard C, Gaasenbeek M, et al. (1999) Molecular classification of cancer: class discovery and class prediction by gene expression monitoring. Science 286: 531-537.

16. LeBaron MJ, Xie J, Rui H (2005) Evaluation of genome-wide chromatin library of Stat5 binding sites in human breast cancer. Mol Cancer 4: 6.

**Table S1**

**Primers-oligos used in the study**

**Table S2**

**Selected potential STAT5 target genes**

Selected STAT5 target genes for further analysis are shown with their symbols, names, Ref Seq accession numbers, corresponding sequence code numbers, presence of ttCn_3/4_GAA motifs on the isolated sequences/promoters and distance of the isolated sequences from the gene. The distance is calculated between gene and sequence borders/coordinates in UCSC (NCBI36/mm8 mouse assembly).

**Supplemental Figure legends**

**Figure S1**

**Comparisons of efficiency of the methodologies used.**

Chromatin precipitated DNAs with antibodies or/and streptavidin beads were analyzed by Real time PCR to assess STAT5a binding on known STAT5 target genes and to allow comparisons of the efficiency of the methodologies used. Cross-linked chromatin from cells deprived of IL-3 for 6 h and then stimulated with IL-3 for 30 min was used. Ba/F3 cells were used for ChIPs and BirA/bioSTAT5a Ba/F3 cells for chromatin streptavidin precipitations (sp) and for ChIP followed by streptavidin precipitations. Primers specific for *Bcl-X*, *Cis* and *Socs3* target genes containing STAT5 motifs were used, as well as primers for negative (neg) control regions close to *Cis* and *Socs3* genes, lacking STAT5 motifs. Bars demonstrate mean and SD values of specific enrichments (fold differences) versus input obtained from at least three independent experiments. For samples in which no bar is visible, specific enrichments were very low. Anti-STAT5a (a-STAT5a) antibody and IgG control were used in all ChIP experiments.

**Figure S2**

**Expression levels of selected STAT5a target genes.**

Ba/F3 cells deprived of IL-3 for 6 h (starved) and stimulated with IL-3 for 30 min and 3 h were used. Expression levels (mRNA) were measured by real time PCR with the deprived of IL-3 cells set as 1. Bars demonstrate mean and SD values obtained at least in three independent experiments. The statistical significance of the difference in expression between 30 min stimulated and deprived of IL-3 cells is indicated with asterisks (ns: not significant, *****0.01≤P<0.05).

**Figure S3**

**STAT5 binding to the novel target genes.**

**A. STAT5a binding to** ***Lrg1*, *Mta3*, *Rnf41* and *Traf1*:** Cross-linked chromatin from Ba/F3 cells deprived of IL-3 for 6 h and stimulated with IL-3 for 30 min was used in ChIPs with anti-STAT5a antibody. Three sets of primers were used for each gene (one set specific for the amplification of the isolated genomic regions/cloned sequences (seq), found in proximity or inside the genes in question and containing at least one TTCN_3/4_GAA motif, one set specific for the promoter of each gene (pr), containing at least one TTCN_3_GAA motif and one set of negative control primers (neg), for amplification of a region lacking TTCN_3/4_GAA motifs). IgG was utilized in parallel with anti-STAT5a antibody, as control. Bars demonstrate mean and SD values of specific enrichments (fold differences) versus input obtained at least in three independent experiments. The statistical significance of enrichment versus the negative control region is indicated with asterisks (ns: not significant, *****0.01≤P<0.05, ******P=0.008).

**B. STAT5a and STAT5b binding to *Dpf3* in cells deprived of IL-3:** Cross-linked chromatin from Ba/F3 cells deprived of IL-3 for 6 h was used in ChIPs with anti-STAT5a and anti-STAT5b antibodies. Primers specific for the amplification of the cloned/isolated sequence, the promoter and the negative control region were used. IgG was utilized in parallel with anti-STAT5a or anti-STAT5b antibodies, as control. Bars demonstrate mean and SD values of specific enrichments (fold differences) versus input obtained at least in three independent experiments. The statistical significance of enrichment versus the negative control region is indicated with asterisk (ns: not significant, *****P=0.03).

**C. Sequence of the mouse *Dpf3* promoter:** The wider promoter region is shown with the STAT5 consensus motifs (TTCN_3_GAA) in bold and italics, and the non-consensus motif (TCCN_3_GAA) in bold. Oligonucleotides used for EMSA are underlined. Transcription start site is depicted with an arrow.

**D.** ***In vitro* STAT5 binding on motifs in the *Dpf3* promoter:** EMSA assays were performed with double stranded γ^32^P-labeled oligonucleotides containing one or two STAT5 motifs. The oligonucleotides were incubated with recombinant STAT5 protein in the absence or presence of unlabeled competitor oligonucleotides in 5x and 10x molar excess. The positions of the specifically retarded complexes are indicated by arrows, and an asterisk indicates the supershifted complex. Oligonucleotides with mutated STAT5 motifs were used as negative controls in all experiments.

**Figure S4**

**Efficiency of STAT5a and STAT5b knock-downs.**

After transduction with viral supernatants corresponding to different shRNA clones, the cells were maintained in puromycin selection for 10 days, subsequently deprived of IL-3 for 6 h and then stimulated with IL-3 for 30 min. Expression levels (relative mRNA levels) in the deprived of IL-3 cell state and 30 min after stimulation were calculated with Real time PCR and compared with Ba/F3 cells transduced with scrambled shRNA, set as 1. Bars demonstrate mean and SD values of expression obtained at least in three independent experiments. Equal loading of cell extracts in immunoblots was verified by using β-actin antibody (lower panels), (Scr: Scrambled, a&b: double STAT5a and STAT5b knock-down).

**A.** *Stat5a* expression of Ba/F3 cells transduced with four different shRNA clones for targeting STAT5a (A0, A1, A2, A9) and **B.** immunoblot of the respective total cell extracts.

**C.** *Stat5b* expression of Ba/F3 cells transduced with four different shRNA clones for targeting STAT5b (B3, B4, B6, B7) and **D.** immunoblot of the respective total cell extracts.

**E.** *Stat5a* and *Stat5b* expression of double knock-down cells and **F.** immunoblot of the respective total cell extracts (in A-F the cells were deprived of IL-3 for 6 h and then stimulated with IL-3 for 30 min).

**G.** *Stat5a* expression of Ba/F3 cells transduced with the A0 shRNA clone for targeting STAT5a and **H.** immunoblot of the respective total cell extracts.

**I.** *Stat5b* expression of Ba/F3 cells transduced with the B3 shRNA clone for targeting STAT5b and **J.** immunoblot of the respective total cell extracts.

**K.** *Stat5a* and *Stat5b* expression of double knock-down cells and **L.** immunoblot of the respective total cell extracts (in G-L the cells were deprived of IL-3 for 6 h).

**Figure S5**

**Immunofluorescence detection of activated STAT5 (p-STAT5) and DPF3 in CLL.**

**A.** Selected field of PBMCs cytospin of a CLL patient immunostained for p-STAT5 (green) is shown. Granulocytes are pointed by arrows (examples of nuclear p-STAT5 staining are depicted with solid arrows and cytoplasmic staining with dashed arrow). Negative control immunostaining with goat IgG is shown on the right (Scale bars: 10 μm).

**B.** Examples of granulocytes with nuclear & cytoplasmic p-STAT5, cytoplasmic p-STAT5, nuclear DPF3, nuclear p-STAT5 & DPF3 staining are depicted from the left to the right. p-STAT5 staining is shown in green, DPF3 staining in red and double p-STAT5 and DPF3 staining in red and green, or yellow (Scale bars: 5 μm).

In all images DAPI staining is depicted in blue. The original magnification used was 40X. Images were acquired with a Leica TCS SP5 Confocal Microscope with dual (Tandem) Scanner.

**Figure S6**

**Sequence of the human *DPF3* promoter.**

The wider promoter region is shown with the STAT5 consensus motifs in bold. The transcription start site is depicted with an arrow.

**Supplemental Figures**

**Figure S1**

**Figure S2**

**Figure S3**

**Figure S4**

**Figure S5**

**Figure S6**

**Sequences S1**

Sequences analyzed from the ChIP followed by streptavidin precipitation library. Vector sequences are deleted.

>Sequence_1

GAGAGGTTAGAAGCAAGAATCACACAGTGTAGATGCACTTACTTAATCTACATGGATCACGAATGCAGGCAGCAAAAGCTTAGAGGGAAAGAGAGACCTGGTGCAGGGGATTCGCCTGCTGTTTCCTCCAAGAATATGGGACTGGAATGAGGGGCCCCTGGAGACATGTCTTACAGCTGTCACACATTCAAGCTACAGCAGGCACTCTGGGAGCTCAGCTGAGGGGGACTTTATTTGAACTGATGATTTATGATGATTCCAGACTAGGGTTATACTAAGTCTCACCTAGAAAGTTATGTTGAAACTCTAGCTCCTGGTTCCTGTGTCTTTATTTGGAAGTAGGGTCCTTGCTGATAT

>Sequence_2

TGACTATCATCCCACCCACCAAACTGCAGCATGCTCATCCGGCACAGCATGGCCTGTCTTCATTCAGTTACCCAAGGCCCCACTTGCTAACTGAGATGGCTTCAGTAAT

>Sequence_3

AGGTCTATGATGTAAGACCAAAAGTGCACTGCCCTTAAAGGAAAGGACAGATGGCCAGGATTACAGAAAGAAAAGAATAAGAATTCACACACATGCACACTACAAAGATGGCGGAGGAATAGTAGACAGCGCTGCTCAGTGTTTCAAACTGGCAAGGTATCCCAACACAGTAGCACAGGCTGTAAAGTCTCAGCAGTCAGAGGCTAAAGCAAGAGAATCCCTTGAGCCCGGGCATTC

>Sequence_4

ATAATAANTAANTAATAAAAACATTAAAAACACACACATACACACAACCAGAAAAACAAAACAAAAACAAAAACAAATGTGA

>Sequence_21

TGAAGACGTGTTTTAAATTGCATGGGTCCCATGCAATATTGCAAGTATCGAGATACACTTCTGCTGAAACAAACAAACAAACAAACAAAAAAAAAAAAAAACATTGACTGTCCAAAATTCTAAATTAATCAGGAGTCTTAACTATCCCCCCAGCCTATCCCCCCAGAAAATCCTAATCCTGGCTCTGACCTTTGTAATATTGCTTTATTGCAGGAATTCACAAGCCATCTGTAAAGGGCCACTGTGTGGGGCTCAGTGACAGTGTTTGTCTAGCATGCACAATGCTTTGAGTTCAATCCCTAGAACCAAATAAAAGGACCCGTTCTAAAGGAAAAATAGGGAAGTCTATGTAGTTGGATTAGTGTGCAAATCATTGAGGGTTTCTTTCTTGTGGATGACTGGAGTCAGCCCTCACATCATGTTAGCACTGAATG

>Sequence_22

GCTCAGCCCTGTGGAGTCTTGTTCCCAGATGTGCAAATGATTGGCA

>Sequence_23

ACCAAAAAGCCCAGTAGGCAGCTGGATACAGTGTTGCATAGTGTAACCCGTTCAGTCCTGGGAAGCTGGACAAGACACAGGGCAA

>Sequence_24

GCATCACCCGGCTCTGCGACCAGCTGAACACCCAGGGAATGGCGCGCAGGTCTTC

>Sequence_36

CTGGATCAGGCTGCTGGTTTGGTGTCCTTTCCCGTGAAGCTGACTCAACCTGTTATTGATGCCGTGCGGGTTCGTGCTGCCGAGCAAGGCGTATCGACCCATGAAGTGATTGACCGCCTGCTGGCCCAAGCCCTGGACCTGTAATCCATGCAGATTTGGGTCGATGCCGATGCCTGTCCGGTGGTTATCAAAGAAATTCTTTACCGTGCCGGGCAACGTTGGAGCCGTCACGT

>Sequence_37

TAAAACAAACTGTCTCATATTCCTTTTGAGAAGACAGCTATGTTTTGCACCTACATTTATGTTGTACAATACCCCAATTTCATTCACTCCTCTAGCACACATACAGAAAGAAAAATAAGAAAGACTTTCAATGACAGGCACGGCATCACATACCTGTAATCCCAACTGAGGAGACAAAAGTGTTTTTAAGTGATTTTAAGTCCAGACTGTGCTGTGGAGTGAGACCCAGTTTCAAAAATCAAAGTGAAAGGATACCTCAAAATGCATCCATTTGTAACTGCAAATGGTCTTCTCATCATTTGTCAGAGACACACTTGATGTGAAAGACATAGAATCTTATAACATGAAGAAAAGACATACTTCTTAAGAAGTTTAAAATGTAATTACAAAACAAACTCTATTTAATTGTCTAATAAATAAAATACACAATAAATAAATGTTTGTAAAGGTTCAAAACGCTCCAGAAGATGCAATGTTTTGGCTTTTTAGTAGCTGTGAATTTGGGCCCTTATCTCCAACATAGGGTCTAGCTCAGATATAAGGTGTTTGAAAAATGTGATGCAATTTAAGTACATCATGTAATGAGTGCTCAGATGACTTATTGCTTTATTATTTCTCTGGCTACATTGNTTCTATGGAGATC

>Sequence_38

GATCCCTCTAGCTCCTTCGGTTCTTTCTCTAGCTCCTCCATTGGGAGCCCTGT

>Sequence_39

CACGACCAATGGACCTCTCTTTCACTGATGGCCGACTAGGCCATCTTCTGATACATATGCAGCTAGAGACACGAGCTCTGGGAGGGTGTACTTGGTAGTTCATATTGTTGTATTGTTGTTCCACCTATAGGGTTGCAGATCCCTTTAGCTCCTTAGGTACTTTCTCTAGCTCCGCCATTGGGGGCCCCGT

>Sequence_40

CAGTCACTAATGTGTGGGCTTTGCTAAAAACAGCCCCGATTTTGCTTGAGTTGTAAACTGAGCAAATGAAACTGGAGGAGACATCGGGAACAGATGTTGATGTTGAAAGGCCACTTATCTATCATCACATTTTTATATCATCAGCTGAAACTAGCATTCAATCCCCTTTCTTATTTGATTGAAGTGAACCTATAAATCCGAGTCTTAAAATTCCTAGTAAAAATGGAATAATTTATTTTGAAAACTTTAACTATTCTGTAGCCCTAACGTCCCCAAATTCATCATACATAAAT

>Sequence_41

GATCTGAGCTTTAGCTCAGGTCCAGAGGCTTTTTACCTGCTAACCCTACGATCAGTAGCACTAAGGACAATGTAGAAGGAAAGATTAGCAACAAGAAAACTTGGCATGAGAAAAGTACCTGTGCTACCTGTCTTTATCTATTGTCAAAGTAGGTCGGAGGAAGTGTGAGTCTGAAATAGAGTCTCAGATTCTAAGAAGAAAAGAGGCAGCTTTGTTGTTTTATCTTAACCCTGTAGGGTGTGGCTTTGGTTTGAGGCTGGGAGCCAAGCAGGAGGAGTTCTTGAAACAAGTGGTAGTGACTTCTCAGCAGAGGTGACTTGATGCTTCTGTACAGTGGACTTTTATAGGCGGGATTTCTTCCTTCAGATGCTGTTGTGAGCCTGCCTTTGCAAATGCCTTTGGTTCCAGTCTCTGTGCAGATTTATTATAAGGTCAATAGCTCCCACGTGTGTACCTGTTATGGTTTAAGGGGCTGTGCCTCTCATGGCCTTGCAGGGATATAGGTCTTTGCAGACTCTGCCTGAGTCATCTTGACCTTTTGTAAAATCAAGGTTTCATCTTAAGGATGATTTTTAAGGGATGAAATGTGAAAGTAGTTCTACATGCTGAGACATGCCTTTTAATTAC

>Sequence_42

GATCCCTTTAGCTCCTTGGGTACTTTCTCTAGCTCCTCCATTGGGAGCCCTGT

>Sequence_190

GATCACCCGCTCGCCGGGCTTCAGCCCGAGCTTGAGGAAACCGGCCGCCGCCGTCTCGGCGCGCGCATGCAACTCCCCATAACTCCAGCGCT

>Sequence_191

GATCTGAAGTCTTTCTGCCCTAGTCTAAGGACTGGCTCTCTGGGCATGAGCTATGACACATGACCCTAGCTTTTTCAGGAAACACAAATCAGAGAATCATTGTGAAGGTTATTATGAGTAGGCAGCCAGCAAAACCATGCCTTTGAGAGCCTCCCTTTCTCTCTCTTGCCCTCTTT

>Sequence_192

GATCTGAAGGTGAGCAAGTCAGTCCTGTGGAATGTGTCCCAATGTGGTCAGGTCGGTTCCCACCCTGT

>Sequence_193

GATCAAGCCCGGGTAGCTTGCTTCCCTATGTACCGCAGTCTCAAGTTCCACGCGATTGGATTGGGGTAGGCGCTGTGTTCCACTCACCAGAGGTCTTAGGGTCCCGTGGGGAGTCCCGTGTGGGCCCTTGCGGGTGTTGGGCAAGACTCTGCTGTCAAGGTAGCCCGGGGCTCGAGTCTCGAGTCGAGCGGAAGGGACTTGTGCCCCA

>Sequence_194

GATCCTCCGGTAGTACTATGTCCAATTTTCTGAGGAACCGCCAGACTGATTTCCAGAGTGGTTGTACAAGCCTGCAATTCCACCAACAATGGAGGAGTGTTCCTCTTTCTCCACATCCTCGCCAGCATCTGCTGTCACCTGAATTTTT

>Sequence_195

GATCTTCTAAAACAGTCAGGCATCTGTTTTACTTCGGTTCCCTTTTAGAAATCAGGATATTGGGAACCCTGGTGCTTAGAGTATAGCAGTGTAAAAATACATGGTGTATCAAAAATATTGCTTGAGGGGGCTGGAGAGACAGCTTAGTGGTTAAGAACTCTCTTGCTGCTCTTCCCTAAGACTGGAGTTTGGGTTCCAGCACCCACATCAGGGGGCTCACAACTGCCAGTAACTTGGGTTTCAGGCACNCTGATGTCCCTCCCATGTGCATGTACACACACATGTAAAGACACATGCACACACACACATAC

>Sequence_196

GATCGTTCTTTTCAGTGTTCCTATTTTTTCTAGTGACTATATTCTAGTACCATTTACAACACTTTTATTCCTATTTTGAATGGGATTGTTAGAAGTACTTGTTTTGGGTTTGGGAGTTTGCTCAGTAGTTAAGAGCACTTGCTGCTCTTTCAGAGTACCTT

>Sequence_197

GATCCCTTTTCTGCTTCTCAGCTTCCAGGGAGCCGGATGGTTCTGAGATGCTACCAGGCTGGGTTCCCCAGTCCTCACAGGGTCCTTCTGATGAACAAGAACTCAGAGCTCCAATCCTGAGAGCCCTTCCTACCTGTTTCCCACCAGGAAAGACTTCCTGGTTCCAGAAACCTATGGCGTGAAACACATGGCCTTGGCCACTGCTATTTCTCAGAGTGCCTAGTTATTATTGGGACCTGAGGTTGAGCAAGGCATACCAGTTTCTTCCTGGCTGGCAGGCAGCAGCTCTGGTTCAATATCCCCCCTAACTGACTGCGCCAAACTCTACCCTGCTACTTTAACCCTCAGTCTTCAAGGCAGCAGTAGGCAAGCCTCAGGCTCCCGTGTTCCACCACCTGCATGGCTTCAGTAGGCCCGGGTGCCTTGCTCCCCCGGGGAACCATTTGTCCCTTCTACCAGCTCTGCCCCCTCACTCTCCCTTAGGGGAACCTGGGAGCAGCA

>Sequence_198

GATCCCCTTGAATTGGAGCTACTAGTAATTGTGTGGTGCTATGCAAGTGCCATGTGGGTGCACTCCAGTAAACTTGTACTCTTCTGCCAAGGAGTAAGTGCTCTTAAATACAGAGCTCATCTCTCCAGTACCAGCTGTTGGTTTTTAAATGACTACTTCCTATACTGAAAGGAATATTTTATAATTTAAAGTACTCAGGCAAAATATCTTCAGTAATTGTTCTCAAATATC

>Sequence_199

TGGTCAATATGCTGAAGGCTGTTCTTG

>Sequence_200

TGGTCAATAGGCTGAAGGCTGTTCTTGATTGTCACA

>Sequence_201

GATCCTTGGGTACTTTCTCTAGCTCCTTCATTAGGGACCCTGTGTTTAAATATGCATTGGTGAGCCAGTCCCTTCACTGGTTGTCTTGTCTATCTACTGAAGGTGGTCTCTCTAAGTTCTATCTCCTCACACTT

GGACATATCATCTATGGACATCCCTGGAACTGAATCCAGGAGGCCTCTCACATCCCATGTCTCTGGGACTTTCTAGAGGA

CCCCATCCTCCATGCCCAATCCCACTCTGCTGCATATTTCTACTCATTCTCCTGGCCCTTGAGGCCTCTATCCTCTCTCC

CCATACCT

>Sequence_202

GATCCTCCTGTAGTACTATGTCCAATTTTCTGAGGAACCGCCAGACTGATTTCTAGAG

TGGTTGTACAAGCTTGCAACCCCACCAACAATGGAGGAGCTTTCCTCTTTCTCCACATCCTCACCAGCATCTGCTGTCAC

CTGAATTTTT

>Sequence_203

GATCTTTCCATCTTCTGAGATCTTCTTTAATTTCTTTCTTCAGAGACATGAAGTTCTTG

TCATACA

>Sequence_204

GATCACAGCCCTTTGTGAAGGGATGTCAAAACAATTCAAGTAAGAACCTGAGT

CAGAAATCATGAAGGAAGGTTGCTGGGTGACTGGCTGGCTTACACAGGCTCATACTCAGCTAGCTTTCTTATACAGGTCA

GGACCACCTGCCTAGGGAATGATGCCGCCCAGAGTGGGCTGT

>Sequence_205

CTTCTGATTGCAGATACAGTGTAACTGGGGCA

TCAGACTCCTGCCACGATAGCTTCTGTGTCATGTTGGACTGTACGCTCAAAACTGTGAGACAAACAAACAACAAAGAAAA

CAAAACAAAACAAACAAAACCCAAAAAACCCAAACCCAAACCCCTTTCTTTCCTTTACACTGCCTTTTTAAATAACAATG

AGAAAAGTAACTAATGAATCTTCAGAACAATTTAGGCCCGAGATTCATATCAAATCTAGGAGATTTGTTAGCACTCTGTG

CCGAGGTCTGCATTTCCACAGGCCTTGGCGTTCCCATTTCTCTAGAACACTCTGAAGCGTGGTCTGTGGACATGGACTTG

CCTTCCCCTGGGTGTGGGCTACAACTGCAGCATCTCAGCTTGAGTCCCAGAAGTGCTCTCCTCCGCTGCTTTAACTCAAA

CCCCCATGGAGAGATCATCCAAGTTGTAGGTAATATGCAGACTTTGAGCAGGGTACTCTGAAAGTCATTGTTTCATCTGA

TTCCAGGAATAGACATCCTTGAC

>Sequence_206

GGATCGCGACACGCTTTAGAGCCGTTGCTTGTTTCACGGAATCG

CTGAACGGCTCCAAGCCTTTGGTTTGTCGCGTTTCCGAACCGGAAAAGTGTTTCCACTTTACCTGGAAACGCTCTGGC

>Sequence_207

GATCCTTCATCCATCTTGCAAAACGAGCGATCCAG

ATATGCTCAAACCCACCCCATCCCACCCCACCACGCAGAATGCCCTTCGGATGTTAGTAGGTCGTAAGCAAAGACAGCAG

TTGCTCCTGGGTGGAAACCAGCTTTGGCAGTATTTTCTCAATCAGCCGACCTTCCTCCGTCCAGTCCACTGACTGTTAGG

TCCAAAGATTACTTGCTGCCCTGAGTAGGTTTGAAACAAAAAGCCACGCCCGCGCGGTGCGTTCCTCGGGCCTACAGCGC

CATCTTGTGGCGCTATGGCGTTAACACACTGGCTTTTGCATCCTCAGAAACATCCAGGCATTCCCAA

>Sequence_208

GATCAAAAGGCAAAGTTGTGGAGTCCAAGCCCAATGGTGCAT

CAGTAAAACAATCCCCACACCTAAGGCTCAGGAGTCATTATGGAAGAAGAGGAGGAAAGACTATAAAAGTCAGAGGACCA

GGGAGATGCCATGAAACCATGTGTCCTAGTAGTGTCAGAAGCTGTACTCCCACCCACCCACCACCAAAAAAAAAAAAAAC

CCTCACAGATGAGCCTACCTAAACATGAGCT

>Sequence_209

AAATGATAAAAATGTATCAGCTAAGGCCAAATGGAAG

GTTTTAGTTTTTCCTTTTAAAAAATTGGTTAATTTATTTATTTACATTTAAAATGTTATCACCTTTCCTAGTTTCCCCAC

TGGAAACCACCCATCCCATCCTCCTGTCCACTGTTTCTATGAGGGTGCTGCCATATCGATCCACTGCAAGAGCACGCCAC

ACTCTCAGCCACTAAACCATCTCTCAGCACATAGTCCATGTTCTGTTCTGTTGCTGTTTGACACAGGAAACCCTGGCTAG

CCTACAGCCATAACCCTGGTTAGCCTACAGCCATAGCCCTGGTTAGCCTACAGCCATAGCCCTGGTTAGCCTACAGCCAT

AGCCCTGGTTAGCCTACAGCCATAGCCCTGGCTAGCCTACAGCCATAGCCCTGGCTAGCCTACAGCCATAGCCCTGGCTA

GCCTACAGCCATAGCCCTGGCTAGCCTACAGCCATAGCCCTGGCTAGCCTACAGCCATAGCCCTGGCTAGCCTACAGCCA

TAGCCCTGGTTAGCCTACAGCCATAGCCCTGGTTAGCCTACAGCCATAGCCCTGGTTAGCCTACAACTTATTG

>Sequence_210

GACGACATGCGCCGGATGGCGCGTGAGCAGGAGTTGACA

GAACGGCCATCCGCTGGGGATGGCAACTTCATTTCGGTCGCCGTGGTGCGCGCGTTCCGGAGCCTCGCTAAGGGCGGCTA

GTAACACCTTTGAAATGCCCACGCGCGGGAACTGGTTTCGACGCGTGGGCTACGCCGCTGAGGGCTTCGCCCAACGACTC

CTAAAGAATCCGCCCACTCGGCGCCCTTACCTCGCCCGCCAGCTTCTGACGCTTCATCGACACTGCGGCATCGACCTCGA

AAGGAGATGCCATGCTCTGGCTCATCAGAAAACTGCTTAAGAAACGTAAGCGCAAGCCGGTCGGCAATACCGAGCCCAAT

ACAGCGCCAGCGCCCCTCGCCCGCAAGGCAAAACCCGAATGGGAAACAACTCTTCTGGGCTCGTTCGAACAGAACAATGT

GGCACCGACGGACGAAGTCATCCTCCAGTCCTTCGATGAATTCCAGAACGACCTCTACCGGCAGATTACCGAGCTTCGCC

GGGTTCTGGCGAATGGCTATGCCGAAATCGTCGCCCTCGTCGCAGCGCTTCGCGCGATCATTCCCGG

>Sequence_211

GATCGTGTGAGCCTGCGCCACGGGGTCGCCGTTGCCTGAGCCGCGCTGCGACC

ATCCATGCCCCGGCCTGTCCAGAAACAGCATCTCTGCGCGACCTTCGAGCAATGGGCGCACCGGGACCATCTGGTCGAGC

AAATTGCCGCTCGCGCCGTGTATGAACACGAGCGGCGGCA

>Sequence_212

TCCGCTACCTCGGCCATTTCGGGCTTTTGCCC

GACCATTGGCAGAACATTCCACAAAGGCGTGCGTAAGTGCTCTCGATCAACAATCTCCATGTCTCCGTCGGTGAAAAGCC

>Sequence_213

GATCTGATTACCCTCCTTTTCAAACAATGTTTCGAAGCATTGATTGTAATAGC

CACCTGGCAGGTATTCAGCAGTCAATACATGTCTGACCAACATCTCTCCATCCCTCTGCACCAACTCTGCATTTCTGAGT

CTGCAGGCGTCTCAAAGAACACACAACAGAGTTCCTTCCACAGGCACATCGCAGCCTGTTAACAGGTGAAAATGCCTCTT

AGAACTTCACAGTCACCTAGAATCTCGGAATGTAACATCACTTGGAAAGAGAATCTTAGCCAGCATCATTCAGCTAAGGA

TAGAGATGAGCTAACCCTGAGTTAGGGTGGGCCCCACACCCATTAGCATCATCCTTACAAGAGACAAGAACACAGAGAGA

CAAGTAGGAAAACAGTGAAAAGACAAACAGAATCAGAGCTGCACACAACGGAACAACAGTGCCATGGGGTTCCTGGAAAC

TGGAAGAGACTGCTCAAGANGGAGCATGGCCTTGCAGAAACTCAGTCTGACTTAGAGCCTTCATATTTGTGAGAGAATCC

ATTTTTGTTCTTTTAGCTACTA

>Sequence_216

GATCAGCCTGCTTCAGGCTGGGGGGGGAGGGTTACACTTTCCCCCCTGTTATTGGCAT

AAAATTTATTTATGCTCTATAGTCCTACCTGGGAGTTATGGTGGCTGGCGGCGGCGCCTGACTTAGGAAATCTTA

>Sequence_217

GATCAGTATACAGAACAGAAGTCTGAGGGCCTGCACGACCCAGGAGGACAGCA

CGTCACTGGGAACCCTGACTGGCACACTGAAGGACATAAATCTTGAAGCTTCTTCTCATAGACTGGGGACTGGGGACCAA

CTCTCAATCCTAACTGCCTGTTGCCCATGCTCCCCACACAGTGCCCACACTCATCTATACCTGAACAGATAACAGAGCTG

CTGGCCCACACTTGGCCACTGGGAGCAAAGGGACACAGAGTGTCTTCCGGAGGTGATAGC

>Sequence_218

GATCCTGCTGCTTCCGCTTGGACTAGCTGAGATTACAAGTGTATGCC

AGTAGGTGTGGCTTCGTATTTTGAGGCAGGGTTTCACCGTGTAGCCTAGGCTGGCCTGGAATCCTCTGTGTAGACCAAGC

TGATGCTGAGCACACAAGAGTTGCCCTGCCTACCAAGTGCTGAGGATAAGGTATACCTAGCACAATGGTGTGTCTCAACG

CAGGGGCATCTTAACTTTGATGGGTATCTAGTGTCTACCGTAACAACCTTCTTTGCAGTTCACCTTAGTCATTCAGAACT

TTTGGTTGCAAGTCGAGCAACTTCCCCTCAAAACAAGAAAAAATTTGGCCCAGTAGTTGGACAGAATGCTGGGCACAGAT

CACTGGGTTAGAGGAAGACCTGCAG

>Sequence_219

GATCTAAAGGTGACAAAGGCATACAAAGGAAGCAGGACAGAGGGCCTACACCAGAAC

AGACAAGTTGGAGCCACAGGTGAACCCGAATTTAAGTGTCTCTTTCTGGCTTCACCAAACCTACCCTCCATCTGTCTCCA

CCCTCTTCCTTGTCCAGGGGAGCCAACCTATAGGACTTTATTATGTATCCCTTGGCCTTCTGCCCTCTAGTCAGGTTTAC

CCAAGGGGAGGCACTGGCTGGTGATGGAGGAGGAGGTATAGGAAAATGTTTATTCCCAGGCTCCATCCTTCCAGTACATT

TGGAAGGTACTGTCTCCCCTTTCCCCTGCCACCACCACTGCCCATAATCTCAGCAACTCCAGACAGCTGTTCTATATAAC

TTTCTCTTGAAGGTCTGGTGCCCTTTAAGCATATGGTGGTTAGAGCTCCTCACTGTTGCTGGCTTGAAGTACTTCACCAT

TCTCCCAGAGTTCCCTTCCTGCTACACCCACCTTGGTTAAAACAAAACAAGTTCAGACAGCAAAAATGCACACTGACAAC

CTTTATCAAGGTACCATCTCCTTTCCAAGTTTATCCTGACTGGTGCACATGCCACGATTTCTATAATATGCCTTTAAAAT

GCAGGTAGAGTGCTACAC

>Sequence_248

GATCCTCCCCACAGTTACCTGGCAACAGTAAG

GTAGCCCAGCCCACTATAAAAGGAGCTGCTTGCCCCCTCCTCTCTCTCTTGCTCTCTTACCTCTTATTCTCTTGCTCCCT

TGTTCCCCCTCTCTCCGCCTCTTCCCCTCCCCCTTTCCACTTGGCCCTGGTCGTCCTCTCCTTCTCTACACTTCTACTTC

TCTCTGACTTTCTACAATAAATGCCTTAAAACCACGAACCATTTCTTATTGAATCCACCATGCTGGAGC

>Sequence_249

GATCAGCATCCTGGTTTTCAGGTGCGTTGGTGTATCCAA

GGCTTACTGTGGCAGGCTTACTGGATTCTGATGATGCCATAGTATATTGACTTCTCTTGCTTAAGGTCTCATGCTTGCCT

CTTGCCATCTGGTTATCCCTAGTGTTAGCTGGTCTGGGTGTCTCTGTCTGGAGCCAGCCTCCTATGTTCCTGGATTGCTG

CAGGCATCCTGAGATGCTTATGACCCTGGCTGTAGCAGACCTTCTGGGGGGTCTTCAGACTGTGTGGTCTTCAGAGGAGC

AGTCAAGCTGAT

>Sequence_251

GATCAATTCGAATTCTTCTACATGATAACCGCCAGTTGT

GCCAGCACCATTTGTTGAAAATGCTGTCTTTTTGAAAGGATGGACCATGTAGAGACTGCCTTATCCAGGGATCCACCCCA

TAATCAGCATCCAAACACTGACACCATTGCATACACTAGCAAGATTTTATCGAAAGGACCCAGATGTAGCTGTCTCTTGT

GAGACTATGCCGGGGCCTAGCAAACACAGAAGTGGATGCCCACAGTCAGCTAATGGATGGATTACAGGGCTCCCAATGGA

GGAGCTAGAGAAAGTACCCAAGGAGCTAAAG

>Sequence_252

GATCTTGATTTAAAATTGGTACCTGGTATCTGTC

TAGAAACTTGCCCATTTCATCCAGATTCTCTAGTTTTGTTCAGTATAGCCATTTGTAGAA

>Sequence_253

TCACGCGTGGAGGCGCCGTCGAACCCCT

TTTCGGAAAACACTTNCAGCGCCGCCTGAAAGCAGGCTGGGCCTGCGTCGCCTCGCGTTTGCTGGACCCTGAGGTTGACC

TTGGGTCAAAA

>Sequence_254

GATCAAGCTTTAACCGGCAACAGCAGTAA

TGACTTCTGTTCTTTGAGGTGGAGGTTGCTGGTCCTACTGGGGTGGGGGTGGGAGCTCACTTGTGGCTCCTATGCAGCAG

>Sequence_255

GATCCTGAAGTGTACCTCCCTGCATCCACTC

ATTGTCAAACAAGCTAGTTTAATCAGACTTCTCAGATAGCTGCCAATTTAACATTTTTTTAAATCAGAGGTAAGCTATTT

AAAATCAACCAGAAAATTATTGACTCAAGCAATCCCTATACCTACATGACATGTCCAAAGCATATCTTTGCCATCTTTAG

AGCACTGATATTTTGAATAACCTTGCAGGAAATGACATAGTATGTCTTGTAAAGAGAAGAGAAACCTCTCAACTGCATAG

GCTTCCTCCATTCTGTGCAATGCCTTATCAAATCCAATATATGAATAGTAAGTGAGTTAAATATTTATTTAATTTGGTCT

TTTAATGGTCATTTAAATTGTGTCATTCCTACCAGTCACAAGAATATCCCCAAATCCTAAGGTTATTTCACTGCTTCATA

AGGTCTGTGATTTTTCACCCAAAATACAACGTATGTCAAGCAGAATGAATGTATTATTAACATAAGGCCAGCCAACTTTC

TATTCTACAGTGACCCCCAGCTTCAAGAAGCTGGTTCCACTTTCCAGGTCCTCTTTTACAAAACTGTCCACTTTCATGTA

CCACATGGTGTAAGTTAGTCATATGACTGTCCNCAGATAACAAAGACCCTGCAGTGTTCTAGTAATGGATATTCTTACGA

GTTTCTGCATAACATGC

>Sequence_256

GATCCATCCAATAGCTGACTGTGAGCATCCAC

TTCTGTGTTTGCTAGGCCCCGGCATAGTCTCACAAGAGACAGCCATATCTGAGTCCTTTCAGCAAAATCTTGCAGCTAGC

CAGCCAGCCCTCA

>Sequence_258

GATCCCAACCCTCCTAACACTGAGACA

TTTAACACGGTTCCTCGTGTTGTGCTAAACCCTTCATCATAAAATCACTTTGTTGCTACTTTATAACTGTGATTTTGCTA

CTGTTATAAATCATCCTGTAAATATCTGAGTTTTCTGA

>Sequence_259

GATCAAAAACTTTTGCATGAAAAACATACTCT

GATATGTTTCTGTTTCCCAAATATTTGGAGAGACCAAGAAAGCATTACAGGCATCTGTAAGAGAAGCCCAAGCAGCCTAA

GGTTTAAGCCCTGTCATCGGCTCCTCCGTCGGCTGCTTGCTGACAGCAGGTTACAACAAGGTTAATCATTCATTTGATGA

TA

>Sequence_260

AGTCACCATTTCGTATATTTGTTTCTAA

GCCCCAACATCTATGTGTATCCACTACATAAACCCAGGCATGGTGGCACAGCCCCTGAGAGGTGAAGGCAGGAATTCAAG

GGCAACCTTTATTGCAATAGGGAGTTCAAGG

>Sequence_262

GATCCTCTGGTAGTACCATGTTCAATTTTCT

GAGAAACCACCAGACTGATTTCCAGAGGGATTGTACAAGCTTGCAATCCCACCAACAGTGGAGGAGTGTTTCTCTTTCTC

CACATCCTCACCAGCATTAGCTGTCACCTGACTTTTT

>Sequence_263

GATCCTCTGGTAGTACCATGTTCAATTTTC

TGAGAAACCACCAGACTGATTTCCAGAGGGATTGTACAAGCTTGCAATCCCACCAACAGTGGAGGAGTGTTTCTCTTTCT

CCACATCCTCACCAGCATTAGCTGTCACCTGACTTTTT

>Sequence_264

GATCTCTTCTTCCACTATGAGTCCCAAGGCTC

AAACTCAGAGCACCAGGCTTGGAAGCAAGTACTGTTATCAACTGAGTCATCTTACCAAGCAGAAGTAAAATTTAAATATG

TGCAGTACTAGTGTTCATAGTCAAATGTTCAGACTATAACAAAGGTATAAAATAAAAGGACTAATAGAAGAAGGGGAGAA

GAGAGGGAGGG

>Sequence_265

GATCTCTTCTTCCACTATGAGTCCCAAG

GCTCAAACTCAGAGCACCAGGCTTGGAAGCAAGTACTGTTATCAACTGAGTCATCTTACCAAGCAGAAGTAAAATTTAAA

TATGTGCAGTACTAGTGTTCATAGTCAAATGTTCAGACTATAACAAAGGTATAAAATAAAAGGACTAATAGAAGAAGGGG

AGAAGAGAGGGAGGGAGAGG

>Sequence_266

GATCCACCTGTTAAATCTTAAACCCTGGGACAAATGGCGGAGGTTCCTGTTTA

ACATACCAAAGTGGACCTGGCCTCCAGAATCTCTTAGTACCCCTCAGTTCTTACTTGTTACAGGGCATGGCTGGCACACC

AGTCCTCTACCCTCAACTCCCTAGCCCCAGGACTGAGCTGCCCTTCCCTATATAATCCAGCCATTTTGGTTACCTGCCCT

CTTCT

>Sequence_267

GATCCTCCCCACAGTTACCTGGCAACAGTAA

GGTAGCCCAGCCCACTATAAAAGGAGCTGCTTGCCCCCTCCTCTCTCTCTTGCTCTCTTACCTCTTATTCTCTTGCTCCC

TTGTTCCCCCTCTCTCCGCCTCTTCCCCTCCCCCTTTCCACTTGGCCCTGGTCGTCCTCTCCTTCTCTACACTTCTACTT

CTCTCTGACTTTCTACAATAAATGCCTTAAAACCACGAACCATTTCTTATTGAATCCACCATGCTGGAGC

>Sequence_401

GATCCTCAACTTGATTTTGTTTCTTTACCTCCAAGTGC

TGGGATTATGGGGCCACACCACCATACCAAGCTATTAAGCTTCTTTTGTATTTTGTGCCTTCTAAGGGTAGAGGATTGCG

CATTGTTCACTCTTTATATCACCATGCTCCAGAGGGCCTGATATATAGGCAATCAGAGCACTTATTCTGAATTATGTTAA

ACCTACAGAACCCACCATAAGAGTATACTTCACAAACCTTCTTTTTTTAAAAAAAAAATTACTGTTTTCTAATTTTATGT

GCATTGGTGTTTTGCCCTGACATATGTCTGTGTCAGACTGTG

>Sequence_402

GATCAAGGTGGAGTGGAAACCCCGGATTGGGGGCTGG

AGAGATGGCTCAGCGGTTAAGAGCACTGACTGCTCTTNCAGAGGTCCTGTGTTCAATTCCCAGGAACCACATGGTGGCTC

ACAACCATCTGTAATG

>Sequence_403

GATCGACGATGACCCGCAGTTCGCCGCGCATATAGGTG

ACCGAGGCGCGGTCGCCCTCGGCGATGGT

>Sequence_405

GTAAAGCCTACTT

>Sequence_406

AGACGTTTCCTCCAGAGTTAACAGCTCCTCAA

GTCCCTCCCTAGAATATTNCGAGCACAGCATGTTCCAGGCATGCAGCACCTGTTATCCACTTGTTTAAATGTCGCATGAA

GAGGACAGAGAAAAAAATAGTTTCGCTCCATCTCTGGGAACATACATAAATTTAGCATACAGTTCTTTTTTGGCCTCTTC

CAACTTTGAGTTACCTTAAGAAAGGCCATAGGATATAATATCTTGAAGATACTCTTCACATTCCAATTTTTTCCCTAAAA

TGGCTGAGAACAGGTGGTGTCCCAGAAAAGAAATAAAAAAGACGGGGGTGGGTGGAGAAATGAAAAATACAATAGACGTT

GTGTTATAAATCCAGCCATAAAAACTGGGATGAAGCAGTGTCAATCAACTTATTACTAACATTTGCCTTCTAAGTATTTG

GTGGTCTAATTGTATTGAATTTCAAGGTCATTAGGAGAGAAGAAACTGCTTTTGGAGATTAAAATCCACTGTATATGTGA

ACGGGTTGTTGACTACCGCTCTTTANGAAGTAGACTGGTTTTATATTACTCTNCCTGCATTTTAAGAT

>Sequence_408

TCTCTCTCTCTTTCTCTCT

>Sequence_409

GATCTCATTACAGATGATTGTGAACCACCAGGTGG

TTGCTGGGATTTGAACTCAGGACCTTCGGAAGAGCAGTCAGTGCTCTTAACTGCAGAGCCATCTCTCCAGCCCCTGAACC

CAGGTCTTATGTAACAGCATCCGATGCAGGAGTGTGGGGCAATGGTCTATGCACAGACAGTCTGGTCTCCAGTTGAGTTG

AGGTCTGAACCTCGGTAGAACCCAATGGTGATAATTCACCTACAGGGGACAGAAGGTGTTCCCTCATGTGTCCTGGACCC

CTGGCTACTGTCAAAGTTACCGCCCCCACAGCCCCCACAGGAGAAGTTGTGGCTAGCAGTCACATACAAAATGTCCCAAT

CTTCTGACCTTCAGGCTAAACTCCTCCCAGTTACCTAGCAACAGCAAGATAACAATCCCACTATAAGAGGGGCTGCTTGG

CCCCTCCTCCTCTCTTGCTCTCTAACCTCTCACTCTCTCTCACCTCTTACTCTCTAGCCTTTCCTCTCTCTCTTTCCCCC

CTTCCTCTTCTTGGCCATGGCTGTTCTCTCTCTCTCTTTGACCTTCTCCTTTCTCCCTGCCTTTCTACCATAAAGCTCTA

AAACCATAGACTGTCTCTGTTCATCAAGGCCCGCTGTGCTTGGACGATGGGAAAA

>Sequence_410

GATCAGATGCTACTAGTGAAACCTCTGCCCTGAAAAAG

CTTGGTAACTTCCTCCTTCTAAGCCCCAGAGCCACGTGATGGCCATCTGCTTCCAGCTCCTTGTCTGCTCAGGAGTCCTG

TACAGGAGCACTGCCAGCTGGAACAAGGTGTGTTCTCACCTTGACCAACACCCAGTGGGCCCAGCCTCCCCGAGTTTGTG

CATATACTTCCTCCCAGATGGAAGGCATTTCTTTCATCTCCTCGTGGCCAGACCAACTCCATCTTTCAGTTCTCTACTCA

GTGCCCCTCTCCCAGCTGTTCACGATCCTCTTCAGCTGGGAGCCATCACTCCCCTTTCCAATTTTCTTTTAGGAAACAAT

TATTTTCTCTCTGACATGTGACATTTTAATGT

>Sequence_412

GATCAGAGTGAGGATACTTNTTCCTCCTTAGAATAGG

GTACAAAACACCCATGAAAGGAGTTACAGAGGCAAAGTTTGGAGCTAAGTCAAAAGGATGGACTATCCAGAGACTACCCC

ACCCGGGGATACATCCCATAATCAGTCACCAAACCCAGACAGTATTGCATATCCCAGAAAGATTTTGCAGAAAGGACCCT

GATATAGCTGTCTCATATGAGGCTATGCCAGTGCCTGGCAAATACAGAAGTGGATGCTCACAGTCAACTATAGGATAGAA

CACAGGACCCCCCATGGAGGAGCTAAAGAAAGCACTCAAGGAGCTGAAGGTGCCTGCAACCCTATAGGTGGAACAACAAT

ATGAACTAACCAGTACCCCCAGAGCTCATGTCTTTAGCTGCATATGTAGCAGAAGATGGCCTAGTCGGCCATCACTGGGA

AGAGTGGCCACTTGGTATTATATGTCCCAGTATAGGGGAACTCCAGGGCCAAGAAGTGGGAGTGGATGGGTAGGGGAGTA

AGGTCGGGGGAGTGTATAGGGAACCTTTCGGGATAGCATTTGAAATGTAAATATAGAAAATATCTAATAAAAAAAAGAAA

TAGATAAATCCTCAGATACATAATGCATACCAAAATTAAGTCAGAAAATAAAAAGCAGCTTAAACAGTTNAAAATATGAA

CGAAGCACCACACTGAATTAATAGTAAGGCAGTCTCAGAGCA

>Sequence_413

GATCCCTGGNATATGGCAGTCTCTACATGGTCCATC

CTTTCATCTCAGCTCCAAACTTTGTCTCTGTAACTCCTTCCATGGGTGTTTTGTTCCCAATTCTAAGGAGGGGCATAGTG

NCCACACTTCAGTCTTCATTCTTCTTGAGTTTCATGTGTTTAGCAAATTGTATCTTATATCTTGGGAATCCTAGGTTTGG

GGCTAATATCCACTTATCAGTGAGTACATATTGTGTGAGTTCCTTTGTGAATGTGTTACCTCACTCAGGATGATGCCCTC

CAGGTCCATCCATTTGCCTAGGAATTTCATAAATTCATTCTTTTTAATAGCTGAGTAGTACTCCATTGTGTAGATGTACC

ACATTTTCTGTATCCATTCCTCTGTTGAGGGGCATCTGGGTTCTTTCCAGCTTCTGGCTATTATAAATAAGGCTGCTATG

AACATAGTGGAGCATGTGTCCTTCTTACCAGTTGGGGCATCTTCTGGATATATGCCCAGGAGAGGTATTGC

>Sequence_414

GATCATCCCTTTCCTGTACTTCTTACTTTCTTAATTTTT

TTCTCTTGTCCATCCAAGAATTCTCCCCAGGAAGAGATGAAATTGGTCTTCTGATTGCATCCACTAA

>Sequence_415

CTGGTCCTGGGCTTTTTTTGGCTGGGAGACTA

TTAATAACTGCTTCTATTTCTTTAGGGGATATGGGACTGTTTAGAAGGTCAACTT

>Sequence_416

ANGAGAACTCTTNCTCCTGTGACATTCCTCAGAAGGCATGTGCTTAGCTGCCCGCATTCTGGAAGCTCACGAGGAAGATG

GACGGCATGGAGGGTCACCACTCAGAACACGAATCCACTT

>Sequence_418

TGAGGGTCCCCACACTCCAGCTCTCTCATCTG

CCCCACTCAGTCACTTATGGCACCTCCTGAGCTCCCCACAAGCCTATTCCTCTGCCACACAGGTATTGTCACAGTACTCG

TTCGTCTCTGGTTCTTAGTCCATGCTTGAATGCTTTTTCCCTCTTTCTGCCCAAATATCTCACACTTTGTCTTATCATGA

ATGAGCCTAAGCTCTCTGCCTTTAACAGAAACCCATAGCATTCCCACCCTGCCTTTCCCTCCATTTGCTGTTTTATACTT

CTTTGTTGTCTAAAGACATTTTATTTGTTCTTGCCTTTCCATGTTCGTTCTTGCACATGGCACAAGAGCAGGGATGTCTG

ATTTTCTGATGTGGCCAATAAATG

>Sequence_420

GATCTACCACTATACTACCATCTTCCACATCTATGAGACCCCTTAGAA

CTTGTGGTTTCTCCAGGCCTTGTGCTTCTGCTCTGCTTTTCTTCTTCTTCCTCCTCTTCTGCATCCTCTCTCTCCCAAAC

CTCCTTCTTCTCTCACTCAACCTTCTGTTCCCTTCCCTTTCTGCCCAATCATCAGCTTTCCTTTATTTTACAAATTAAGA

TGGGGGGCAGGTTTACAGGAAATCACCTGAATGCTGACTTATTCCTTGTTCACAGCCCCTCACAGAAGAATGGAATTAAC

ATCAAATATAATTAGCCCCAGGGCTATCCACAACACGCACATACTTATGGACACTT

>Sequence_421

GATCTAAATAACTTTAAAAGTCCCACAGTTTTTCCAAAG

TCTTACATATTAAAATTTCAATCCCTTTAAAATACCCAGTCTTTTAAAATTCAAAGTCTTTTTACAATTCAAACTCTCTT

ACCTGAGGACTCTACTAAAATACTTTCTTCCTTTAAGGAGGGAAAACATCAGGGCACAGTCACAATCAAAAGCAAAATCA

AATTCCAATCTCTGGGACCCACTCACAATCTTCTGGGCTCCTCCAAAGGGCTGTCGTCACTTCTTTGGCTCCGCCCTCTG

TAGCACTCTAAACTCTGGTT

>Sequence_423

AACCAGCAAGCTCAGCAGAGGCCTCCTCACAT

TAAATATAGATTAGGTCTCAGACCAGGAAGTGACTGGGGAAGGCCAGGCACCACACACAAATCTCA

>Sequence_424

GATCTCGGGGTCCAGGTTGATTGAGATAACTGG

TCCTCCTACAGGGTTACCCAGCTCCTCAGCTTCTTCCAGCTTTCCAGTAATTTAACCACAGGGGTCAGCAGCTTCTGTCC

ATTGGTTGGGTGCAAGTATCTTCATCTGACTCTTTGAGCTGCTTGTTGAGTCTTTTGGGGGATATCATGATAGGCCCCTT

TTTATGAGCACTCCATAGCCCGAGTAGTAGTGTCAGGCCTTGGGACTTCCCTTTGAGC

>Sequence_426

GATCCTTCCTTTGGATAACATGAGAGACTTGAGGGTGG

CCATGATGCCCAGGAGAAGGCAGGGGCCCTGCTGAGGAGAGAAACTCTGCAGACAGCAGAGTGAAGACGCCTCAATGGGG

GCAGGGTAGCCTGCTGCGCTTGTTTTTTTTTGTTTTGTTTTGTTTTGTTTTTTTTAGGTTTGTTTATTTATTGTTATATG

TAAGTACACTGTAGCTGTCCTCAGATGCACCAGAAGAGGGCATCA

>Sequence_427

GATCCCAAATAAAGACCATTGGTATGGAAGGAAAAAGA

ACTGTTCCAAGACATTGAAAGGAAGAACTAGAAAAGCCTTGAGGCTAACTAGGATAGGCAGATATCCAGGGCATACAAAG

GACTGAACCCAAGGC

>Sequence_428

AAATCAGAAATTAAGGTTCCAAGGAACCAAAT

AACCCTTTCTAAAATGAAGTAGAGAGCTAAATAGAGAATTCCCAACTGAGGAATCATGAATGGCTGAGAAGTCCTTCAAG

AAATGTCCAACATCATTAGTCAACAGTGAAGTACAAATTAAAATGACCCTGAGGTTCTACCTCATACAAGTCAGAATGGC

TAA

>Sequence_430

GATAAAAGTTTTNGAAA

>Sequence_431

GATCAAAGCAGCTGTGGTCTTCTTCCTTCGGTCTGTAA

ATGGGCGCAATGCCAGGTGCTCTGTTAGCAGCAATAACTAAGGCTTNGTCTTCCCTTTGAGAGGAAGAGAAAAGTAAACA

CATCAGAGAATAGGGAGACTCCTGATGGTGAGTCTCTAGAGCCCCA

>Sequence_432

GATCTGACTCCCTCTTCTGGTGTGTCTGAAGACA

GCTACAGTGTACTTACATATAATAAATCAATCAATCAATCAATCAATCAATCAATCTTTAAAAAAAAAAAAAAAAGAAAG

TGATGAAATGACCCTTTAAAACGACCAGAAACTCTGGAGTTTGAAAGCGGTCTACTCACCTAGCTCCTGGTGTCTACCTG

GGGTACTTAGCACACTCCTCACATAGCACCTCGGGCCCACTTTCCCTGGGCCCAATGATTGATGACAAAGCCACAGAGAC

AAAAGTAGGGGGCGAGACTCCTGCATGTCCAAGAAAGCAGTCCAGAGTTCTGAACCTTGGCTCTTAAGGCAGGGAGCCCT

TCACCCAAAGGCCTGTTGAAAGTAATATGAGCAAAAGGGGCCTCTGGAGATGATACCCTCAGACCTGGCAGCTCCTGGGG

TTGTCACTACCTACCATCCTTGT

>Sequence_433

GATCAAACGTAGAACTCTTGGCTCTTCCAGCACCATGTC

TGCCTAGATGCTGTCATACTTCCTGCCATGATGATAATGGACTGAATCTCTGAAACTCTAAGCCAGCCCAATTGCATGTT

GTCCTTTGTAAGAGTTGCCATGGTCGTGGTGTCTCTTCACAGCTATGGAAACCCTAACTGAAACAGAAGTGAAAGTAATA

CTCAAATGAGCTGTTGAACTGAAGGAAAAGCTGGAAAGCGATGGGCCATTAGGCATACAACCCTTTGTACGACATTTTCA

TGTCCTCCACCTCTCTCAGATTAGCACCGTGGGGCTTTTCTCTATGCTCTGTAAGAAACCCAGTGAAGAAGGGATTCCAG

TCTTCTTCTGCAAGTGCATTTTTATATGTTAATTGGTTTTATTGCATGCACCTGTACACATGTGTTTGTGTCCTGGCACA

TGTATACACTTCAAGACACAACTTTGTGGAGTCAGTTCTCTTCTACCTTTATATGAGTTTCAGGTATTGAACTTGGATTG

TACGGCCTTATCCAGAGCAGCAAGTTCTTATCCACTGAGCCACTTGTCAGCTTTTATCTTGACCTTCTGTGGGGAGTAGT

TTGAGGCAGGGTCTCATGGTGTGGCCCTGGCTGGCCTGAANCTTTCTTTATGAAGCAGGTTAAACTTGAACTCAGTAGAT

CCCCCGGGCTGCAGGAATTCGATATCAAGCTTATCGATACCGTC

>Sequence_435

GATCTGAGTGCAGTTCTTCAGGACTGATATAA

AAATCTGTAGGGTGTAGCCCTGTGCCTGTAATTTCAGTGGGCAAGGTCAGAGCAGGAGACAGGCAGAACCCTG

>Sequence_436

AGCCAAGCTGTGTCTGTCAGCTTTCCATATTA

CAGAAACCCGCTCTTTCCTGCAAACGCTGGGTGAAAGACGTTCAATACATGATGAATTATCACGTCCATAATCCCTTCAT

TAAAAATCACTGATTCTGATTTATTGAATGCTTTGGTTGTGAGGGGAAGAAGGTTGCATTTGGGTATGAAGTGCCCTTCC

AGGAGGCCTATATATTGAAAGGCTGGTCTCCAGATTATGGTAATAGTTCGAAAGGCTTTGGAAACTTAGGAGGTGGGAGC

TGGTTGGAGGCGACAGAGATAATATACCCTCTAGATCCTCTCCTGTCACTCTTTGTTGCTTGTCTGTGAAGCAGCAGCAG

ACAGCCTCCATGACACACCCCCATTGTCACAATGCTCTGTCCAAGGACAGGGCTCAGGCAAGCGTGGGCTGAATGTTCTG

AAACCAAAAGCCAGAATGAGCCAGTA

>Sequence_437

GATCTGAAGTCGAATTGAAATCACAGCTTTTGTTCACA

AGGCATAAATCCACCAAGAAATTCAGNGAATCAACAGCTCACCCTCACTGTACAGTATGGACCATGAAAGCCTGTTTAAA

ATATATGAGAAAATTCACTCAGAAAGTCACAGGACTCCAAAAATAACAACTTTTGGTATATATTTATATAAATTAACACA

TTATGTTAAGTGGCTATACTTCANTACATTAGGATAAGCTTACACTGGACCTTTTCCTTGATTGTGTAACTGACTGGTTT

CGTTGCTTCCATTTCNGAGCATTATATATATATATATATATATATATATATATACTTAGTATACATATATATATATATAT

ATGAACATTGTACTTGTTATATGATAGACTCTGGGCCATATACAGTGTACCGTGTTTCCAGCTAAGTTGTGTTNCCACAG

AAGGTATCA

>Sequence_439

GTCGGTACAAATTTCGGGGCGCAGCNTCTGGTCGCGCTCCGATGCGGGGCTGCGGCTCGGCCTTGCGGTGGCGCTTGGCT

ATTGCCTCGCCTTCGAGGCGGCGATGCTGCTCTCCGCCCCGGT

>Sequence_440

GATCCAACAGNACAGAACGCAGGGCTCTCGTCAGAACT

GTTAACTAACAACAGGAACTTGTTAACAGATTCCCTCCCACTCGTCCTCATTCCTCTCCTCTGAGGACTTTGGTCTGGAA

TTTGTGGACTGACTTCACCCTGGGCAAAAGCAGGTTCAAAGCGTGCTAAGGCAGAAGGAGATTTCGCAACAGTGTGAAAA

CCTAGGGAAAGAGCAGAGCGACTGAAACAGAGAAAAATACGTGAGAAGTCAGAGCTGCTTTACCTATCTCTGCTCATCTT

TTTAGCTGTGTCTCAAGAGGCCACTCCTCCGCTCTGGAAAGATGAGCAGTACCTCTCTGGTAATCCTCGTGTGCAGTGGG

ATCCAGAATCTTCCATATTAAGTGTTCAAGCAGGACAGTGACAGGAGACTAAGCTGATGAAGCATCCTCAGGGCACAAAC

ACCTAGAAGCAATGAACCATTTGAGAGTCACATTTGTAGCTTTCAAATACCTCAGAAAAATAGAACCACTTTTGTGTGGT

GAACTA

>Sequence_441

CTGTGGTGGTTGTCCTAGCTGGAGTGGGTATT

GGGTTTCACCCATCTTTGATGACTGAGGACCTCCTGGGTGGCTTGCAGGCTGTAGGAGTGGATGGGTATCTGGCACTGAT

CTCAGAAAAAAAATCAAAACGAGCTATCTTCACTCTCTAGAG

>Sequence_442

ATTTCCAGAGTGGTTGTACAAGCCTGCAATCC

CACCAACAATGGAGGAGTGTTCCTCTTTCTCCACATCCTCGCCAGCATCTGCTGTCACCTGATTTTTT

>Sequence_443

AGANGTGAACAGGCNGGAANTCNTGAGGTCCGTGGGNACTGGGCTTTTACTAATCGCCTTNTGAAAACCCTCCTCGAGTA

GCAAAGCAACACATTCTGA

>Sequence_444

GATCCGAACTCAGGTCCTCATGCTTGAGCAGCAAGCATT

CTTACCCACTGAGCCATCTCCCTAGCCTCCCAGCTTGTTTGAAAATCCCTGACAGCACCTAGCTGTTCTGTCCCCAGCCC

TCCGGACAGTGCTGTCTTTGCTGTTTTCATTCTCTGCCTCTGTGAGGTGG

>Sequence_448

TTGTTTTTGCAGTGTTTGAATTACACAGCTGT

TGTTCTTGTCCACAGATGCTCTGCTGTGGACATGGGAACCTGTCCCTCCTTTCTAGAAGAGCTTANAGCCCTGGGATGTG

GGTATAGTAGGTTGGTCAGTGATATCTTTTGAGTTACTGGTGTTACCAGTGAACCACATAAGATGAACTTCATTCCNAGC

GAGTATACAGCACCAGAATATTTAAATAAATATGTAGAAAGCATCAATGGGGAAAAAAACTCTATGGAAAACATATAATG

TGAGTTTGAAGTGTTCTCTTATTTTCCCCAGGATGGTTGCTTGTGGTTTGTCAAAGTATTAGCCACGTTACACACACGTG

AGATTAAAGAGGCT

>Sequence_450

CTCACAGAAAAAGAAAAAGGATGGAAATTTGT

NTTTATTTTTAATTTTTGTGCTAGGAATTGGACTCATAACTTCAAGCATGCTAAACACAGGCTCTTCCTGCAAGTCCCCA

GCAACAGAAAAAACTTTACCAGCCTTCTGTTTGGTCCCGTAGTTCCTGCC

>Sequence_452

GATCCTTCTTACTGCCTTCTAACCAGGAATGGCA

GTTCTAAGAAATTTCTTCCAAGGCAGTAAAAAAGCAACATCAAATTTTAGGTAGTTTTTAGTCTCTATAAGGCAAATTCT

CTGATTTCCTTTAGAAGAAATAAAAATATTGACCCACATAAACTAAGAACTCACATATTTCCCTAAGAAATTTGTTGCAG

GCAAACTTGTGTATGTGTGTGTTTTGGGGAGGTGTAGAGGTCAGAAGTTTACCTCAAATGTCTCCTTCAATTTAGCTAGA

TGGGCTGGACAGCAAGCCCCAGGGATTCACCTTTCTTCACCTCCCCAGCAATGGAAGCATGGGTATATACTGGTACATCC

TGCTTTTCTTTTACAGGTACTGGGGTTCAAACTCAGGTCCTTGCTTGCACTGCAAGCTCTTTACTGATTGAGTTGTCTGT

CTCCCAAGCCAATGCAAAATACCCTCAAATATCTCAGCATAACTAAAACTCCCTCCCCTGCCATCTTATAAGTTCTTTAA

GAATTTCACATACTGTATTT

>Sequence_453

CGTCGTCGTCGGACANTGTTGTCGTCGGCGGCTGCAACCATGGCGTCGACCA

>Sequence_454

GATCAACTTCTGCTAGGTGTGAGGTCAACCTGGCCTTCC

CAGCAGCTTCAAGCTGACGCAAGAGGCAGTGAGGCCAATATCAGAGTATTCCAGCAGTGACACGGACAGGTGACCCTGAA

ATGCTTGTGCAGAGCATGTCTACACCAAAAACCTTGTTCCCCAGAGCTTCTAATGTTGTAACCAACACTACAAACTAGTA

TTTCTAGCACTGTAGCCAATACCCCCCACCCATCCACAGTTAGGGAAAGGCTCCTGCATGGTAGCCCCATCTGAAACCTG

TCAATCAAAAGTAGAGAAGCCTGGTCCAGCTGTGGGCTACCCTGAAGAGTCATTTCAGCTTCCAGAAACTACTGGGTCAG

CTTAAGCCTGCACTGAAGCCCAGCTTTCACTATTCCCAATCTTGCCTCCTTCCCTTCCTTCCGAAAGGCTTCAGCAAATG

TCCTGCGGGCCAACCTCCTTCCCAGAGCCAGCTCTCCCAGAAGGCCAGTGACCACACAGACACACACGCACATGTGCACC

AGACTGCCAGGCTCCAGAGCAGGCAGGCAGCTTCCCAACACTAACAAAGACTCACTCCCTCACTTAGGGAAATGACAAGC

AAGTCATTTCCTCTCTT

>Sequence_455

GATCTAACCAGGACTGCAATGGTTTGAGCGTCCATGGCTATGAGT

GTGAGTTTCAGATGATGTCCATGGCTCTGAGTGTAAGCGAGGAGTTTGGGATGTTTCTAACACTTTTAGCCTAAGAAACT

GGAAGGACATGGTTTCTCTCCATTGAGAAAAAGGAAGGGAGAGGCATCTGGGCAGTTTTGGCTGAAAGCA

>Sequence_456

GATCTTTCTCATACACCTCCCTACTGCCCAGTCCCTCCC

AA

>Sequence_459

GAAGGAAAGTCCATCCAGAGACTGTCNNTCCT

GGGTATGCATCCTATATACAGCTACCAAACCCAGACACTATTGTGGATGCCAACAAGTGCTTGCTGACAGGAGCCTGATA

TAGCTGTCTTCTGAGAGGTTCTGCCAGTGCCTAACAACTACAGAATTGGAT

>Sequence_460

GATCACCAAATATCCTCCCTGCTGCCTTCGTC

ATTCTGGCCACAAAGTCTTTTTTCTTTTGTTTTTTCTCCTCTTTCCTTTCAATCTCTCCCCAGGTATTCTTCCCTGACAT

TAACTTTTCCTCGGGTTCAAGACCCGTGGAAAGACCTGTATGCTTAATTGATGCACCATGTTTCCTCTTTGCTTCTACTC

TCTCTCCCCGCTTTACTTCTGATAGACTGTCTTGAATTTCATCCAGAATTCTCTGCCCTACCTTAACCACTTGATAACAT

GTGAAAAGGAACAAAAAGCCTCCTAACACTGGAGAAAGTTCAAGGCCAGACATACCTCATAAAGCTGTAACGTGACCCGC

AGTTCTGGTTCCGCCTCATGAAT

>Sequence_461

TGCAGTGTGTATACAGTAAAAGGCTTAGATGACAATCCCAGGAGACATTCAGACTCTTCCG

GGGTCTTGTCTGGTATTGCCAATATGAGGCTCCCATTGCTCTCAAGTAAACTCGGCCTTCAACACCCCCTTCAATCTCAG

AGCCCTGTTACTTTCTGTGGGAGAAATCACAATACTTTTCTATTTTGGGAACTTCAAGCAGGTGGAAGTTGATTTGTGAT

GTCACTGTGGCTGTGACCAAGGGCTGTACAACCAGTATCATTGGCTTAGATGGGACAGACAGCGAAAGAAAATCTGAAAT

AGAACCCACCATGTGTGCAACTGCTAATTATTTGCTTTGTTCTCGCAGCTCGACACTAATACATTTAATATCAGACATTT

ATAATCTGGCACTATTTAAATGACTAGAGAGAAGTCTAAAAGGCGTCTCATAGCTGGAAGCCCAGGCTCGTTACAAAAGT

TATTCTCTGAAGTCAGTGTGGAAGCGGGTGGTAGAATCCATGTTGCTTTGAGACTCTCAGACTATGCATAAAATCAGCCC

GATTGGTTTCAAAGATGGAAAATAGAATTTCCATGGATTTTCTAAAACAAAATCTTTTTTCTAATATTAACCAAAAGGAT

TGGTGAACCTTTCTCAGGTCGACCAGCTGTCTCGATACGATACTGGAATAAGCAGAGCCCAGTGCTCTTCTTGAAA

>Sequence_462

GATCTAGTGGTCAGTTCCAATTGCTTTCTCAG

TACTGGTCTAAATGCTCTCCTGTCTTGAAACTCCCTTTGCTCAAAAACCTAGTCAACCATCTCCCAATTCAACTTCACTC

AAGTTCTTAGAGCATATGCAAAAATGAAGCCACGTTCTT

>Sequence_464

NGCANCCTACCCACATGCTACACTACTTGGGAGGCAGAGAATTAAGTTTGGGGCTAGCCTGGTCTACAAATACAGTTCCA

ACTAGCCAAGGCAACATATTGAGCAATCTTTAAGCCAGCAAGATTAATTTAAAATTAATTAAATGATTAAAATGATTACC

ACCAAGCCTATCAACTGGAATTCAATGCCTGGTACCCAGAGGAGAACAGACTCAAAACTGGTTGTACCCTGACCTCCACA

TGCNGACTGTGTCATGTACACACAAAAACACCAATCTTCTAGGAGCCCTAACTATATTTACTTGCACACAAAAATAATCA

GCATACTTTATATATCAAGTTTTCATAGAAAATTCCAAAAACTATTGTCTGTATAACTGTGAAAATATTATCTGGGGGCT

GAAGAGATTATTGCAGGTAAGAGCACTTGCTGCTCTTGAAGA

>Sequence_467

AATGTTACCCTGGCATATATTCACATTTATTTT

ACTAGCCACTAAAAGTCGTATTTCATCAGTNAGCTTATCTTTAAAACCAGTATCATCCTGTTTTCAACTATTAACTAGTT

TTTCCTTGACACTGTATAGCATAGAGTGGAGACGGGAGATGCATATTCTAACTACTTCCATACAATTTTTCTGTTTATTT

TTTTAAAAAACTACAGGTTATTTCAAAATAAGATATATAGAAGAAAATGTAAAAAACAAACAAAAATCAAAAACATCTGC

TGATAAATTAGGAGAAGTAAATAAATGAAGACTATCATATTTAAGGGATTTAGGACTCATTGGCAAGATGTCATTCTTTC

CGGATTTGAGTTACAAGGTCAATGCAACCTCCAAATCTCTCAGTGAGCTATTTAGTGGTTATTGACAAACTGAGTAGAGA

ATTTATATTTAAAGACAGAATATTGAGACTAAGAAAAGGATAACTTAGAATTCTGACACCATCCTATTTCAAGGCTCATC

TTAATGTTATCTTCACCAAGGGTGTATGTGTGTGTGTGTG

>Sequence_468

GATCCTGATTCAACTTTGGTACCTGGTATTTGT

CCAGAAATTTTTCCATTTCGTCCAGGTTTTCCAGTTTTGTTGAGTATAGCCTTTTGTAGAA

>Sequence_469

ATGTTGCTTCATTGCTCAAAGCACGCTATTCA

GAATAAAAACCAGCATCCTTGCAATGATTAG

>Sequence_470

GATCAGTCTACAAACAGAGGCGTGACCTGCTATGCACTTTATCACTTGTACTAAATGC

AGCATAGTTATCGCA

>Sequence_471

GATCAAAGGCGTGCGCCACCACTGCCTGGCAAGGGCACTCTTGA

GAGCAAAATGCATTCCCTTTTGTAAGCTATCAAGACCAGGAGACACATGTGGAAAGAACTTTCTATCCCTAGCAGACAGG

AAATCAAACCCCCTTTTTCCTTGAACTTGGATAGTCTCCCCCACTTGGAAGTCTCTGTTGGCCTTGGGTTTTGGAGAACT

CTACTGTGTTCTCACCAAGCACAGAAGAATTAGGAACATCCCACTTTTCAGGAAATGCAGCAACTCTCTTTTTCTTTGTA

CATGCATATATGCTGTATTTGTATACATGTTCGTGTGTATGTATACGTGTGTATGTGTACAGATGTGTGTATATGTACAC

AGGTATGAAGGGCAGACGGTGACTTTGATGTCTTTTCCTCTATTGCCCTTCATGTTGTTTTTGGAAGCAGGGTATTTCCT

GAACCTGGACCTGGACTCAGCTAGACCAGCTGGCCAGTGAGCCCCAG

>Sequence_472

GATCTGACTAGACTTTCACATAAGTGGCAGTAAGGG

ATGCTAATCANCCCAAGAACTNTCAAGCCAGGTATAACTGTATTCCAATCCC

>Sequence_473

GATCTACAGAGTTAGTTCCAGACAGCAAGGGCTACACATAGAAAATCTGTTCC

AACAAACAAACAAACAAACCAAACTAGAAAACAAAAAACAAACAAGCAAAGCTAATGCTAGAAAGAGAAGCTTCTCTTAT

TAAGACTGGGAGTTGCT

>Sequence_474

GATCTGAAAAAATAGAATACAGAGATTTTTTAAAAAATTAGGAGT

ATAGGTTTTTACAAACAAGATTTTATAGTCTTCTTTATAGTTCAAGTGTTACTGAAGACAACTATTGATAATCTTTGAGA

AATCTTTATCAAAGCAAGAGTTGGACATAAAAACAAGTGTGTGTGTGTGTGTGTGTGTGTGTGTGTGTGTGTGTGAGTGT

GCGTGTGTGTGTGTGTGATTGTGTGTTTGTATGTGAGAGAAAGATTATAGTTAATTTTAAAGAGGTATTTGCAAAAACAA

TATGAAAGTATTTTTTAACAAAAAGTGTTGTTTCATAGTCAATAATTTCTATAGTGGTTTATTATTAGAAAAAAATCCAT

ATTGGAGTTTTGCAGTTTCACAACTTTTACAGAACAATAGTTCACTACTTGCTCTGTTATACATTAACACATTGGCTTTC

TCTCACAAAGGAATATTAGGCAAATTAGCCTAATATTAAAAAATAAGTTAATATACTAGTATATTATAATTTTATTATAC

TAGTATAATAAAATAATATACCTAGATGGAATGAAATAATAAAAATCTTAGTGACAGTCGATTCAAGAAATTTATTAACC

TGTTAGGATGATATAGTTACCTTA

>Sequence_475

GATCCTCTCTTTAAAGGTGTCAGGCTCCCAATGG

ACCAATCCAGGTATGTATCCGTTTGTATATTACAACTCTGTGGTGATGGGAAGCTTGACAAGTGAATGCAATTCAGAAAG

AGTCAGAGCGAGCCAAGGCAACAAAGGTTATGCTGGGTTTCCCCACTGGTCCCTCAATGTGAAGCTGTGCTTCTAGGAAG

CTTTGACATCATCTTCACTTTGGGAGGAAGTAGAATTGATCAAAATAGTCTANTCAGTTACCAAAACAAACACACAAGTG

AAAAACAATAATAATGGCTGATTGGAAGCACTTGCTACTCTCGCAGTCCAGGTTCTTCGTGGCATCCATATCAGGTGTTT

CACTACTGTCCATAACTCCAGCTCCTGGTACTCCCCTGCACTCTTCTGGCAACCAAGGATACTGCACCCACCAGCACAAA

ACCAAGCACAAACACTCATAAGCCGCACATCATTTTATAAAGGATAGAAACCANAACGTAAACATTAACAAAATTGACAA

GCCCATGTAAAAAACAGAGTTGTCTATCTCACAATGTCTATTCAGCACTGTAGGAGGTTTTNGGCTGGAGTAAAATGAAG

CAATATTTTAAATTTTAGACAGCAATTAACCATGCCTGTTAGCAATAATA

>Sequence_477

GATCCACCCCATAATCAGCATCCAAACGCTGACACCATTGCAT

ACACTAGCAAGATTTTATTGAAAGGACGCAGATGTAGCTGTCTCTTGTGAGACTATGCCGGGGCCCAGCAAACACAGAAG

TGGATGCTCACAGTCAGCTAATGGA

>Sequence_478

GATCCTTGGCAGAGTCCTGTAGAATCCAGTTGGGTGTAGTGG

CAAATCTCTCATATCTCGGCACTCACACTGGGTGGGTGGAAAGTTGAGGTAGGAGGTTGGTGAGTTCAAGAGAAGCCTGA

GCATAGCAAGGCCAGTTCTTTGGGGTACAGCGATTGGTCCACTTTATTTCCTAATGGAATAGGTGGTCTTCTAGATGGGG

AGAGGGCTTGTCCCTCTTGCTCCAACCCACTAATAAATAGTGACTTGCAAAAGAATAAAATAAAAAGCAAAACAAACAAA

CAAACAAAAAAAAAAACCTTAGGACCAAAAAACCAAAGCAAAGCCATCCTAACTTTGTTCCAACTAAATACAGCTCTTCA

>Sequence_479

GATCTCTTTTCTTCCTTCAAAGCACATTCAACGTGAGCTCTG

GGAGATGTCTGAGGAGTGAAGCTGTCTCTCACAGCCAGCCGTCACCCAGCTGTGTCACCAACTGCACTGTTACTTTTGTC

TGAGACAGGGTGTTACTGTGGCTGACCTGGAACTCATAAAGACCAGGTTGGCTTCAAACTCATGGAGACCTGCCTGCCTG

CCTCTGCCTTCCAAGTGCTGGGCCTAAGGCATTTGCCACTATGCTGGGCTTCATTTCTTAATATGTCAGCAGTTACCTCT

TTCAGTTTTTATGTAAACAAGATAGCAGTTAAAAAAACAACAACAACAAAAAAACCCATTCATCTTCTGAGTTAGTGATC

AGTTCTATAAACATATGCAATTTCCCTTCTTCCTAGCTGCTTTGAGGAACTTCCCTGCTGTGAAACAGACTGAAGGATGA

GGGGTCTGCAAGGTGGAGATATAAAGGCAGGCACACTAAAATCACATCTACTGTTTTCATTTGATTTAATCTAACAGGTA

TTCACTCGGGCATAGGATATAACTCAGGTTTAATGTGTCTCTGGCTGTGCATTATCCTGTTACGTACGCTGGGATACCTC

ACAACTTATTCTTTAAAAGCTCATGAATTAGGCTTGTGCTATAGCTTGGCACTGANGTATTTCCCTAAGATATATAACAC

CCTGGATCCCCCGGGCTGCAGGAATTCGATATCAGCTTATCGATACCGTCGAC

>Sequence_480

GATCTTGTCCAGGAACAACTAGATGTATTATGGCAAATAGCTCAGCTGGGAT

GTGAACAAAAGTTTCTGGGATTGTGTGTTACTTCCATTCAGTATGAGAAATTTACTAGGGCAGCTAATTTGTTAAAAAGT

CTTTCTCAGTATATGTTACAGAATTGGATGGCTGAATTTGAACAGACCCTTCGGGAATTGAGACTGGCCATTATTCAGGT

CAACTCCACGCGCTTGGACCTGTCCCT

>Sequence_481

GGGATGTGAACAAAAGTTTCTGGGATTGTGTG

TTACTTCCATTCAGTATGAGAAATTTACTAGGGCAGCCTAATTTGTTAAAAAGTCTTTCTCAGTATATGTTACAGAATTG

GATGGCTGAATTTGAACAGACCCTTCGGGAATTGAGACTGGCCATTATTCAGGTCAACTCCACGCGCTTGGACCTGTCCC

T

>Sequence_482

GATCCACCCCATAATCAGCATCCAAACGCTGACACCA

TTGCATACACTAGCAAGATTTTATTGAAAGGACCCAGATGTAGCTGTCTCTTGTGAGACTATGCCGGGGCCTAGCAAACA

CAGAAGTGGATGCTCACAGTCAGCTAATGGA

>Sequence_483

GATCACATACAGGCATGATATTCTGAGTTT

>Sequence_484

GATCAAAGACCTAAATAAAAAACCTGCAACACTGAAACT

GGTAGAAGAAAATATAGGGAGCCCTATATGATATAGCTGTAGTGAAGGACTTTCAGGAAAATAACTCTTTGTCTAAGAAC

TATGACCAACTATTGGCAAGTTGGATTAGTACAACTACAAGTGTTTTCATGGA

>Sequence_486

GATCCTCCGGTAGTACTATGTCCAATTTTCTGAGGAACTGCCAGACTGATTTC

CAGAGTGGTTGTACAAGCCTGCAATCCCACCAACAATGGAGGAGTGTTCCTCTTTCTCCACATCCACGCCAGCATCTGCT

GTCACCTGAATTTTT

>Sequence_488

CATGCGTTTTGAGTTCGCCAGTTGGCATCGTC

CGCGAGGCAAAAGACATTGAGTTTTTGTAGCGCCAGCGCCTCTCTTTCAATCCGCGTTTCGGGTAGCGGTGCGAGGCTGT

CTGTCAGGTGACTAATGGCATCATCAAGCC

>Sequence_490

GATCTCCAGCTTCTACAGGCTTTATCCTAAAACTCGTG

GTACCATTGCACCTAACATAATAAATAATAATATTGAATACTGGCCTCTATTCAGATTGCCCAGGTTTGTTCTAAAAAGG

CTTTCATAAGGATGAAATGAGATGGGACATCGTGCTCAGTTCTGTCCCTCGGGCTCTTTATCCTGTAACAGCCCTCCTCG

CGTGTTTCCATGTCTTTGTCCTCTTTGAAATATAAACTGAGACCCTTTTTGT

>Sequence_521

GATCCCTGGGCACCCCAAGGTCGGCTTGCTTGACAAAGAACAACTCAA

GGTCATGGGCACTTGGCACCCATGCGAAGCCAGCACCTGTCGTGTGCCACTCAAGACCTGA

>Sequence_523

GATCACATACAGGCATGATATTCTGAGTTT

>Sequence_524

GATCGTTACTCTTTTCTTTAGGTGTTTTTGGAAGGAATCTTCT

CATCTAACTGGAGTACAAA

>Sequence_525

GATCAGGAGTTACAAATGAAAGCATCACCAACAGAATACAGGA

GATAGGAGAATCTAAGGTGTAGAAAATTCCAAAGAACACATTGACAAAACACTCAAAGAAAAAGCAAAATTCACAAAGCT

CCTAACCCTAAACATCCAGGAAATCCAAGACATCATGAGAAGACCAAACCTAAAAATAATAGATACAGGAGAGAATGAAG

ATTCCCAATTTAAAGGGTCCGTAAATATCTTCAACAAAGTTATAGAAGAAACTTCGCTGATGAAAAGATAGAGATGCCCA

TGAACATAAAGGAAGCCTACAAAACACCATATAAAATGGACCAGAAAAAAATTCCTCCCATTACATAATAATCAAAACAA

TTAATATACTAAAGAAAGAAAAATATTAAAAATGATAATGGAAAATGGTCAAATAACATATAAGGGCTGACCCATCAGAA

TTACACAATCCATCCCAACTGAGCCTCTAAAAGCTAGAA

>Sequence_556

GATCAAGGGACTGGCCCTGGTCAATCCGGCGCAGG

ACTTCACCGAGCGGCTGATGTGGCCCGGTCTGGCC

>Sequence_557

GGCGGTTAGATTTGCCATCTGATTTCCTTTAG

CCACAGCATCTTAGGTCCTTCTCTGCAGATGTCAACAGCCCTCTTTGTATCAAAGTCACAAGTTCAGCTTTTTGGGCCGA

CGTCCCTTTAGGGAGGCTGCTGGTCTA

>Sequence_558

GATCCACCGTCAGGCGCATCGAGCGCATGGACTCTGCAAGCAGCGACA

GACCCTCGCCGCCACCCATCTGGTT

>Sequence_559

GATACAACAGGACTAATGCACATATGAACCTACAGAGAGAGTGGC

AGCATGAACAGGGCCTGCATAGGTTTAAGCCAAATGGGGCCCCAGGGCTAAGAGGGGAACATGGACAAGTACTTCTAAAC

TCTGTTTAATTGACATCTACTTGCAGGGAAAATCAGTTTTCTTTAGTGAACCCTTACTGGATGTATAAACCACACTTAAG

TGTAGGACTTACCCAGTAATACATGGCTCCTGCACAAACAAATGTATTTTGTATACTTTTGTGTCCTATTGCTTTGTTTG

GACATTTTTTGTCTTACTGGAGTTTTGTTATACAAACAAAATTCTGGTTTCTGATTGTGACTGTGTGTGTTTCTTGTATT

TTTCTTTAGACTTATATGTTTTTTTTCTGCATTGTTTGTTTTATTTTTTGCGTATTTGCCTTTCAAAGAGAGACGAAGGA

GGAAAAAGGGTGGAGGTTGGAGAATGAGAAGATTGTGGAGTTTCTGAAAGGAACTGGAGGAGGAGAGGAAGGCAT

>Sequence_560

GATCGCCCGGGACCTCGGCCTGTTCATGGCGCGCACGCTGTTCCGCGG

CTCCGACCTCGCTATGCCGACACGCGAGCGCAAGGCC

>Sequence_635

GATCTTCCTTCGCAGACTTAGACACTTTATTAATGTCTAATGAGATTCCATGT

GCAGGAGACAGTGAAAGAAGAGGAGACTCAATGCCCACTGGCGGATTGCTAATTCTCTGTTGCCATTCCTGATTCATCTG

CCATTTCAAGCCCCATTTTGCCCTATTTCCCCCTGCACTCACTCTCATCCGTCAGAATCCACTCTGTGTCCATATCAGGT

GACATGCTCCCAGTTCTTGAAATCCTCTTCTAGCTGAAGTTCATGATAGCATCTTACCATCGCCCCAAGTCTTCCCAGCA

CCCTCAGACTGCCAAGTTCTTCTGGACAAAACACAAAA

>Sequence_636

GATCAGCCTTCATAATACTTANTGATTTAAACCCCCTTTATTCCGTGCAAAATAATAA

CAGTTTACGAATTTTTAAGTCTCACATCAACAGAAACACTGCTTTCAGCATCTCTGGAGGTTGTTTTACTTTCTCTTCTT

TGTCCCACAGTCACCACACAGTCTTCCACAATTTGTCCCAGTTCCACAGGTGCGAGTTCTAGCTTATATTTAGAACACCC

CACACCTTAGCTTTCTATTTTCTTTATTATAGGCAGAAGTGAACTTCTACTGATGAAATATCTAGTCTGGCTACATGATG

ACTCATGTCATATATTATAGAGATATTCCCACATTGGGTAGATAATGAAATGTGCATAGTTTGGTGTTGTTTGACTGGGG

GGGGGATATATACAGTAATTCATTAATGAATCAACTTCTGCTCCAGAACCCAGAGAAGAACCAGCAGTATATTTTTAACC

CTTAAAGCAATT

>Sequence_637

TCTGCGAATTTAATGCTACCCTGGTATACATAGGGTTAGGGTTAGCCCT

TCCTTCGAAATGGATATGTTAGCATCGTAGTTCCTCAATTTTACTTTGACTGCAATATTCTCAATTTCCTCTACACTTTT

AAAGTATAGTTATGAAGGATACAAAGTACTACATGGCTGGCATTTTTTTCCTTCAGTCTGTTATCCAAAACCTTAGTCAA

CTATCTATCAATTAATTAATTAATTAATTTTTATTAATTGATTGATTGATTTGTGCTTGGCTTGTGAGGATGCATCATCT

GCAATTCCTATCTTTACTGGAAAGGTTTTGCAGTTCATTTACCTTGGTGGGCTCTTGATAGATTTCCTGTCCTTTTCCTT

GGTTTGCTATAGTTCTGTTATCTTAAACCTTGGTGTGACTTTGCTGTGTCCTGATTTCACTTTCTAGTTTTTTTGTTACT

TCTTTATGGTATTCTCTGAGAGCTCTGAATCGACTGTTTGGTGTCTCCTGCTAATTTTCAGATTCTCAGGCATCAGTCAT

TCATTTGTCCTCCCCCTCCCTCTTTCTTATCATGGTACTGTGTGTCCCCATGATACATGTGTGCAGCTTTGCAAACTCTG

CAGTAGTTGTGGGATGCTCGACTCTGCCCTTGTTGCTATTGNGTTAATCTTTATCGNCTTGCTTTTCTATTNGGACAGCT

TCTCTGTGATCCCCGGCTGCAGGATTCGAATCAGCTATCGATACCGCGACCCGAGGGGGGCCCG

>Sequence_638

GATCATCAGTGTTTAATTTTATGGGGTATCCAGAGAACTGTATGGAATATTTCTAGAA

CTTTCTTCCCTTCATCTTTGGATAAAGACTCATCATAATGGCCATAGTGTGTGCCTTTT

>Sequence_640

GATCCAGAGCTCTGAATTGGCCCACCTCAAAATCTATATAATCTGTGAGTGGC

TGGGACATGTGAAAGGGCCAGTCTTGTTGTTCCAAAGTTGCA

>Sequence_641

CTTTGAGGTTATATATGCCCAGTTACACACTC

TCCTTCTGCGGCTTTCATATCAAGATGTAGAACTCTCAGTTTTATCTCTAGTACCATGGTGTACCTAGATGCTGCCATAA

TTCCCATTATCATGATAATATAAGTCAGCCCCAATGATAAGAGTTGTATT

>Sequence_642

ACTTCTGAAATCTGAGCTCAGGATTTACTCCC

CTTAGGGAACCTTCCCAAAGGACTCCTCCAGAGTTTGAGACATCAAGTTAGTCCTAAAATCTCTAGAAAGTTGCAGTGAA

GGTTACACCTTTATGCAGTTTCCTCAAGGCCTCAACAATACTCAGCAGGCTTTCCTAGTGCCTTTAGTTCTGTTGGGCTG

TTCCCAGCAGGAAATCCCAACCATTTGTGATTAAATGGCCCTCAGGCAACCCAGCA

>Sequence_643

TTCCTGGCAAGACTGTTAGTGATACAGGGATTCG

GGATGAGACAGTAGTTAAAATTCTAATAGTTAAAAACTCATTTAATAGAGTCAACACCACCTGTGGGTTTGGATATGCTT

GGCCCAGAAAGCGGTACTATTAGGAGGTGTGGGGAGGAAGGTGTCACTGTGGGCGTGGCTTTAAGACCCTTATCCTAGCT

GCCTGGAAATCAGTCTTCTGTTTGCCTTTGGAGTAAGATGGAAAATTTTCAACTCTTCCAGTGTCATGTCTGCCTAGATG

CTGCCCTGCTTTTGCATTGATGATAATGGACTGAACCTCTGAACTTGAAAGCCAGCCTCAATCAAATGTTGTCCTTAATA

ACAGTTGCTTTGGTCATGGTGTCTATTCACGGAGGTAAAACCCAAACTAAGTCACCACCTTTCTTTCTATCTGTGTATGA

AAAANATGA

>Sequence_644

GATCAAACGCTACAGGGNAGCAGGCACATGCGGGAC

AGAATGCAGCTGATACCGATGGTGGTGGAACGCAGCGAGCGCGGTGAGCGCTCCCATGACATCTACTCGCGGCTCCTGCG

CGAGCGCATCATCTTTCTCAACGGTGAAGTCGATGACACTGTGTCGGCGCTGGTCTGCGCGCAGCTGCTGTTCCTGGAAT

CGGAAAACCCGGAGAAGCCG

>Sequence_645

ACCATGTGGTTGCTGGGATTTGAACTCAGGAC

CTCTGGAAGAGCTGAGCCATCTCTGCAGCTCCAGACTGTTCTTCTTTGAGGTTGAGGGGATAAATGAGGGCTCAGAAGAA

GTTGGATTTGAATTCACATGCAATTTCCTATTGACCCTGGGTATTGGCCCTCCATGTTGATGGCACACTGCACACACAAT

TAAAAGCACATCCTGAAGAGGGAGACTGAGTTGCCTCCATGTGGTATGGAATGACAGACACGATCCAAGCTGGAACAGCA

GTTCTCAAGGAAATGGTGTCCTGTGAATTACTGAAAACTGCAGTCTGTTTCTCTAGGAAATAATTCTTTTCTTTTCTGGT

GGTTTTATTTTGGATTCTCTCACCTCAGCTCCCCAAAAGCTAGGGTTACTGGCATGCTCCACCATGCCCGACTCCAGATA

GCTTAGCAGCCAATAGTGCTTGCTGCCAGGCCTAAAGACCTGAGTTTGAGCCCACCCCTAACACTGCTAATAATACTCTG

CTATACTTGCAGACAGGAGCCTAGCATAAGTGTCATCAGAGAGTCTTTACCCAGCAACTGATGGAAACAGATGCAGAGAC

CCACAGTCAAATATTTAGGTGGAGCTTGGGGAATTCTGTA

>Sequence_646

GATCTGCCTTCCCAGAGAAGACATAGTTTCAGAAACTCTGGCTCG

GTGTGTGGAAACCTTGTGTTCACAAGAGTATAATTATTTTGTACCCAGGCAGTAAGAAGGGAGTAGACAATGCAATCAGC

ATTCTTTCAAAGTACTCTGTTTAGACACTCAGCCTCCTAGTACTCTGAATACTGAAGTGTTATGGCCTTTGCATTATCTA

AGCATCAAGGAAATTTTATGGGGAGCCAAATTATAGCTTTCTCTCTAGAAGTACAAACACATGATTTATCTGAGAAAAAA

AAGGGGAACCATAGGAGACCTTTCATTTGACAATGATTTTAAATGATAGCATCTTAATTTGTA

>Sequence_647

GATCCTCCAGTAGTACTATGTCCAATTTTCTGAGGAACCACCAGACT

GATTTCCAGACTGGTTGTACAAGCTTGCAATCCCACCAACAATGGAGGAGTCTCTTTCTCCACATCCTCGCCAGCATCTG

CTGTCACCTGAATTTTT

>Sequence_648

GATCTTCTTTAATTTCTTTCATCAGAGACTTGAAGTTTTTATCATACA

>Sequence_649

TAAGAATCACTGTGAGCTGCTATGTGAGTGCT

GGGACCCAACGTGGCCTCTCTAGGATTGTTCTTGACAGGATGGCCTCCAACTCCTCATCTCCTTGCCTCCACCCCCCAAT

TCAAGAATAAAGGGTTCTCTACGGCACCCAGCCTGGTTTGGGGTTTTTGAGACAAGGTCTTGCTATGAGTGGCCTTCAGC

TCAGAATCCTCCTGCCGCAGCTTCCCACTTTGATTTCCAAGTTGCGTTCTATTATACGCTACTTCCACATGCTACCTCCA

GTCCCCTCGAGTGTTGGTCTTGCCCAGTGTTCCCCTATGCTAACTCTACACTAAGGTGAGAGACCACTGT

>Sequence_650

GATCCTCACAGGGGCTCCTCAGATACTCTGGTGTTGCTCTGTGTCATGGA

>Sequence_651

TTCTGCCTCTATTTGTGACTAGGTCTGCTGTTTCCTTGTTTGGGGTCG

TGTTCTGTGTAGGCACATATATTTCATCAATCTTTTTCTATAAGCCCCCTTTTCCTTGGATATATTTAATTAATATGTAG

CCCC

>Sequence_652

GATCAGCAGTGTGGAAGTGTAAGCTGAATATACCCTTTCCTC

CCCAACGTGCTTCTTGGTCATGATGTTTTGTGCAGGAATGCAAACCCTGACTAAGATACCCTGCAAGACTTGAGTCCTAA

GTCCCATCACCACAGCTCCGGAGTAATT

>Sequence_653

TTCCTATACATCCAATAGGGACTTATGGAGGAGGC

AGGGATAGCTGACATGCTGGGAAATGAGTAGCTGCCACATCTGAGAAGGAAGGACAGAGAGACAGCTAACACATTAGAGC

CAGGGAAGAGTAGTCTCAGCTCTACCCCCACCCCACCCAACTGGTCTGGGGTCACAACTGTGGAATCCAGATTTTATTTA

AGTAATAACTCAGGAATATTGGAGGGGAGGTGTTAGCTATTAGTAGGTTTGGAAGTGGCCCAGCCATTGAGGTGTTTAAG

GTGCATTAAAATATAAGGCTGCATTCCTGTGTATTTCATTTTGGGAACTGAGAACATTGGGGCAGGTAGTAAGGACCTCA

TAAGCCATTTTTGGTTTGATTAGAGTAGAGATTAATTGACAACTGCAATATGTCTATATATGTTCAGTCATTTTCTGAAA

TTTGATAATTGAAGTAAGCATCCTTTCTATTCTGAGATGATGTTAAATGATACTTCATCCAGTCGACAGACAACTGCCTC

CAGCTCTGTCAAGATGTTCTTAAGATACGGAAAAAGAAAGTGGAGTCAAATCTAAGATACTAAACATGTTAGTTAAGTTT

CTCTTCTTTATAACAAAATACTTAACAAAAGCNACACATGAAGGAAGGCTTCATTTGGCTACAGAGGTGAAGGATGTTCT

AGCTNTAGACCTTGAGCATCTATCATTTTATTCCCCGCTGCAAGCAAA

>Sequence_654

GATCTGCTCCATGAACAAGACTCCCAGCATGCTAGTCAAAGGACATCCACAAG

ACCACCCAGAACTCTAGACAGGAGGATAGCCAATGACACCCAAATCCACTTTTCTCAAATTCCCCACCCAGTCCCAGACA

ACCTTCAGCATTGTTTGCTATCACTGTACTATTTAATATTTTCTTTGCATCAATTTTGTGCTTCAGAGAAATCCTTATTC

AAGGAAACAATGCAATCCTTGTGGGACTGACTAGAAGCAGAGCTGTGGAATCTAAGTATGTGGGAAACTACAGTGAAAGA

GAACCAAAAAATAAAAAAATAAAAAATCCGCAGACCTTGAACTAAGAAGCTAGCCTTCCTGCCCCAGACCCAAGTCTAAG

GCTTGCTTCTATTATTTAAAGGAGACCCTCTCCTCCTCCAAGACTAGAAAGGAGTCAGAGAAGAGTTATCAGCAGGCAGA

GGTAGTTGACCTGACATGAAGAATTGAGGATAGGATTGAGGATGCCAAAGGACTGGGACAGAAGCCAGTTCTCTTGACAT

AGAATCCCCGGAGAA

>Sequence_655

GATCATAAGTAAACATTGGTTTTATAAGTAAGTGTTAGTATA

>Sequence_656

GATCATCTGTTAAATCCATTTGGTTCATAGCCTCTATT

AGTTTCACTATGTCTCTGTTTAGTTTCTGTTTTGATGACCTGTCCATTAGTGAGAATTAGATGTTGGAGGTGAGGACATT

CATATGATACATGGGATTAGAAATCTGGTGTACCTGGTGAATTGACCCTGCAAAATCAGCATTGGTTATGAAGAGACCAG

GACCCTGACATGAAATCCCTGTTTTACCTGGACAATCAGTGCTGCTCAGCAGGGGCTGA

>Sequence_657

GATCCTGTGATTCTGGGCTCCTGTGATCCTGAGATTCTGGGTGTGTCA

GAATTCTTGGCAGTCAAGCTTCCTCTGAGACCCTGA

>Sequence_659

GATCGAGCCTGAGCCCGACACGTTGGTGACCAACTGGGCCGGGCGTGTCGACA

GGTCGACATCGCCGGAGCCGGAGATGGTGATGCTGGCCTTGCCCGTCGGGGC

>Sequence_660

GATCAAACCTCACAGAATGCTAGGCAACTGTTCTACCACTGAGCAACCTCCCA

TACCTTTTAAGATAA

>Sequence_661

AAAAAGTGTGCAGTCGGTCCTCTGGCAGTGGTGTTTGG

>Sequence_662

GATCCACCCCATAATCAGCTTCCAAACGCTGACACCATTGCATACA

CTAGCAAGATTTTGTTGAAAGGACCCAGATAGAGCTGTCTCTTGTGAGACTATGCCGGGGCCTAGCAAACACAGAAGTGG

ATGCTCACAGTCAGCTAATGGA

>Sequence_663

GATCCTTGCTGAGGAGCCTGATGACGTGAGTGCAATTCACATGGTGGAAGAAA

ACCAACTCCTTCT

>Sequence_664

GATCTGCTCACCTGGGCAAGTCCTCCAGCTGAAAGAAGCCATCGC

TGGT

>Sequence_665

GATCACCAGTTCCGGCGTGTAGCCCAGCCCGCGCAGCTGCTGCAGCGCGGGGTCCAGC

GTCGCC

>Sequence_667

GATCATGGGAAATCCTTTAAAAAGTGATTATTCGCAATATAATGAATTTGCAG

CAAAAGAACACTCAACCCCTAGAGCCGGGCGACCGACTCTAGGAGGTAAGTAGTCTTACTTAACGGAACGCACCCAGGCT

GTTGGCCTGAACGATGACCGCTGGAGCTTTGTTACGACCCACGGTGTTCTTGAAGTGCTCAACCATTTTGACAACCTGGT

TGAAGCTTTCGTTACCGGCGTAACGCTCTTTAACTGCCGGGTTATT

>Sequence_668

GATCAAGCATTATCCAGAATCCGTTAGATGGACCAGGTCACCGGGGCCACCAA

AGGAAGACAGTCGGAGTGTGGGTTTAAAATCCGGGCAGAGGCAGCGCTGCTACCACTGATGTGCCCTTGACAAGTCGCCT

AT

>Sequence_669

TTCTGTGTTTGCTAGGCCCCGGCATAGTCTCA

CAAGAGACAGCCATATCTGAGTCCTTTCAGCAAAATCTTGCAGCTAGCCAGCCAGCCCTCA

>Sequence_670

TTTGCGATTGCAGTATTTTCCGCCGCCCATGC

GCCCTCCTCGCGCCGCGCCTTGCTGTTCGCCCAGGCAGCGCTTGCCGGTGGGCATGAGATTGTGCGGCTGTTTTTTTATC

AGGACGGCGTGTACAGCGCTGCCAATAACATCGTTGCGCCCCAGGATGAGCAGGACATTGCCCGCAAGTGGCGTGAGTTT

GTCAGCCTGCATCAGCTCGACGGCGTGGTGTGCATCGCCGCCGCCTTGCGCCGTGGCGTACTCAATACTGAGGAAGCCAC

GCGTTATCAACGCAGCGCGGTGAACCTCGACGCGCCGTGGGCGTTGTCGGGCCTGGGGCAGTTGCATGACGCCATCCAGG

CTGCCGACCGCCT

>Sequence_671

GAGCAGTTAATAAGGAGAGCTTGGCACCCTGT

GCCTCTGTCTGGTTGACACCCACGGGATTGAGAAATCTGCTAGTTTGCATACTGGTACAATGCTGGAGCTTAACCCCTGG

CCACTGTATGGATGACAGGTGAGAGCCAGATTGAAGGCCAAAGGGGTTGCTTTT

>Sequence_672

GATCCCTGGGGATTTCTTACTAGAATCTAGTTTGAAGAATATATTCTCAGCCT

CAATAAAATTATAGATTATTATGGATTGCTACAGATCCCTTTAGCTCCTTGATTACATTCTCTAGCTCCTCCATTGGGGG

CCCTGT

>Sequence_673

GATCCATCCATTAGCTGACTGTGAGCATCCACTTCTGTGTTTGCTAGGCCCCGG

CATAGTCTCACAAGAGACCGCTACATCTGGGTCCTTTCAATAAAATCTTGCTAGTGTATGCAATGGTGTCAGTGTTTGGA

TGCTGATTATGGGT

>Sequence_674

GATCAAGGGCTGGTCTCCACCATAAACACAGACCTGGCAGGAAGGCCTCCATGG

CTGTTTTCTGGTTGGGATATAGTCTCTCTGATTCAGTTTTTTCATGCACCTCACTGACCATTGCCTCTATGAATCTATGA

GAGTTTTAGGCTCCACTCCAACCAATTGGTACATAATGTTTACACATACTGTCAGGAGCTCTAGAGAGGCAGTCATGTCC

ACTCAAATTCAGTGAGCAGAGGGTGCAAGGTCTGGCCAGAGAGGCTGCCCCTGCCCAGCCCAGACT

>Sequence_675

GGCGGCCGCTCTAGAACTGTGGATCAGGGGGATAAAAATTTGAAAAGTAGAAATCAAAGTGTTGCTACTCACCAATGACA

TAGTAGTATATGTAGATTACCCAAAAAACTCTGAGAGAGACCCCCCCAAGAGCAGATGAACACTTTTATTTAGCTAAGAG

TCTGGAGACAAAATTAACAACAACAAAACAATAGTCCTCCATTATACAAATGATAAATGACCCTAGATAGGAATTAGGGA

AACAACATCTAATCTAATCTAATCTAATCTAATCTAATCTAATCTAATCTAATCTAATCTAATATATAATATTTGATATT

TATAAAATATAAAAATATAATATATCTTTGTGTAACTCACACAAAGAAAGTGAAAGACCTGTAAATAAAGAACTCAAGTC

TCTGAAGAAAGAAGTTGAAAATGATTTTATAAGACAAAAAGGTCTTCCACACTACTGGGTCACTAGATTTAACATAGTAA

AAATGACCATTTAAAAAAAAGCAATCTACAGTTTCAATAAAATTCCCATCAAGTGCCAAAACAATTCTTTCCAGATCCCC

GCACACGATCTATCCAAGTGAATGATCTGCAGCAGTATCTGATGTCCATGCTTTGCCAGAAGATACACGATGTTCCCCTA

AAGTCCCCTAAGGGAATAGTGTTGTGCAGGGTGAGTTCCTCTGGTTTTCCTTCCAGTCTGGGGTCTCTCCTCCTGTGGGC

GG

>Sequence_676

GCACCTCCTCTTCTGTCTCTGAAGTATGAAAA

GGGGGACCCAGGCTGTCACAAACAACCTACCACATAGCTTCACAGGAACTCCATCCCCTCCACTACAAAATAAGGAGTTG

CCTTAATTACCATCACAGTTGTTACTGCTCTCTAGGGTATTAGAGCATTGTGGTCCCTTCCAGCCCAGCTTTTAACCTCA

GCATCCTTTTCAAACACAAAGGGCTATGCCCAGTGGAACCTATGCTCCTCAGCCAGGGAGCACCTGCTGCTATGGCCACT

CACTTCTCACAGCCCCTCCGTGTCCTGTCTCCTGGTCTGCATCCGCTGGAACCGGACAAGAACACTGCATAGCCTGAACT

GGCAGCTTCTCTCCATCTTGCAAAGCACACTCACTTCCCCAGTAAGAAAAACATGGTGCACTTGGCTCTGACTTGGGAAG

CATTAGGGTAGAGTCATTTGAGCTCTGTTCAATTACTCCTATTAAATGCATGAGCGAAACACATAGCTGGTGGGTTAGAT

TCTTGTTAAGCAAAATAAATGGGTTTCAGTTTCAAGTTGGAAGACATCTCTAACCGAA

>Sequence_677

GTACCACAAGTCTCCAATAAGGGAATCGATTG

TGCAACATATAAACTATCAGTAAAAATATTAAAAGCATCATTTACAGTCTGAAAAATGTTCAGTACTGCTGTTAATTCTG

CTAACTGAGAGTCCAGGAGTCTCTATGACTACCTATTGATTATTCATAAGATATCCAGCTCGTCCTTTAGAGGAGCCATC

ATTAAAAATCAAAAGAGCATTATGTAAAGGCTGTAAAGAGGTCATAATAGAAAATATCACCTCATGTACATTTAAAAATT

TTATCACCCTATCTTGAGGATAGTGATTATCTTTAGTTCCTATAAACCCTATTTGAGCAAGCAACCAATCTGTACTGTGC

TGCTTCAGCCAAGTATCTT

**Sequences S2**

Sequences analyzed from the double ChIP library. Vector sequences are deleted.

>Sequence_10

GGGTATAGGGGAAAAATTCCTGAATAGAAAAG

CAATGGCTTGTGCTGTAAGATTGAGAATCAACAAATGGGACCTCATGAAACTGCAAAGCTTCTGCAAGGCAAAAGATACT

GTCAATAAGACAAAAAGGCCACCAACAGATTGGGAAA

>Sequence_12

GGGGGTGTGA

>Sequence_19

GCTTTGGGGTTCACACCCCGATCACTCAGGAT

ACAAGCTACCTTTAATAGATACAATATAGATATAGATATACACACATACATACATATATGTATGTATAGCTAGGNTTTTG

ATCTTTTCTTNATGCTTTTGGCTGGGCTGTCTTTAGTCTTACTATCACAAGATATGTTCAGNCAAACANGGTGTCACATC

TNCCCTAAGACTGATGCTCACGAGTCC

>Sequence_20

CCTTTTCATTCTCCCAGGCAGGCCTTGGCTGG

AGTGCTGGGTTCCTTTTGGAATCCTTTCCTGTAACTCCTCTAGGACTCTTCTCTCCTTAGCTTGCTGTCCAGCGACTTTA

ATTATGTTTCCTTTAGTAGTCTCTAAGCTCTTAGATAGCAAGGCAATGTGTCCTTTTGCCCTGTACCTCAGGCTAGCCTG

ACATTT

>Sequence_28

GATCCGACGCGCCCTGCCCCGGC

>Sequence_31

TTATCCTGAAGCTTTCTTTCTGTGGCAACTGTC

ACTAACCCTTCACATACTAAACAGCAGTGTGTGAGACAGGCTGAATAGTGAAGATGCACCCGATAACAGCACGCATTCCT

TCTGCAGGACACTGGCATTGAATAAAACCACACTAATCAGCTGGCCACGAGCATGATACACAATTTTGGGGGGG

>Sequence_45

TTGTAGGACAAAGGTGGTGTGGAGTTTGGGTA

AACTTAGGTCAACTGTTAAAGGCAGGGTGGCCTCTCTGTAGCTACATCTA

>Sequence_46

TTGTAGTAATCTTTCTTACTAAATTCCTTCCA

AATGTTCCCATTTGAGTCTGTTGTCTGTTTTCTGCTGG

>Sequence_51

GGTTTTATATTACTCCCACATGTATGCCCACA

TATGCACACCCCTCCACACACACACAAAGAAACAAACCAAAAACTGGGAGCCATAAAGGGAACTAAATCTTGGTGAACAG

AAAACTCGAGGCAGGAGAACACAATGACAGTTCAGTAGAGAGCTGAAGTGCAATATT

>Sequence_56

AGTTAATTCTCTCCTTTGTTATATGGATTCTT

GGGGTTGAACTCAGATTCTCANATTTGGTGGCAAGCTCCATTACCCAGAGAGCCGACTCACTGGCCCAAGGTTTGTTTTT

TGAGACATGTGTTACTGGCTGGCCTCAAAACCACTATGTANCCAAAGAGGGACCTTGAGCTTCTTTTGCTCCTCCTGCTC

CTACCTCTTGAGTGCTGGGATGACAGACAGGGAGCACGTNACCACATCTGACTTCTGAGTCTTCTAGTTTTCAAAGA

>Sequence_57

TTTTCTGAGCTCAAGATTACCATAAGGGTATG

GCTAAGTTGCTCCTTGTTATATTGAGGTAAGGAGCATTCTTGAAGGTGAAGGCTCTTTTCAGAGGAAACTGGGAAAGATT

CCATCCTGGCATCACAAGCGTAAGGTCTTCGGCTGGTGGGAAATCCTTCTCAGATCACTGCCACTCACCTGTCTGCTTCA

GCCAAGCTCCAGCTGCAATCAACGGTTGCTAGCTCTCCCCATCCATCTTCCCATTCGCCAGAAGAGATTATTAGCACTCA

GCAACAAAAGCTAAAGCCTCAAGTGTCCCAGAGGGTTGTGTCCTGTGTGTCCTGTGTGACCTGT

>Sequence_68

GACTAAATATTAACAACTCCTTTAATTACACA

AAAGGAAAATCTGTGTCAATGTCTCTTTTAGTATTAAACCTTCAACCTGTCAATTAGTAAATATACTTGTAGGTCATTTT

TCCATTGGGAGGAAGGGGAAGTTCAGTGACAGTAAAAGGGACAGTCAGAGACTTGGTGCTTCTGGTCAAAGAAAGAACAT

GTAATATTGAACAGAAATCAATGTTTGTAATGTCTAGAGAAGCCATTGAAAGCTCACACTCAGTAAAGTTACATATAATC

TTACTGGATAGTACAGTCCCATAAGACTGAGTAAGGAGGATTGCAGAAGTCCTAGTCACCCCAGAGACTGCTACATTTCC

AAAATATTGTCTACCCATCTTGAA

>Sequence_70

CTGTATGATGCCTTCCAACCACCTGCAATTCC

AGTTCCTGGAGGCCTTATGCCATTCCTAACCTCTAGGCTCCTGAATGCATATGGTGTATATGCACTCAGGCACATATGTG

CACATAAGATAAATATTGTCTTTAAATCTTCAAGAGTACCTTAGTGCAAAGACTAAGAATGTAGACACACTTTGGGTCCA

TGTGAGCACACAGTGTTGAGTGAAGAGTGGAGAAATTCTGAACTTAACTTTGTCTTCTTATCTAATGGGGAAGATGTAGC

TACATGATATGACTAATGGGCCTCGAAGTCTCAATTATATATCATGATTTACAGTGAAAGTTGCCGTCAGTATAGAGTAG

TAGTCTAGGAGGCTTGGATGAAGTCTTGAAGACAGGTGTAAGCGGGAGTAGTAGTGGGGTTCAGGGTGGAGGAAGGGACT

TGGGGCGCTCTCCTGACCCCAAGCACTTNCACAGGCAGGTTGAGATC

>Sequence_77

ATGATGGTGAGTGGTCTTGGTTTCTGATAGTA

AGATTCTTATGTTTACCTTTCACCATCTGGTAATCTCTCGAGTTAGTTGATGTAGTTATCTCTGGTTAGAAATTGTTCCT

CTCATGATTCTGTTAGTCTCTATCAGCAGACCTTGGAGACTATCTCTCTCCTCTGAGTTTCAGTGGTCAGAGCACTCTCT

GCAGGCAAGCTCTCCTCTTACAGGGAAGGTGCACAGATATCTGCCATTTGGACTTCCCTCCTGGCCGAAGATGAAGGCCC

AAAACAGGACCTGTCCCAGAAGCTGTGTTGCTTCAGCCTGTCACAGAAGCTGTTAGCTTCTGTAGTCCACACTCTCACCT

GTGCAGAATACTCTTGGCAGAGTTCTGGAACCAAGATGTCTGCAGCTTTTGCTCAGGGCAAAGCCCTCCCGGGCAGGGTG

GACACCTATCCTTTGGCCAGGCAGGTGCCCNAGATGTCTGTGGGCC

>Sequence_78

TAAAAGAAAACTGAAGATGGATAGGCAGTGTA

ACTTGTTGTGCAGCAATCATATATAGCCTGTGTAGGGCCCTAGAGAGCAACCTTCTGTGCTGCAAAACATACAAATAAAT

ACATGAAAAAATAAAGGGAAATTTCTGTTACTTTCCTCCTATCTGGAACAACTTTTAAAAACTGAACAAAACACACAAAG

CAGTAACAGTTCTCAGTTATTGGGCATCAGGTCATCTTGGAGATGGTAAGTGGGCGAGGGAGCCTATCTTAATTATTTTC

ATGTTGCCATAACAAAACACCATGATGTAGGCAACTTATAGAAGAAAGCTTTTAGTTTGTATCTCACAGTTCCAGAGGGC

TGGAGTCCATGACCAAAAATGGTGGAGCATGGC

>Sequence_86

TGGTTACTGAGACTAACAACCTGACTTCAATC

CCCAAGAACTACATGGTTGGAGGAAGAGAATCAACTCCCATGGTTGTCCACCGGCCCCCACATGTGTGCTGTGGTACACT

CTCATCCATGTAATACACATATGAATAAATAAACAAGTAGATAATATAATTAAAAACAACTTTAATGACAATCCCTCACT

ACCTCCAGCCATAAAAATCATTGTTTAAATTTATCTGAGGGACTTTTTCTTTGCTTGAATTAGGTCCATGTGGTATAATC

TATGGATAAGTTTTAATATGTAGCAG

>Sequence_98

CTGATTATCCTTCATGGAAGATACCTGGGCTG

GGCAAGAGCTATCGGCCACTAGGCCAGCCTAGACCGCCCATTGGTGCCTTCAAATTGGCCTCGAACGGGCTCGGAAACGG

CGTCACGGCCACCGAGGCCGTAATAAGGGCTGCTCAAAAAAAATCTAAGCCGCTCTGCATTTGAGGTCAACAGAAAAGCT

AGCCGGCCTAGCGGCCGGATAAGCGATTTTTTTTACGACGGCTCAAGCCAGGAGACATCGTAGCCTTT

>Sequence_100

CAGGGCTACACAGAGGAAACCCTGTACTGAAA

AGCCAAAAAACAAACAAAACGAAACAAAACAAAAAGACTAGCTTAAAATAGTAGCTTTTTAACACAGGATAAAGAGACTG

AAAACCAATAATCTCAACACAGCTGTGGCTACATAGCAAG

>Sequence_105

TACAGTTGGCTTAGGCTAAGGACACCTGGGCA

GAGACCACTGATTTCCATCCA

>Sequence_113

CCGGGGGGGGGGGGGGGTCTGGTTATTTGATA

TTGTTCTCCTATGGGGTTGAAAATTTTTTGGCTTNTTAAGTTCTTCCCATAACTCCTCCACTG

>Sequence_115

GATCACAGCCTCCTGCACGCCGGAACCTGCACCGTACG

CCAGTCGCGTGGCGGTTGAGCGATAGTCTATTACTGCCGACGAACCCGCACGGCCCGACTGTTGCCCGCGACAAGCTGAT

AGCTTCCAAGCGCAGCGCGTCGAATGCGAATTTGTATTCCCTCCTGATTGCGAAAACGCACG

>Sequence_116

CATCAGCAGGTACTCTTTTTCTCTTGTTTGGT

CGATTTATGACTTTGGCCTGGAGCTAGCTAATTGGGCCAGTATGGATAGGCAGTGAGCCTTAAGGTTCTGCCTCCCAGTG

CTAGCATGACAAGTATATGCCACAGTGCCCAGCTTTTTGACATGGGCTCTGGTCATCACACAAAGTCCTGGTGCCTTCCT

TGAAAGTACTGTACATGAAAGCTATCTCCTGACCTAGATTCAGAATTCTTAAAATGAAATTTCAGACCTTAATTAAGCAT

AAGGATGGATTGATTGATTGATGCAAAGCAAGGTGGCAACACCATTTAATAACAATAATAATTAACATGTAAGTGTTTAT

AATCGATATCGAATATCTTTCAAAGAGCTTTCTATGTGGTGAGTCATTAAATCTTCAAATGGCTCTGTTTGTTTGTCTGT

TTTACTACAGTGAGGATGGAACTCGCAGCGTCATGCACATTCAGAAAAGTGCCACCACTGAGCTGCTTACAGCTCAAGAC

ACAGCTCCTTGAAACATGTATTCTGCAGATGAAATTTACAGAGGAGGACCATT

>Sequence_138

GATCGAGAAATCGACAAATGGGACCTAA

TGAAACTCCAAAGTTTTTGCAAGGCAAAAGACACCGTCAATAAGACAAAAAGACCACCAACAGTATTGGGAAA

>Sequence_140

ACTAACTCGGGTTCCAGGCGCATGCTCATATC

CTGCGCCTGCTCTTGCGCCAGCAACAACTGATTGCGCAGGGCCACCAGATCCGAATCACCTAAACCGTCATTTTTCTGCA

AACGCTCCTGCACCGTTTGCAGCGTGTCCCGCAATTCATTCAAAGATTCACTGGCTTGGGCAGGCGTCCAGGCCACCGTC

TCAGCCGCAGTGGAAACCGTCACGATGCTCAGCGCAAGCACCAGCAGCCACAAGGACAAAAAAGCACACAGGCGCGAACG

GCGAAAGGCGTGCATCAAGAAAACTCCGGCTAAACAATCAGGCCCCTCATGACAATGTCAGGGCAGCAGAAACATGCACA

GAATACCGAAAGAATCACTCTCTTGGCACAAAACAAAAGGGACAGGCGAAAAATCCCGCTCCCCCAACCGGCTCAGAGCG

CCTTGCGCTGCATGCCCTACCCAGTCAGCCACGGTCATTTCGAGTCGCCCAGGCAGCAAGCCAGTCAACACACAAACCCA

NGCTTAAAAAAAGCCCTTACAAGCCAGGCACAAAAAAGAGAAAACCCTAAAAGAATA

>Sequence_146

GATCCCTTCTCTTGAGTGTGGGTTAGATTTAGTAGATTATTTCTAATACATAGTAAA

TTGCAGAAGGGTTGTTGTGCAGTTTTAAACTGAGGGATTTTGCTTTGTTTTGTATTGTTTTAAGACTGTGACTTCTCCTT

TTGGTCCTTTTTCAGCCCCAATCTGTTACCATTTCCTTCTTCATCATCTA

>Sequence_155

GATCTTCTGTACCCAATGTGAAAAGCAGGGCTAGGAG

GTCCAAGACAGACAGGTCCCTGCAACTCAGTGGCCAGCAAGTCAGAGACTCTGTTCAAAGTGATAAAGAAGACACTTGAT

GCAGTCCTCTGTCTTCCACACCCATGTGTACTCATGTACATGCACATGTAACCAAACATGGACATGCTTATGCAAAACAA

ATGTACAGGCATATACAACACACACACATCACATCCCAAAATGCAAGTTACCAATGGAATTTTCAATTGAACAAATGGAT

TGTTACATTTCTTTATCTGTCCTTTCATTTTATAAATAATTTTTTGCACTAAACTACATATTATTTATTCCAGCCCTTCT

CTGTGAGCCGTCCATGTTGCAGCTGGCAGTAAGAACTGGATAAGTGTCAAAGTTAATGATTCAAGACA

>Sequence_160

ACTTTTACCCAGCTCTCAGGGTTCACTTTAGA

AATCTTACCCGGTTCGGGCGTGGTGGCGCATGCAGGCGGATTTCTGAGTTCGAGGCCAGCCTGGTCTACAAAGTGAGTTC

CAGGACAGTCAGAGTTACATAGAGAAACCCTGTCTTGAAACAACAACAAAACAAACAAATTCATAGTTAAGACTTGGTTT

AAGATTATGTAACTAAAGCTGGCCATGGTGGCCTTTAGTCCCAGCACTCGGGAGGCAGAGGTAGGCAG

>Sequence_220

GATCTGACTGTTGACTAACATTCTTCCAGGATGGGCC

TGTCAGGGTCCCTGGACATTGAGTCACCCAACTGTAGCTGCTCCTTGGGTGCAAATATTCCTGAACCTCTCAACCTGTGA

GTCATGAATCATTTGGCAAAGTTTTATCTCTAAAGTATTTACATTACGAGTTATAACAGTAGCAAATGACAGTTATGAAG

TAGAAGGAAGATAATGTTATGGTTGGGGGGGGTCACCACAACATGAGGAACTGTATTGCGGGGTCACACATTAGGGAGGT

TGAGAACTGCTGACCTATGGATTAGGAGGGAATAAGAGCCTCCTTGACTGAGCGTCCTGTTTTCTATGTGACTTCGGGCT

GTGCCCCAGGGTAAGGCTTTCCTGGGGCCAGCAAGTTA

>Sequence_221

GATCCCTTTGTGGAATCCTGAGGTCTCAGGGTGTCTGGGA

GCTCAGCGTTGAAGACATATTACTTGAAAGCCATTGTCCCAATTTCACAGCTGTTTGTCCTTTTAGTTTTCATTATAAAT

CTGATATAGAAAATAATAAAAACAATGTCATTTGGTTAATTTCTAGTCCTTTTTCCAATAAATATCTGCTCTAAACTAAT

GACAGTGGCAATGACTTTCCTGAGCCCATACCCTCCCCTTACTGACTCCAGTCTCTCCAGGCTCTCGACAGAGGCTGTGC

ATGTGTGCACCGTTTGCTCAGTACCAGATGTTGCTGTGCCTGAGCACTGGTTTGGAGAGTGCTGTAGTCCTGACTATGAT

CTCCAGTGCTAGCTAACTCACCGTCAGTTGATGAGATGGGTCATTTAAATGTACAGCATAATTAGAGTTTGCCTCTATCT

GTTGGGGTATACTTACAGGGAATTCCTTGTCCCTCTCTCTTCCAGGGACATCTTTATGTACGTATATGTATACTTTCCCA

TTAGTGAAAGGTCTCTGCCTAACTGAACAATGGGTTGGCTCTGCTGTCAATCTTG

>Sequence_224

GATCAGGCGTGACGCGCATCTGGTCGGTCTGAACCAGAGAGA

TGGACTCCACCCCGCCTGCGACCGCCACCTGCATGT

>Sequence_225

CTTGGAGAACCCATGCAAGAACACATAACGGC

TACCTATTCTGGCCGAAAGCAAGCTGCGATAGCCGCCGCTTTTGCCACCACCAGGGCGAGCTACCCGCTTCTTGTATAAG

GCGCCCCCCAAGTCCACATC

>Sequence_228

GATCTGATACAAGAATTCAGAAATATAAAACAAAATAACTAT

AAAAGTTAGGAGGCATCTGAACAGCATTTCCTTAGAAACAGCTGCTAACTCTGAACCATTCCCGTGTGGATTCCTATAGA

CGCTGGGGTGAAATGGGTATCTTCCCCCTCCCTTTTCTGGGTCCCTGGACTTTCTTCCAAAAGCGGTAACATATGGCCAA

ACATCCCAGGAGTCTCTATATTACATACGCTCCACTTAACAAGCCAATCAGATGGGAGAGGGCAGGGAATTATAGAGTGA

GGGGTCCAAAGGTAGCGGCTGGTTTACTGAGAACTTGCCCTTTCCAAAATGTGGAAATCGTAGTGCTTCTTGCTCATTCT

CAGCTTAAACTTGTTCACTGAA

>Sequence_229

GATCCGATGCCCTCTTCTGGTGTGTCTTAAGACAGCTACAGG

GTACTCATATACATAAGATAAATAAATAAATGTCTTAAAAAAAAAAAAAAGAGCCAGGTGTGGTGGCACACACCTTTAAT

CCTAGCACTCGGGAGGCAGAGGCAGGCGGATTTCTGAGTTCGAGGCCAGCCT

>Sequence_230

GATCTCATGGAGGCATTTCCTCAACTGAAGCTCCTTTCTCTG

TGGTAACTCCAGCTGTGTCAAGTTGACACAAAACTAGCCAGTACAATTGACCCCTTGTCAACTTGACACACAAACACATC

ACTAGTAAGCCTAAACCCTTACATTCTTTTTTTTTTTTAATAATTTATTTATTGATTATATGTAAGTACACTGTAGCTGT

CTTCAGACACCAGAAGAGGGCATCA

>Sequence_231

GATCAAGGAACTTCACATAAAACCAGAGACACTGAAACTTAT

AGAGGAGAAAGTGGGGAAAAGCCTTGAAGATATGGGCACAGGGGAAAAATTCCTGAACAGAACAGCAATGGCTTGTGCTG

TAAGATCGAGAATTGACAAATGGGACCTAATGAAACTCCAAAGTTTCTGCAAGGCAAAAGACACTGTCTATAAGACAAAA

AGACCACCAACAGACTGGGAAA

>Sequence_233

GATCAGCAAGGCCAGTATGGCGGCCACCACGG

ATAGCTCGACGGTGGCAGGCAGACGTTCTTTGAGTAGTGTGGAGACAGGCTGGGCTTGCCTTAGGGACAGACCAAACTCG

CCTTTTACGGCCTGGCCCACGAAGCGTCCGAATTGAACGGGAGCAGGTTGGTCCAGCCCCAGGCTGGCGCGCAACTCCAC

GC

>Sequence_234

GATCACGAAATGTATCAAAAATGCTAAAAGT

ACAGAAGTACAGTGGGTTACTTTTGTTTTTAACCTTATCCCCGA

>Sequence_235

CCAGGCATTGAGAAAACAAGTACACGTCAGAA

ATGGCAGCCAAGAGACATTCAGAGACATTATAATGTCTCCTAG

>Sequence_237

CTCTGCATCCAGGCCTGGCTTCCACAGTATCC

TTCTACTACGGTGGGACCTCTGAACGC

>Sequence_239

GATCAGAGGACAACTAGGGGAAGTTGATTCTCTC

CTTCTGCCTTGTAGGAAGGTCCTAGGGACCAAACTCA

>Sequence_242

TGACCCATCCATCCTGTAGAGTGAAC

AGCATTGCAGGTCTGGGTGGGGGGGGGATCCCCCGGGCTGCANG

>Sequence_243

GATCCCCGCGCCACGGTTCCGGCCATCCTGA

AGCTGGTCGATTCCGACGCGCCGCCGCTGCGTCT

>Sequence_245

GATCTGACGCCCTCTTCTGGGGTGTCTGAAGA

CAGCTACAGTGTACTTACACATAATAAATAAATAAATCTTTTTAAAAAAACAAATAAAACACCTTTAAAAAAACTCTCAG

CTTCTCCTGCACCATGCCTACCTGGAGGCTGCCATG

>Sequence_247

GATCCATGAGGGTATGTCCCCACCATGCGGA

CACAGCCGTGGGGAATTATACTGCAAGGAGCAAGCACGGACAAATCTAGCCTCTCAAGGTCTCATCAGGAGATAGCAGAC

TGAGGTGTCGGGTGCTGAATTTTCCTGGGGAAAAGGTCACTCTTCTCGGAGAATCCTTAAGCTCCAGGTTCCTGTAATAA

GTAACTGAATTATTTGCATAGCAGCATGTGGGCACT

>Sequence_268

GATCAGGGGCTCCGACTGTTCCACCAGG

ATGGGTGCAAAGGGCCCCACAAGCCAGGGCAGTGGCATGGCCCGCAGGACCAAATCCAGGAACTCATCCACACAGGCATG

TGCCCGGGCCACACTGCCCCGGCCCTCAGCCAGAGCAGTGGGTGCCACATCACCAAGGCAGTGTGCATCAGCAAAGGTGG

CCTGTAGAGCATCCACGCTGCGCTGCACCTCAGCCACCTTGGCCTGGGCACTAGGTGGCAGGCCCCGAACACAGCCTGCC

AGGGCATCCACTGCATTCTGCAGCTCCAGGGTCAAACTTCGAGACAGAGCCAGTGTCTCCAGCTCCACCTGCAAGGGCAG

GCCTCAGTTTCCTTATACCAACAACAAGAGCCTTGGCCTATCTTAGTTTTCTCATACATTTAATGGAAGCTTTGGCCTGC

CTTAGTTTCCCCATGAAATCTAATGTGAGGGCCTTGGCTTGCTTCAGTTTGCCATGAGAACACAGTAATTTGACCTAATT

TCTCACATGGACACCTAACAGGATTGACCTGTTTNCCCTATGACACACAGTGGGATAGAGGGAATGACCTGGTGAGAGTA

CTGGAGCCTGGGCTTGCAACACACAAGGTCGCTGTCCCAGGTGCAGGG

>Sequence_270

GATCTGGCGCTTCATGGCCCACAGGACCA

CACCACCCAGACACATTACCGTCCGGCCTGCACTCTTCGACACCCAGTGCGTGCACACAAAAACGTATGCGTTAGCTTGA

AACGGAAGTCCTGGCATCCCACGCTTGTGACGCGGCCAGCAACAAAGTTTTTAGGCGCAAAAAACCGGGCGGATGGCCCG

GTTAACAGTGCCTCGCTACAATTTGCTCGGATATGGAGCAAAAACACCTAGTTACTTTACCTTGACTGCACAAAGCGAGC

AACTGTGATTGTTGGTAAAGTGCAAAAAAACAGGCTTAAAGCTTACATACGTAAAACGATATACTGTCTACCCATGACGA

GCTGTCCGGGTATTTTATGGCATCGCCCATCCCACAATTAAAACAATTCCTGTATAGCCACTATTTTTTCGGTGGGCTGC

GCCAGTCCATCGGCGTGTTGTTGCCGGTCATTATTTTGGGCCGTTTCTTCGGTCACTATGATATTGGCGTCATTGCCTCC

ATGGGGGCCACCTGTGTGGCCATCATTGACCAGCCGGGCGGNCCGCGCCGCTACCGCAACAATGAAATGCTCGGTGGCAT

TGCACTGGGCACGGTCACAGTCGG

>Sequence_271

GATCAAAGAAGATGTGAACGGTTCTAGCA

CCAGCTGACAGGCCAGTGTGGATGAGGGAAACCTCATGGAGCCCCACCTCTCTATGGAAAGCTACAAGCAAGCAATGACG

CCTGAGAGAGGAAGGGTTAGTTTCAGGGTTGAGCCCCCTTGAAATCTCTGCAAGTTCAAGGCCAACCTAGCTGCATAGTG

AAACCATGCAAAGAAAAGCCATGAATTTGGGGGTTGGGGGAAACATGGGTTGAGCTGGTGGGAGAGAGGGGAAAATGTAA

ATACAGCACACATATATGAAATTTAAATTTAACCAGACCAAAAAGTATCTAGGTGGTAGGAAACTAGAGGCAGAAAGACA

AACATTTTATTTGGAAAAACAAAAATGAAAGCCAGCAATAGTGAGATGGTTCAACAATTAAAGGCGCTTGCCACCAGTCC

TAAAGGTCTGAGTT

>Sequence_272

GATCAAGAGTTGTGTCTCTGTAAAGTAGAGT

CTTAGGCAGGGTATGCAGAGTTATTCATCCTTCATTTCCTAAAGAAAAAGCACTTACAGTTAAGGCCGGTTCTATTGCAC

AGTCACGTAATTGAGAACATCTGTACAAGACTTTATTGCTTTAACAGAGCTTATCTCTAAAACTGATTTTCCTGTGTGCC

AAGTACTGTAGTGAATGTTACCATCACTATATCAACAGGAACAGTGGCTAAGAAGTTCCAAAAACTTGATTTCAAACCAA

GGTTTGATATCA

>Sequence_273

GATCGCCTCGTCGTCACGGGCGCCGCCG

>Sequence_274

GATCCCTCTCTCTCTCTCTCTCACTCTCT

CTCTCTCTCTCTCTAGCTACCTCCCTCCCTAGCTACCTATCTGTTTATTTTTATTTTGAGGTTGGGTCTGATTCTGAAGC

CCATACCTCAGACTCGTGCT

>Sequence_276

GATCCCCCTGGTACCGTTGTTAATGCTGTGAA

CTAGACCTGGGTCCCGTGGGATGACAAGTTTTCTTAACTTCTGAACCATCTCTCCA

>Sequence_278

GATCCTGCCAGACGCTTGGCTGGTACTTCACG

TCGACGCCGTAGGCCGACGTGTCCTGAGAGATGACGAGGATCTCCTTGACGCCTGCCTTGACCAGCCGCTCGGCCTCGCG

CAGCACGTCGGCAGCCGGGCGCGAGACCAGATCGCCGCGTAAGGCGGGAATGATGCAGAACGAGCATCGGTTGTTGCAGC

CTTCCGAAATCTTCAGATAGGCGTAGTGGCGTGGCGTCAGCTTCACGCCCTGCGGCG

>Sequence_280

CCTGGGTCGCCCTGGCAACCCTGGTGGCCATG

GAGGTGGTGCTGGGCATCGACAACCT

>Sequence_285

GATCCAGACAAAGGGAAAAGACTGAAGTGGTGTGAGGATG

CGGGGAGGAAGAAGTCCGTGCTTGTGTACCAGTAACATCAGCCAGAGAGGTCACCCCAGCCAGGTGT

>Sequence_287

GATCTTTTATTTGCTTGATTAGAGTTACAACAAG

GTAATGTATATTATTTGTGACTATGGTTAAGGGTGTTCTTTCCCTAATTTCTATCTCTGCCCATTTATCATTTACATAAA

AGTGAACTACTGATTTCTTTGAGTTTATTTTCTCTCTACATACTTTACTAAATTTGC

>Sequence_292

GATCAGCTTGGGCTATATAGCAAGACAGGCTTTAAAGACA

AAGTAGCAAGATTTCGTTGGCTCTGGAGCTGAAGGCAGTCCCAGATGGGTGGGCAAGGGACACAGCTGTATTCTTGGGAC

AGCTGGGGACACAGGCTGGATGAGGATGCAGGTTCTGAACTAAGGTCTCCAGTGACCTTTCTTCTCATCAGAATCTCAGA

TTACCCCGAGGTGACCTAGACATGAGTCCACTCTCACAGTCACTTCCATAGAGTCTGGAGCAGGCCACCAAGGCACACCA

CGATAGCAGTTTGGTCTAGGAAAGTCAAGAGGGCTCACGAGGTTGAGGCAGCTAGACAAACAGCTCAAGGCCAAGAGCAA

AGGGAGATAGCTGGTGGGAATACCCTGCCAGTGATACCTGCCACACCAGGCAGCCAGAGACCCAGTTCAACCAGGGAAAT

ACAGTTCACAAAGTATTTTTACCCATGGAAATAAATCAGAAAACAAAACTAGC

>Sequence_301

GATCTCAGCTGATCTCTCCTCTCAGACATTTCCAAATGCTGGCAC

CAGCATCCCGAGGGGTGGCAGGATGGCTTT

>Sequence_321

GATCTGAGATGATCAGACTCCCTGGGCTAAG

AGCCTCCAAGTCTCCTGGCACCACGTTCCATAAGATTCTCTGATTCGTAGGCCTGGTGGAA

>Sequence_328

ATCTCATTATTTTGTGGTGTTTTGTTGTTTGT

TCTCAGAACTGTTCTATTTGAACAACTCAAGAGGAAGCAATGGCAAGACTTCTAAACTTGTTTGACAATAAATGAAGTAG

TTTATCCCAAAGTACACAGGAAGATAATAGTAGAAGACAGTTTATGACTCCTGACCTCTTCTT

>Sequence_336

GATCCAAGGCATAGAAACATTCATTTGCCCAG

TCATCAACATATGAAGCAAGGATACATGAGCCATTACAAAATTTTACAAGAGACACAGGGTTGTTTTTAGAACGTATGCC

CAGGGGGATTCCTTTTTGACTTAGCACTTCAGCTCTCAAAGCTGGATAATGGACGTCGAATCTCTCTTCTGTTTCTGCTT

CCTGCTGACATTAGGACGCTGCAAAATGTCTCAGGAAGGCCTAAATGCTAGGCAAAGGCTGGGCAATGGAGCACTTATCA

AAGACATCTAATTGGCTAATCTAGATGAGAGACCAGGAGCAATCACATCGGCAACATCAGCTTTCAGCAAGTCCAGGGTT

GAGCATCGGCACAGGGGACCCCTGGGCGGAGTCTGTTGTGTGTGGGGGGTAATCTGCTGAGACAAGTATAGACTAGGAAC

AGAAATTAAAATTGTCTAACAATTTGTCAAATGTAAAAAATCCTAATACCAAGGAAAAACTTCATTTTCAAGAAAAGACA

GGTGTACTTATCTGGAGTCCATTTGATCTGTGACAGCCATACCACCCTGAACGCGCCCGATCTCGTCTGTCCTGGTTTCT

AATGACTTTGAAACGTGAGCCCTCAAGAGAAATAAATGATAGGAATCACTGATAGATTGCTCCACTTGTAACTATGAAAT

GACTGGTGATGGGATGATGAGGACCCAG

>Sequence_337

CTTTAAAGGTGGGGAGAGTTTTCTCTCGTTCTTTCTGAGAAGCCCGCCG

CTTGTAAGTTGAACCCTTCAGGTCATATTTCATATGCATTTTGACTGACCGAGGCAAGAGATTGTTCATCACCACAATTC

GTATGTTCTTGC

>Sequence_338

CCTCGAAGTAGATAATGGTTTTCGCGCCAAT

ACGCCCGAGCTGTTTGGCATCACCAACACCCGCGATACCCACCACCAGCGTGGA

>Sequence_339

GATCGCTCCGGGGCCTTTTCGTTTGTCTGA

GGTCGAACCTTAGGCCGCTTCGGCGGTCG

>Sequence_341

CAGCTCTAGCCCAGAACAGGTGTGC

AAAGTGGCACTTAG

>Sequence_342

GATCCGGCATCCAGTCCCGCAGCCTGCGCCTG

ACGCAGCAGCCAGGCCCATTCCTCGAGGTGGCCCGGTTCTATGACGTGACCTGTCTGCGGGTCAGCCGCCCAATCCTCAG

TAAAATATTCGC

>Sequence_343

GATCGCTTCGGCGAGCCCGAAGCCCAGGAC

ATCAACCCCGACGAAA

>Sequence_344

GATCCTCCTGCCCCATGTCTGCCTCCATGCTACCATT

CTTTCCATCCATGATGATAATGAACTAAACCTCTGAAACTTTAAGCCACCCTCAATTAAAATTTTTCCTTTATAAGAGTT

GCCTTGGTCATGGTGTCTCTTCACGCCAATAAAACGCTAATTAAGACATATCCTTTTAAAAATAACACTGCACCAAGTGA

GCTATTCTCTCTCATCCCTGCAACTATTTTTTAGTTCCTTTGAGACAGAATTTTGCTATAACATTTTGTCT

>Sequence_345

AATGGCTATCTCATAAGATATTCCATTGCAGCTTCTTT

TTCAATTGCTTTTACTGTGAAATGCCCTTGTGGAGAGTTCTGGGGACGATTTCACACTTATGGTATTGATTTGCTAAACC

TGATGATAGGTTTATATTTAAGATACAGTATTGCATATGAGCAGAGTGAGAAACTTATGAAAAACAGAGCTCAGGCTTCC

ATGATGACATGGTGGAAACTATTGTGTGTGTGTGTGTGTGTGTGTGTGTGTGTGTGTGTGTGTGTTTAAGACTACAATAA

GAATTCCTCATTATCTTTACATTTATTTCAGAAATTCAGCATATTAAAATACTACTGAAAATAACAGTTTGTCCTGCTGT

CCCAACATTGCTCAACTCTAATGAAGTACAGGTTACTAGTGAATGTATAAAGCCATTATAAATATCATACAACATAATGT

TGCATAATCTAGCATGTTATAATATTCTAATGTTGTCTTATATTCCCACTT

>Sequence_346

GATCAGACGCTTGCGCTCCGCCACGCGCCAA

TGCGAAACTTCCCTGGAAAGTTCCGGCGGTGGCCCGTCAGGCCGGTCTGGTATCGGATTGGAGCCCTCTCCAGCCATCGC

CTCTCCTCACGCCTTCTCGCTGACTTTTGGTTCAGGCCCTGAAGCGTGTCGCGATCCTTCAGATCGCTCCCTACGCTTTA

AGCTCTTGTTTGCACGCATGTCGTTTGCCCGAAACCGGTTCCCACTTTCGGGCGGCATGCCTTAGCTCAGGACCTTGGCC

TTTCCTTCTTC

>Sequence_348

GATCGACGAGCGTGACCAGTTGTTCTATAGC

CTGTCGCGCAACATGCGGATTCCGCAGAACTACGTGCTGTACGACAAAGGCGTCGGCTCCCTCGACTCCAAGCCGGAGCT

CAGCTGGAACCAGGAACTGGGCTGGCGCTACACCGGCGAAGACAGCACCCTCGCCGCCACCTTGTTCTACATGCAGTTCA

AGAATCGCCAACTCACCTCCCTGGACAGCAATGGTGATTCCGCCGATATCAACGCCGGCACCGTCATCAACAAAGGCCTG

GAACTGGAGTGGAGCGGCAAGCTGCCGCACAACTTCAACTACTACACCTCCTACACCTACACCAGCTCCAAGCAGCAGGA

TGACCTGACGGTGTACAGCGCCGGCAAGCC

>Sequence_349

GATCTAATGCCTTTGGCCTCCAAGGTTACT

GTATGCATGTGTACACATGCACATACAGACACACAAATTAAAGTAGATAAAATATAAACAGAGATGACTTGTTGGAGCTA

GCACTACAGCGCTGGGGCAGGTCGGAGCCCAACATACACAGGGCTCTGCCTTTAGGCCCAGTGGCCATGCAAACAAAAAA

AAACATGTTGAATGCCCTCCTTAGTTTGCTGGCTCGGGAATTCTGGGTAAGGTCGCAGGGAACAGAACTGTAGGAGGTTC

CATTCTTAGGTCTGCAGACTTGTATCCTCGCTG

>Sequence_350

GATCAGGATGTCAGACTCTGAGAGTTGA

ACACTCTCAGATGAATTGGGCATTGTCCCAAGCTGGGCATCCTA

>Sequence_352

GGCTCGGGGCTCTCACTGATCCTGATACCCA

ACCAGGGNACACTCAGATCGCCTTTGCGAGTAGCCCTGTTCTGGGCCGTCTTTGGCCACAGTGGGCGACAACTGCATTTG

GCAAGGCGAGTCCGTGCTGCAAATGTCACTCCAGAAGCCTTTGGCCAGGTGCTCTGCCACCTCTCGCTCCACACTACTCC

GATCCTCAGATGCAAGGCTGCTGCTGTCTTCTTTAGGGCCCATGCTAGACTTCAGATATAAATCCTGATTTTCCGATGGC

TTCTCGTCCTGGGCATCTGGCCTCGACAGAGGCTTTTCTGTCTTCACGGTTTTACTCTGCACCCCAGCAAAGGCCACGTC

ACCTGACTGCTCATATGTGTGTAAGGCTGACAAGGAGTAGAGCCCGTGAGGTCCTGCAGAATCCTGAGGCAAGGGTGTCC

CTTGGGGCTGCTCTGCAGGGCACTGAGGGGAG

>Sequence_353

GATCAGATACTGTGGATTCCTTTTGCACAAGGACCCCTAAAAGCCCC

ATGTGTCTGCGTGAGCCCTTGGAGACAGCTGGAAGAAACCTGCTAATATCTGCCATCTGGAGGGGGCAGGACAGGAAGAG

TGGGAGATGGTGGTGTCTCCTTTATATTCCTGAGCCACAGTATAAGCATGACCAAAAAAGGCCACTTGTAAAAGAGACAA

GGGGGCTAGAAAGATGGCTATGGTAGTTAAGAGAATGGCTATGGTAGTTAAGGTTGCGGGCTGGAGAGATATCTCAATGG

TTAAGAGCACTGACTGCTCTTCCAGAGGTCCTGAGTTCAATTCCCAGCAACCACATGGTGGCTCACAACCACCTGTAATG

GGATCTGATGCCCTCTTCCGGTGTGTCTAAAGACAGCTACAGTGTACTGTACATATACATAAAATAAATAAAAATCTTTA

AAAAAAAAAAAGCACTGGTTGCTCATAGGTAAGACTAGGGTTTGGTTCCCAGCATCCACATGGCAGCCCACAACAGTCTA

GTTCCACAGGCTCTGATATCCTCTTCTTCTGGCCTCCATCACATGGTGTATATACATACACTCAGGGCGTATTAAAATTA

ATAAAAATTAAAAAGATTTTTTTAAAAGTAAAAATNAATAAATCTTAAAAGAACAAAACAAAANCTCAGCATTTGAACCA

GGTTGTGCTGGCTCATGCCTTTAATCC

>Sequence_354

GATCCGACACGGTCGTGGAGGTTTCCAT

AAAAATAGTGCGACCGCGACGAACCGCTTCACCATAAGGGGTATCCGGAATGCTGCTCTCTGCAGGGGGCAGGTAATACC

CATCTGCCCCCACCGGCAGCCCGGCGGTCACCGCTAACTTATCCTTGCTTTGGGTCTTGC

>Sequence_357

GATCTAGGTCAGCGATAGAGACCCTGAGCTTTA

TCTCCAACAACTCAAGGTGGTGAGGAAAGGGAAAGCCATCTGGAAAGCAAAAGCATTTTCTGAAAATAGATAAAGGCTCA

ACCGAGAATATATTTATAAAGTGGCATTTCAAAAGAACAAGCATAAGAAAAACTGTCAACAGAGAAGGAGAAATTACTGC

AGTAAAGCAAGATAAACCTTTAAAGAGAAAAGGTTTAAAGTGGATAATGCTATAAATCAAGTTAACACTTTAAAGAGGTC

GCCAGGATATTGTGAGGCTCTCTCTTCAGTGTCCTGACGTGAGTGGGAAGAGTTTCACGCGTGAAAAACAAGGCCCTGTA

TTTTACGAAGTTTTCTCAATTCCTCGCCTTTAAGGAGAGGTAGAAGTTGAGCAAAGGAGATGGCTCAACAGTGAACTGTT

TATACACAATCCCTCTGAGCAGCGTACAATCTCCCCAAACCCACATAAAAGGGTGAGAGTGGTGTGGTCTTCTATTATCC

TAGTTCTGGGGAAACAGAGACAGGAGGGTCCCTGGAAGTGACAAAAGTCCAGCTCCAAGTTCCAAAGAGAAATTTCTACC

TCAAACATAATCACCA

>Sequence_360

ATGACGTTGTGGCGCGGCTGATACCAGTAAT

CGTAGTTGAAGTTCATACCCGTAGCGTCGTGNNTCCCAGCGTCCTGCCACATCAAAATTGCTGTCGAGCAGGAGGAAACC

GCCCGGAGCATTACCCTGACTGTCGCCGAGCATGGA

>Sequence_362

CTACAAGATCCCTTTAGCTCCTTGATTACATT

CTNTAGCTCCTCCATTGGGGGCCCTGT

>Sequence_363

AATGTCAGCTTCAACGCCGACTATGATCCGCT

GGAGGAGAAGCTGTCGGGCGGCAGCGAG

>Sequence_367

GGATCCCAGAGTCGTGGAGACCACTCACCGA

>Sequence_368

GGAACGATCTAAACCAATGTGGGAGCAGGCT

TGCTCCCACATNTGATTTGTGTCGCTCANGAAATGGTCGGCAGGCTTATTTACGCTGCAATAACCCNGG

>Sequence_375

TCTAGCCTTTTAACTTTATGGCAATAGTACAT

CAGAGACTGTATATTCTGACTTCATGAAATTAGTCATTTAATAGAGTCATAATGATTTTTCTCTTTTCTTCAGTGTGACC

AGTCATTCTAACTCAATCTTAGACTGGTCTAATATTCAGTCCAATATTAGAGATGCCTATATTCTAAATTACCTAGCAAA

GTTAAGTCAGAGCACATCCCCCACTTCCCCTA

>Sequence_377

TTAANNNGGGTANTTTGATTTTCTGGAGTCCACCTTCTTGAGTTCTTTATATATATTGGATATTAGTCCCCTATCCGATT

TGGGATAGGTAAA

>Sequence_379

ATATACCATCTGATATGAGCCCCCACCAACAC

ATGCAGAAGAGGACTGCCAAGGATGGACCCAGTCAGAGAAGAGGCACCTAAACCTCAAGAGACTGGAAACCCAATGGAGT

AGGGA

>Sequence_382

GTCTCTGTTTTCTCTTAGGCTATGTTTCTCCA

GCTCTGTGCAATTGTCTCCCAGGGCCTGCTTGCAGAAAATGCAACCTTGCTCTGCATCGTTGGAGCTCAGTGGCTTTCTG

GGACTTGAATGCAAACCTCTAAAACTCCCTCATGCTTGCATTCTTCATGGCTGCAAAATAAGTACTACATGAATAATACT

GCCAAGTTCTGATGCTGACTTGAGATGTGGTCTGTACTTCTTGTACTACAGCTAAAGTGGACATTCTGTGCCTTCAAGGG

TAACACGTCTCTTGGTGGCCATTCTGGAGTAGGGCAGTAGAGTTAGGTTCTCACCCATCGAAAGTAACGTATCTTAAACC

TGCTTTAGAAGAGTTCTGTATGCAGAGTCACATTTAGATTTAGGGCTGAGCTGGATGTGATTGTTGGCAATTACTCACTT

CTAAGCTTCCTTCTGCTTTGCCCTAGATTGCAGGAGTCAGAGACCTGGGAATTGAATGTAGAATTCTGGATGTCGCTGAT

GGTCTATCAGAAAGATGCGCTTGCCTTTGGAGGAGAAAGGAGCTTTCCCTTGAGCAGCAGTGGTGGCTACTTGATGTCCT

A

>Sequence_383

AGTTGGTAGCCACAACGTAACTCATACAGGCC

TAGGGTTCACTGTCACCACAGCTCAGAAAGCAATGGATGGTAAAGGGAGATGGGAGAATAGGCCCTTCAGGGGGACACTG

ACCCCAGAGAATTAAGAACAGGTAGCCGATCTCACCAGAGAGGGAACAGGCTCTGGTAACTGTCACCGGCTAAAACCAAG

CCTGCTGACTTGTTTCCTAAGTGGGAATAGTGACCAGCCTCTTCTGATGGTTGCAAGGCCTGCTTATGGCTGTCACTCAG

CTGCCCCTA

>Sequence_386

AAAGGTCTTAGTTTGTCAAAAAATAAAAATAA

AAGCTAGATTACTAAAAAGCAATACAAACTTTTTTTTTTTTTTGGTCTTTCCAGACAAGGGTTTCTCTGTGTAGTCCTGG

CTGTCCTGGAACTCACTTTCTAGACCAGTCTGGCCTCGAACTCAGAAATCCACCTGCCTCTGCCTCCCGAGTGCTGGGAT

TAAAGGCATGCGCCACCACGCCGGGCACAAACACATTTTTTTTAAATATTTATTTATTATTATATGTAAATACATTGTAG

CTGTCTTCAGACACACCAGAAGAGGGAGTCA

>Sequence_388

GTGCGAAGCTGTTTTCCCGTTGCGTTCGTCTC

GCGGTTGGTCAACATCTGCACCGTGTCTCNNTGCCGTTCCGCGCCCCGCAATGGGGCCTGTGCAGAGACGAGAAATAGTC

TGTTAACCCTAACGGACGGTTAAGGCGCCGGCAGCGGCGCCCGCGTTCCGTCCGGCATCAGGAAGTCTGGTCCATGCCGC

TGAAACTGACCGAAAAGCCCGCGCGCGACGCACGTCCCGTACACCTTGTCACCAAGCAGGGTTTCGAGCGGCTCGAACTC

GACGACCGGTCCCGCGCGTGGGCGGCGGCAAACGGCTTTTCCGGGCAGGCAGGCAGGTTGCTGGTGCTGCCCGGGTGAAG

GCGGCGCGGTCNCGGGCGCGCTGTTTGGGGGTGGCGGCCGAACGCGGCGGCTTTGCGCCGCT

>Sequence_390

GGATCCCCAGGACTGACATAAAAGGTAGACATGGTGG

CACACATCAGTTCTAGGGAGGTAGAAACA

>Sequence_394

GATCATTCATTCAGGCAATCAGAAAACACAGAACACTGAAGAAA

GAATGGATAAGAACCACACTGCTTTTGCAGATTGAACATAAGCACACAGTATCTGAAGCTTCTCGGGGCTGCAATTCACA

CCACACTTGTCAGCTGTTTGACCTTGGAAACTTCCTTAACCTAACTGAAATATAGGATTTCCCTTAATAAATTGAAGACC

ATTATACTGGTTTTGAAATGCTGTTGCTGTAATTTGCCACATTATGCAGAACCTAACATGCATTGAGAGTTCACTGAGTG

ATAACCATACACAGAACTGATTTTTACCCTTACATGTCTCCCGGGGTGATTTGTTGGAATGACATGTAAAAATCTCAGAA

TAATTTAAAAGGCAAATTAAGTATTTGTCATCAATCTTAGTGCATCAAATAAAAAGTGAGATTATTGACTCCTCTTTAAC

AAAACTGAAAGGTGAACCCTTTTACCTGTATCAGGGTCAAACTAACATGCTAATCAATGGTATGTAAAATATAAATAACG

GAAGCATATAGACATGTTTGGTTTTTGCATACTGTATTCCCATGACTCCCACCCCAGCCTGGACTCTGATATCCACCATG

GGAAAAATATGCTAATGCAGAGGTATTCCCCAAGACCCCTGAGTCAGCCTGTTTATATTAGAATATATCTAATAGCAGAC

CCTA

>Sequence_395

GATCCTCCGGTAGTACCATGTCCAATTTTCTGAGGAAC

TGCCAGACTGATTTCCAGAGTGGTTGTACAAGCTTACAATCCCACCAACAATGGAAGAGTGTTCCTCTTTCTCCACATCC

TCGCCAGCACCTGCTGTCACCTGAATTTTT

>Sequence_396

GATCATCTTGGCCTACAGCCAAGTCTGTGAGGA

ATTAATTGTCCACTGTGGGTAGTATCATCCCTATGCCGGCTGCATTGCCCACCATGGGTAGTATCAACCCTATGCAGCTG

TATTTCCCACCATGGGTAGTATTTTCCCTATGCAGGCTGTATAAGAAAATTAGCTATAGCTAGGCGGGGGTGGAGCACAC

CTTTAATCCCAGCACTTGGGAGGCAGAGGCAGGCAGATTTCTGAGTTCGAGGCCAGCCTGGTCTACAGAGTGAGTTCCAG

GACAGCCAGGGCTACACAGAGAAACCCTGTCTCAAAAAACTCAAAAAAAAAAAAAAAAAAAAAAAAAAGGGGGGCTGGTG

AGATGGCTCAGCAGGTAAGAGCACCCGACTGCTCTTCCGAAGGTCCGGAGTTCAAATCCCAGCAACCACATGGTGGCTCA

CAACCATCCGTTACGA

>Sequence_397

GATCCCACTTGGCTCATGCATACAGACCATCTGGGGT

TCCCAGTTTCTCTCTCCCTCTCACAGCCCAGACCCTGTTCTCCATAGTCTCAGATTTATTAAAGCTACTCAGTTTTTCCC

TGTGTCAAAGGTCAATCAAATGTTTGCAGTGTACTTTGAGCTGTTTAGAGTTTA

>Sequence_399

GATCCTGCGGCAATCGTTCAGAAAGCACTCACACC

>Sequence_400

ATCGCCAGNGCCCCCAAAATCTTTTATTCTGA

TATTTATGCGTTCATTTAGGATAAATAAAATTTTAAATAAGAATCCTGGTAANNGCTTGAGTGGGGCTGAAGCTCACCCA

TGTGTTATTTCATTTATATTTTGGTGTTGGANATTGAACCCAGAGCCTCACACAGGCTACAGCATGCTGTACCACAGAAC

CNTGCCNAANNNCNTNNCACCATATTTTTAANGGATTCCTAGGTGTTAGGGAGGCAACCCTCTTAGTGGAGGAGATTAAA

ATGAGTAGCAGGGCCTNGTGCACAAAGCTACCTTAGATACATTGCTTTGATTTGGGTTAGATTTAGATTTTGTTA

>Sequence_496

GGTCGAGCCGGCCTCCACCTGCGTCTGGGCCGCC

TTGGCCATCTGGGCCCACATGTACGCCAGCGCCGTCAGGCCGAAGACGTTCAGATAGTCGGTCGAGGCGGCGCCCGCGTT

GTCCGGGTTGGCCATGCCGTTCTGCATCAGCCACATGGTGCCTTCCTGCAGCTTGGTCTTCACGTCGGCCAGGCCGTCGA

CGAAGGGCTTGATGGCTTCATTGCCGCCGTTCTCGGCGACGAAGGCGTCGATTTCGCCGAACCAGGTCATGATGGCGCGG

CCGCCGTTGGCGGGCAGCTTGCGGCCCACCAGGTCCAGCGCCTGGATGCCGTTGGTGCCTTCGTAGATCATGGTGATGCG

GGCATCGCGCAGATACTGCGAGGCGGTGAAATGTTCGGTGTAGCCCGAACCGCCGTGGACCTGCATGCCCAGCGACGCGA

CGTGGAAGCCCTTGTCGGTCAGATAGGCCTTCAGCACCGGCGTCAGCAGGCCCATGTAGTCCTTGGCCTTGGTGGCGACG

GCGGCGTCTTCGGACTTCTCCAGATCGGCCTGCAGCGCGGTCCACAGGATGAAGGCCTGGCCGCCTTCGACGAAGGCCTT

GGATTCCAGCAGCATGCGGCGCACGTCGGGGTGGACCATGATGCTGTCCGCCGGGCCTT

>Sequence_498

GATCTGAACTTTCCTTACATTTTTACCTAGTTTGGGTGTCAGGGT

>Sequence_502

GCNTGCGGCGGCNNNGATCGGGACNTCTCATGTGACAAGCTCCTGAAAGAAAAGGGCGATGATCGGGTCGATTTCCCGCG

ACGCCCTCATACTAGAATACGCGGACGGGCGCCGCATCCCTCGCCGCCTGGAGGTCG

>Sequence_503

GAAACTGGAGC

>Sequence_505

GATCAGGCCGGGACCTCCGGCGGCGCGAATCATCATGATATGCACTCC

CTCGTTCACCGCCCCCGCAGCGCCCGTAAGGTGAAGTTGAGCGTCAAGGTTGTGGCGGCTTTACGGCGGAGGTTTCGGGT

TCGACCAACGTC

>Sequence_507

GATCAGGAGACTAGTTAGGAGACAGACAGTTGGAAGGTGCAGTCCGCTGCTTG

GCAGGGCAGGGGTTGGGACATAGGGCTATGCTTGTCACATTCCATTACTGTCGTAAGATAACCTGAGTGCCCTTGTTTGC

TTGCTGTTGCTGTGATAAACATCATGACCAGAAGCAACTTGGGGAGGAGAGGGGTGACTGGTTCACATATCTGGAGCACG

GTGGACCATTGAGGGATGGTGGGCAGGACCCGTGGAAGGAAGGTGCTTATATTATAGGCTTGCTCTTCCTAGCTTGCTCG

GCTTTAACTTTATAAAAAAGATTCACTTATTTATATAGTTTATGGATATGAGCACTCTATTTGCATGTATGCCTGCATGC

CAGAACAGAGAGCCAGACAGAAGAGAACCTCA

>Sequence_509

GATCTTCAGAGTAGTTGATGTTAGTTTTGATTGTTGTGTGTTGTTTTT

CTTTGACTTGTTCATTGACCAAGATTGGGTATTAAATTTACCTCCTATCCTTGTACCAGAGTCTATTTCTCCTAGTAATG

CTATTATTTCATAAAATTGAGTGCTCTATCAGAAGTGTATGTATACTTGAAATCCTTTCTCTATGTCGCCAAGTACTAGA

ATCTAATGTTTATATTTAACCCTGATTACATATTCATTATTCCATCTCACTTTGCTCCTTTCTTCCAAACTGTTAGCAAT

GTCTATCGGACATTGAAGTTGACTAGATTGAAACTTATCTCAGGGAACATATGATATATTTCTTTCTGTATCTGCTTTAG

TTCAGTTAATACAGTGTTCTCTTCAATCTTTTGGCTGGAAATAATCAGGATTTCAGTCTTTTGTGAATTTATATTCTTCT

GTATCTATATAGCACATTTTCATTATAAATTTATCCATGAATGCCCACCTACTTTGATTTCTTCTCATAGCTATTATGAG

TACTGCTACAGCAAACATAGATATGTTGGTGTCTCTTTGCTACACTGATTTTATCTGCTTTTTAAAATGTGGAACTAGAA

GTGTAATTATTT

>Sequence_510

GATCCCGTGGTCTGACAGCAACAGCACCATAAGCTACTACAG

CAGAGCATACTTAATCGATTGTCTGATACTCTCCCTCCTCACTACCCGGTAAGGCTTGGCTCAGAGCAGGCCTTCATTC

>Sequence_511

CAGCAAATATCTTCAAAAAGAAGAAAACTACCCAAACT

TAAAGAAAGAGATGCCCATGAACATACAAGAAGCCTACAGAACTCCAAATAGACTGGAGCAGAAAAGAAATTCCTCCTGA

CAGATAATAATCACAACAATGAATGCAATAAATAAAGATAGAATATTGAAAGCAGTAAGGGAAAAAGGTCAATTAACATA

TAAAGGTAGGCCTATCAGAGTCACACCAGATTTTTCACCAGAGACTATGAAAGCCAGA

>Sequence_512

GATCCTTTGAAACGTCTGGGCTATGAACATCCGAGTTGGCCATCCA

TTCATGGTGGGGGATAAGAAAACAGACACTTTGCACATGTCACGGGTGGCTTAGTGCATATGCACCACTGATGACTATCT

TGCTCAGAGTGAACTCTCCAGTCCAGGTAGCCCTGGAGAAGAACAAGGGTGAGACCTGCAAATGCTGACATGACCTGTGT

TTCAGAAGTGTCTTAGCAGGGCTGGAGAGATGACTCAGCGGTTAAGAGCACTGACTGCTCTTTCAGAGGTCCTGAGTTCA

ATTCCCAGCAAACACATGTTGGCTCACAACCATCTGTAATG

>Sequence_513

GATCCTACTCAGCGCAGGTTCACAGAGGAGCAGACACCCATCATTTGA

ACCAAGTCACAGGTCCTCTTTAAACTCATCCAGTTTCTAGCTGTCTTCATGACATCTGTAGTACTTAGCTGCTCTAACAA

GCCATGTGCAAGTTTTTAAATGAGAGGCAATGTGGTAGTTTGAAAGAAAATGGTTCCCATA

>Sequence_514

GATCAGAATGCTGACAGGCATCATGTATGCACAGAAGTATCCAGAAAT

AATATACATATGCATACATATATGCAACATATATACATATAGATATACGTATGATCTATTCCACAAGGCCATGTAAATGA

CAGCTACAGGGAAGAACACAGTTATCAGGACAACATTATCCTTTCTCT

>Sequence_516

GATCTATAGACATGGCTTTAGGTGCTTTGAGCAGTAG

AAGAAAATAACAGGAATCCCATTGAGATGTTAATAGACTGTCACGTGGTGTGTGTGTGTGTGTGTGTGTGTGTGTGTGTG

TGTGTGTGTGTTATATCTCATGCTCAGATTTAATCCCACAGCACTTTCCTGGCGAGTCAGAGTATTTTGAGAAGTAAGCA

GTCCTCATTGGGTATTGTTTCTGTGAACTAAGTCTTCCTATCCTACTCAAACCTCAGAATCTGCTTAGGTCTATCTAAGA

CTGATACAAAGAGAATGAATATGAAGTAGTCAGTGTGCTCCTAACTGAATTGTTTATATGATATACATTCTTATGTTTTT

TAAATTGTCCCCTTAAGTAGATTTTAATGATACCTGTAAGGATGAAAAGATGAGTAAACCATGTGACAACAGGTGATTCA

TGAAAAAGAATTAATTCTCCATGATTCATTGATCTCTGTATTGTGTTCAGTTCTTGATTTCTCTGATGGTCTTCATTTGC

TGTGAAGAGAAACATAACGTTTTTACATATACTATAATCTGATTATGGCTTTCCTTCCCCCATCTCCTCCCCAAAATACC

CTACTTT

>Sequence_517

GATCGGCACACCCGTCGTTTTTGCCTTTCTGTCCGTCAA

CCTGTTGGGGGCTTGGCTGTTTCTGGGTGAAGCGGCAGGTTTGGCACAATGGGCGCGCAACACCACCGCCTCCATTGGCA

GCTTTTCCTTAACCCCCATTCCTTTATTCGTCTTGATGGGCGAGGTGTTGTTTCACACCGGGCTGGCCTTCAAGGCCATT

GACTCCATCGAGCGCAT

>Sequence_526

GATCATCAAGGNAGATGAAAGGGTTTGCTGCTGAAAACC

CAATGGTGACACAGCTCGGCATGATTGCGGGCGGCGTAACCTCGGTCATCCTGACGTTGAAAGGCTTGTTCGGCGCGTTC

AGTCTTGTTCGCACCGGGTTGAGCGTGTTCACCG

>Sequence_527

TGGATCCACCCCATAATCAGCATCCAAACGCTGACACCATTG

CATATACTAGCAAGATTTTATCGAAAGGACCCAGATGTAGCTGTCTCTTGTGAGACTATGCCGGGGCCTAGCAAACACAG

AAGTGGATGCTCACAGTCAGCTAATGGA

>Sequence_528

GATCGCTGCCATGAAGTTCATGGTGTCGCCGCGGCCGAAG

CTGCCGTGCATGCCTTGGCCTTGGCGCAACACGGTGTCGGCGACTTCCACCGAACACACCGTGGGCTCGTCGCAGCCCGC

GGCATAAGAGCGGAAGTTGACGACGATGGTGGGACGCGGGAGCTTCGACGAACCCTTCAGCGCGATGGCCGACATCGGCA

GCGTGCCCGGAAAATTGCCGA

>Sequence_529

GATCCCCGCGCCCATAACCGCTCGCCCGAAGGCCGNATGGCCGNCGGCGCGCG

CGTCATGCGCCTCGTCGCAGCCGC

>Sequence_530

GATCAGGTTTTTGGCGGCCCGCTCTTGCCGGGTCATGCCGTCTGCCGT

AGCAACGCTTGATTTCTGTAGCGACTCGTTCGACTTGCGCAGACCGTCATTTGCTTTCGTAGCTTTTACGGCGGTGTCTG

CACCCGCTTGAACGCGCTTGCTGGTTGCGTCAGTGGTAACGCGCAGGGATTTAAACGACGC

>Sequence_531

GATCATCAAGGAGATGAAAGGGTTTGCTGCTGAAAACCCAAT

GGTGACACAGCTCGGCATGATTGCGGGCGGCGTAACCTCGGTCATCCTGACGTTGAAAGGCTTGTTCGGCGCGTTCAGTC

TTGTTCGCACCGGGTTGAGCGTGTTCACCG

>Sequence_532

GATCAGGTTTTTGGCGGCCCGCTCTTGCCGGGTCATGC

CGTCTGCCGTAGCAACGCTTGATTTCTGTAGCGACTCGTTCGACTTGCGCAGACCGTCATTTGCTTTCGTAGCTTTTACG

GCGGTGTCTGCACCCGCTTGAACGCGCTTGCTGGTTGCGTCAGTGGTAACGCGCAGGGATTTAAACGACGC

>Sequence_533

GGATCCCCAGACTTAAAACCTGGCCATGGTGGCGCTCGCTGGAAACTCC

ATCACTGGGGAGCTAAGAGGCAGGG

>Sequence_534

GATCTGGGCCAGGCATTGTAGTGCACACTTATAACC

CCAGTGCTCTGAAGGCAGAGGCAGGTGGATTTCTTTGAGTTTGCCGCCAGCCTAGTCTACAGAGTGAGTTCCAGGACAGC

CACAGCGATGTGGTGA

>Sequence_535

CCCAGAGACCTTCCCCAGAAGTAGCTTGAGCGTCTGG

>Sequence_536

GATCCATTCAGATCCGGACAATACCCTCCCCCATCTTCTGCAT

GAACCCAGCCGAGGCCATCTAGAGCACCACAGGACTCTCCATGCCTCAGGACCCCTAGCACACCCAGGACCTCCT

>Sequence_537

GATCTTACAACATGTAGAAGTTGGACAAGGCTACAAAGAG

TCAATGGGAATCATTTGCTCCAGCAAAGCTTCACATCATCAGATATATAATCTAACCCAAGACAATATATTCCAGCTACC

TAAGGAAAATGAAGTTATGCCATTTGCAGGAATTTGAATGTAACTAGAGATAACCATATAAAGTAAAGTAAATCAGTCTC

AGAAAGACAAATATTGTATTTCTCTTAATTGTGGGACCTACATTTCATACAGCTGTAGAAAACTCTATATGTATAGATAT

CATGAATTAGAAATTAAATTGTTTATAGCAATAAGAAGGGCCATATAACAGAGTGACAAGAAAGAATAGAGAAAGTGGGA

AAGAACCTTGAACTCAGTGGCACAGGGGGAAATCTACTAAACAGAACTTCAATGGCTCATGCTCTAA

>Sequence_539

GATCGCCTTGCAGGAAATCTTCCAGCTCACGGGTCAAGC

GCAGCTTGTTGTCCGTGGACAGTTCGATATTGATGCGTTCGATTTCTTCGGTGTATTCACCCATGCGGCGCAGGGGGATT

ACCACGTCTTCGTT

>Sequence_540

GGAATTCGCTTTGTGGGCGGACGCGCAATACGA

AAATCGTCGTCCCCGCGCAGGCNGNG

>Sequence_541

GATCCTGCCAATCCATGAGAATTGGAGATTTTTCCATCT

>Sequence_542

GATCGTTCTGGGTCTCGCGGCAATGTCGGCGCAGAAAGGTCTCGTC

GCGCAGGCGCGCGTCGCGCTTTCGGACAATCACGCCATCGACCTGTCCGAAATGGGCGGCCAGTCAATCGGTGCGCTGGC

GTCATGGTGGTCGGGGCATGACCTGACCCTCACCATTGCTCTGGTCGCCGGCCTCGCCATGTTCTGCTGGATATTCAGCG

AACGCCGTTATCGCAGCCACACGGGCTC

>Sequence_543

GATCCAATAGATGTCCCTCCCCATCCAGTCCAGGCTGG

TTCCTTCCAAGGTTTCAAGTGTTCTCGGCTGTAGGGCTCCTTCCCTGCCCACAGAGGGCCTGGAGGCCCTTAAAAACACC

AGACAGGATTTTTCAGTATCTTACCAAGACCAGCTGATTTATTCTAT

>Sequence_544

GATCGTCAGCTGTGAAGCCGTAGAAGGGTCGGACAAGCTG

CTGCGCCTGAGCCTGGATGCTGGCGAAGGTCGTTTGCGCCAAGTGTTCTCTGGCATCAAGTCGGCTTACCAACCCGACGA

CCT

>Sequence_545

GATCACTGTGAGTGATTTTCTGAGGCAGGGGGATGACTGTGTTTAA

TTTTCTGAGGCAGGGGAATGACAGTGAATGGTTTTCTGAGGCAGGGGGATGAGAGTGAGTGATTTTCTGAGGCAGGGGCA

TGACTGTAAGTGATTTTCTGAGGCAGGGGGCTGACTCAGTGATTTTCTGAGGCAGGG

>Sequence_546

GGATCCGGGGCTCAAATCCCTTCTGGTCCACTGCAGC

ACCGGGGTCCCTGGCCCGGGGAGTCTCCGGACACCCACAAGGACCCACACA

>Sequence_547

GATCCTCCGGTAGTACTATGTCCAGTTTTCTGAGGAACCGC

CAGACTGATTTCCAGAGTGGTTGTACAAGCCTGCACTCCCACCAACAATGGAGGAGTGTTCCTCTTTCTCCACATCCTCG

CCAGCATCTGCTGTCACCTGAATTTTT

>Sequence_548

GATCCTGCTTTGCAACTCAGTCCATCTGAAACAAGCAACAG

AACCCAGCCGATACAAAGGGACATCTCCTCGGGTTTTCCCATATATGTTCAGTGCTGTGCATTAAAAATTGAGCCTA

>Sequence_549

GATCTCACTATTTCCCCTGCAGCTGCAGTGTCCAGGCACAGACGACACTCAGG

GCTGTCCGTGCTTCGCTGAAAGCACAGTGCCTCTCACACTGTGTGAAGACAAGGTTCTCCTCCCCTCAGAAGTGCGCCAT

TAAAGGTGTGGGGATGCAGCTAAGCAGGGTACTTGTCCACCACGGAGGAAGCCCTGGGCTCAATCCCCAGCACCTTGGGT

AACAGACAGAGTGACACACCCCTGT

>Sequence_550

GCCTGGTGGTGAATGGGAACCTGGTCCACACGGC

AGTTGTGCCACAGCTGCAGCTCGTGGCCTCCAGGGTGAGTGGAATGCAATATGGCCTTAGCATCCTGAGCGACCTGCTCA

TCACACCAGGCCAGCTGTTCTGTGTGTGGATGCCAAAAGGGAGGTGGAACTGCTGTGTGGATTGGTCAGCAGGGTTACCG

TGTGTGTGTGTGTGTGTGTGTGTGTGTGTGTGTATGTGTGTGTTTGTGTGTGTGTGTGCCCTCCTTGTAGGGTTGGTGAA

GTGGCTCAGCAAGGGAAGCACTTGTACACACCTGATGACCTCGGCTTAGTCCCTGGAGCCTATGTGGGAGGTGGAGACAG

GACAGCTCCACACAGTTCTCTCTGCTTCAGGAGACCTACTGGTCCCAGGGCAGCTCAGGCACTCAACTGTGTAATTGCAC

CTTCTTCTCTTCATCTAACTATGATTGGTGGTGTGTCTTGTCTGTCTGTCTGTCTGTCTATGACTGTATATGTATGCATG

CACCACCTGCCTCANTCAGCTCCTTCCTTTCACAGTGTGGCTCCCA

>Sequence_551

GATCGCCTTGCAGGAAATCTTCCAGCTCACGGGTCAAGC

GCAGCTTGTTGTCCGTGGACAGTTCGATATTGATGCGTTCGATTTCTTCGGTGTATTCACCCATGCGGCGCAGGGGGATT

ACCACGTCTTCGTT

>Sequence_552

GATCAGGAATTCAAGGCCCGTATCTAAACATGATAA

AAGCAATCTACAGCAAACCAGTAGCCAACATCAAAGTAAATGGAGAGAAGCTGGAAGCAATCCCACTAAAATCAGGGACT

AGACAGGTCTGCCCACTTTCTCCCTACCTCTTCAACATAGTACTTGAAGTCCTAGCCAGAACAATTCGACAACAAAAGGA

>Sequence_554

GATCCCTGGGGATTTCTTACTAGAATCTAGTTTGAAGAAT

ATATTCTCAGCCTCAATAAAATTATAGATTATTATGGATTGCTACAGATCCCTTTAGCTCCTTGATTACATTCTCTAGCT

CCTCCATTGGGGGCCCTGT

>Sequence_555

GATCTACAGAGTGAGTTCCAGAACAGCCAGGGCTACACA

GAGAAACCCTGTCTCGAAAAACCAGAAGGGGGACGGGGTGGAGAGAATAACATATTATTAAATGAGGATAAACAGACTTA

TGGAGTTTTGTTTTGTTTTGTTTTGTTTGAGACACAGTCTCATTGTGTTTCTCTGACTGGCCTNGAACTATGTA

>Sequence_562

GATCAGTCGGGCGGGAGTCGCGCCATACCTCCCAGCCCGCAGCGCAATCCGGC

CGACCCGCTGGAAAGCATGCTGGCGATGTTCGAGGCAACGCCTGAGCCGGAGCAGTCCGTCGAAGCCCCGCCAGAGGGTG

CCGACGATGCAGCGGCGCCGCTCGACCATCCCGCATCGGCCCCTGGTCCTTCGGCGCCGACCGCCGCGCCTGCCGCGCAC

GATTCCGCCTCATTTACCGGGCGGCCATCGGGCGAGCATTTCATGGCCTGGCTGAAACAGGCCGTCCAGACCCGCAAGCT

CATCATCAATGACGCCAAGGCCCTGGTACATACCGTGTCCGACACGGTTTACCT

>Sequence_566

CCAGTTCCAAAATAAAAAGCAAGTCAAAGCACTGCATTCTGAATTCCA

AGGGGTAAGGAAGTGGAGATGTGAGGCTTCTTGGTTGTTACTGTTTGCCCAGGTAATCTAGTATAATCACTTAGCCTCAG

GTTCAAGGGTA

>Sequence_569

GATCGCGCTGGCCGACCATGGCCATTCTCAACCGGCGTTCGAG

CAGGAAATGCGTAACACCCCGGAAGTGCTGGACTGCTGGCTGGTCAGCGGCAACTTCGATTTTCTGGTGCGTATTGGCTG

CCGCAGCATGGAAAACTACCGGCTGCTGGCCGATACCTGGCTGACCAGCAAGAAGTTCCGGGTGGATAAAATCGTCACCC

TGACCGAACTACAGTC

>Sequence_572

GATCGATGTCGGCGCGCAGCGCGCAAGCGGCCGCTTCCGACAGCAGCG

CGATCGCGCCCAGCGACACGTAGTTGTGCAGCAGCTTCATGCGGTGGCCCGCGCCCACCGGGCCGGCGTGCGCGATGTTC

TCGGCAAAGCACGCCA

>Sequence_574

GATCGCGCGGCCGTGGCGGTCGTTGTCATACGGCCTTTTCCTC

CCCATCTGGCGCACCTGTGACCTACGCACCCGCTCTCATCCACCCGCAGACCGCAACACGCAGCCGTACGCTGCGCGTCG

TGAAGCGCTGGGCGCTGATGGTCGGCGGACTGTTCGTGGTGCTGCTGGGCATCCT

>Sequence_575

GATCTGAATCCGCAGCTCAGCCGTCGCTTTCATACGCTCGAACTCGGC

CACGCGATACATCAGCTTGCGCAAC

>Sequence_578

GATCAGGGTTTCTAAGGGCTTATCAGGATGACCAAGATATCTGAGTCTATATTCCT

GTCCTAGCCCAAAGTTGTATTCTTGCCTGAAGCCTACTTCTTTATTCTAGCATAAAATCAAATTCCTGCAT

>Sequence_579

GTTTTGTGTGGGATTCTGTGAGAATCTGCTACAGCCCTGGG

TGTTCAAATCCCCTCCCTACCATTTTTTCCCCCTGTCTCCATAGCTTAGCACTCAAATGATTCTTTGAGAAGGCAGGGTA

GGCACAAATGGAAAGCCTTTTCTTTGCTTCAGTTCTAGAGTCTGGCTTGTATCTTGCCAATTCTGGGAGATTATAGGAAT

GATTTAGAACTTGTATGGAAAACTACCTGACCTCTTCCAGGGAGACCTCAGGGAATTCCTTAAAATATTTAACTTTTCTT

ATGTCTACTTTAGTGCTAAAGAAGAAGATTTCATCCTACT

>Sequence_580

GATCTGCACCTTGCGGCGCATGTCCGGCGTCAGCGCCTCTGCG

GCGATGTCGACCTTGCGGCCTTCGATCAGCAGCTCACCGGACGTTGCC

>Sequence_581

GATCGGCGGCTTTACGAAATCGCGACGCTTACGCATTTGCGA

GACCGACTGAATTCGCGAGACGTCTGGGTCGAGGGTAGCCGGTCATTCCGGCCGATAGACGAACATCTCATGCCGAAGCC

GGCCTTTGTCGCCCTGAAGGAGGAAAATGAACTCGGCCTCGGTGTCCCGAGCGACGGTGCGGCTTGGCTCGCGGAAGTTC

GCCAGATGATGGATGTCAACCTCAAACGGTTGGCTTGGCGAGCCCGTTTCGGGAAGCTCGATGGCGTCAGAATGGAAGAC

GGCACACT

>Sequence_582

GGATCAAACTTGCCCCAGGGNAACCCCTAATCATCAGGAAGT

GGTCTAACAATGCTGCCCCCTTTCTAGCCCCTGACTTGATTCAG

>Sequence_584

GATCAGTCGGGCGGGAGTCGCGCCATACCTCCCAGCCCGCAGCGCAATCCGGC

CGACCCGCTGGAAAGCATGCTGGCGATGTTCGAGGCAACGCCTGAGCCGGAGCAGTCCGTCGAAGCCCCGCCAGAGGGTG

CCGACGATGCAGCGGCGCCGCTCGACCATCCCGCATCGGCCCCTGGTCCTTCGGCGCCGACCGCCGCGCCTGCCGCGCAC

GATTCCGCCTCATTTACCGGGCGGCCATCGGGCGAGCATTTCATGGCCTGGCTGAAACAGGCCGTCCAGACCCGCAAGCT

CATCATCAATGACGCCAAGGCCCTGGTACATACCGTGTCCGACACGGTTTACCT

>Sequence_585

TAGTTTGAATCTTTAAATAGATGACTCTGGAG

TAAATATATTATGAAAAGCTTCATTTAACTATGGGTTAGCAGCCAAATAAAATAATACATCCTATATTTTAATGAAACTA

TTTTAGTTTTGTCTTCAATTTCATATCTCTCTCTCTCTCTCTCTCTCTTTCCTCTCCACACTCTCTCCATTCTCCTCTCT

CTCCTCCTTCTCCTCCTCTCTCCTCTCCTCTCAGGAAGCTCAAAACATCTGTGTCAGGGTAGAATTTACAAGAATGTAAC

TGTTGTAAGGAGGCTGAGAATTAAAGCCCCCAAATGTGAGATTTCTAAGGACTGTCCATCAAACCTCCCTTCCTTCAGAA

GCAGACACCATGTCTGAAGAAGGCACCTGACCCGAGTGATGTGCAGTTGAGTCTGGGAGAGCCTCAAGCCACTGCTGAGC

TGG

>Sequence_586

GATCGTGAGGACAGGGCAACCCCAAATCCAGCTTCCAGTGACCAGAAAGGACA

GCCAAGACAAAAGCAAAGCGCACGAGAGTAACAACA

>Sequence_587

GATCACGGCCTCGGCCGGGCGTTCAGGCCGAATTGCGGCCTA

AACCTCAGACCCTGCCAAATCAGCGGGCGCTCTGCGTCTCGCCCGCACGCCACAGGCGATAGCGAACCTCGACGTCCTGC

GGCGCATAGACCACCAGCGGCAGGCGGCTGTTGTAACGGACAAGCGGCTGTTCCGGCGCGGCGACAAACACCTCGCGCTC

GCTGTTGGCCGGGCATCCCATCAGCGTCCCCGCCATCGCGCCCAGCGCGGGCAGGACATAATAGTCATAGCCCCAGCCTT

CCGCCGTGCGCGTCTC

>Sequence_590

GATCATCGCCTCGTGTATCCCTAGGTGGCTATGTCTTCACACA

TATACAGAAAAGGATAGCTGGGTCTGGGTTTAGTCAGAGAAGATGCAACTGACCCTAAAGAGACCGAGGTCTCTGGAAGT

TCAGAGGTCTGGTGGGGTGGGTTTGG

>Sequence_591

GATCTGCGCTGTTTACAGGTACGGCTGATGCAAACC

ACGAAGACCCACATCGTTCATGGCGGAACCCTTCGCTATCTGAAGCACGACAGCGATACGACCGGCACGCCCATGACCCT

GTCGGTCTTTGTTCCGCCCGGAGAGGGGCCGTTTCCGGTCCT

>Sequence_592

GATCCTCCCGTATGTACTATGTCCAATTTTCTGAGGAAC

CACCAGATTGACTTCCAGAGTGGTTGTACAAGCTTGCAATCCCACCAACAATGGAGGAGTGTTCCTCTTTCTCCACATCC

TCGCCAGCATCTGCTGTCACCTGAATTTTT

>Sequence_593

GATCTGACCCGTATCTATCCTGGCCCGGAGCCAGGGCAGGG

CGGTGCGCACTCCCAAGAGGTCAATGAGGCCGCGGCCTCCTTCCTGAACGAACCACTGACCATTGCGACCAAGGGAGCCT

>Sequence_597

GATCTTTACCTATCCTAAATCAGATAGGGGACTAATATCCAA

CATATATAAAGAACTCAAGAAGGTGGACCTCAGAAAATCAAATAACCCCCTTAAAAAATGGGGCTCAGAACTGAACAAAG

AATTCTCACCTGAGGAATACCGAATGGCAGAGAAGCACCTGAAAAAATGTTCAACATCCTTAATCATCAGGGAAATGCAA

ATCAAAACAACCCTGAGATTCCACCTCACACCAGTCAGAAAGGCTAA

>Sequence_600

GATCAAGGGGGAAAGGCCCCTTGTCGGTGGGACCATCT

CTGGGCTGGTGGTCTTGGGTTTTATAAGAGAGCAGGCTGAGCAAGCCAGGTGAAGCAAGCCAGTAAAGAACATCCCTCCA

TGGCCTCTGCAACAGCTTCTGCTTCCTGACCTGCTTGAGTTCCAGTCCTGCATCCTTTGGT

>Sequence_602

CGCTTACGCATTTGCGAGACCGACTGAATTCG

CGAGACGTCTGGGTCGAGGGTAGCCGGTCATTCCGGCCGATAGACGAACATCTCATGCCGAAGCCGGCCTTTGTCGCCCT

GAAGGAGGAAAATGAACTCGGCCTCGGTGTCCCGAGCGACGGTGCGGCTTGGCTCGCGGAAGTTCGCCAGATGATGGATG

TCAACCTCAAACGGTTGGCTTGGCGAGCCCGTTTCGGGAAGCTCGATGGCGTCAGAATGGAAGACGGCACACT

>Sequence_603

GATCAGAACTCTGCAGTGGTCTGAGGTGAGAGAGTCACGTCTGCC

CAAGGGCTTCTTCTAAGTAAATGGGCCTGTATGGCACTGGATTGATGTCACTCCTGTTGCTGCTGACCTGTGTCTCTGGT

ATCCTGTCCCCTGACTGGAGTCCAAGGCA

>Sequence_605

GATCAGGCCTTTCAGATCATCTGCTGCTACCTAGGGGTTCAAGGCCA

CCTTTGTGAAATCTTGTCTCCAAGGGTCAGCATATGAAAAGAAGTGTCTGTGAGCAGTGGAAACCCCACCTTCCTGAGAC

AGAGGCTC

>Sequence_606

GATCCAGTGGGTAGAAGGTCCTGGGAAGTTAGGTAGACAGAGGT

CTATTGTTAGTTTACATATGATTATTTTTAAAGGAACCTTTATTTTGCTCTCTTGTTTTTTTTTTAAAAGGCTTTGCACA

TTATTCCAGTAGAATATTCTATTCCATTACTCTTAGAAAGCACAGTGCACAAAGAAAAGATGGTATAATCCCCTTAAAAT

AAGTCCATTCTGCAGGAAAGCCCTGGGATGAGTTGAGAGAGCCCTTCTCTCTGGCTGTATAACACAGCATATCACTAACT

CACAGACTGAAGCAATACACATTTACCTCTCACAGCTCTGACCTGGGA

>Sequence_607

GATCTGAATTCAGACTGTCAAGATTGCAAAGGCCAGAGCGTTACTGG

CTGAGTCATCTCTTCATCTCGCTTTCCACATTTTCTACATCGATTTCTTTAATAATTATGCTAGTGACCTAGTCTTGACC

GGGATGCTGCTCTGAAATGAACTGCCTTCTACCCACTGGCTTCAGGCCACCAAATGACTCAGCCCTTTTGTTTTGAACAC

AATGGACAAAAAGGGGTCATGAGACCTGAGCCCATAGCCAGGACTTTCTGTTTACTAGCGGAGCCTATGTAACTCTATCT

ATAAAACGTGGTCACTGATATTTTCCCACAGATGAAATGAAAAACTTAAGAAGAGCTTAAGGAAAACAGTTTAATACTAA

ATCACTAACTTCTTTATGCCATCAGGCAGACAAGCTACTTCACCTGTGAAAGTCCATCTGTACCTGTCAGAGTTGATAGT

AACTCCTCTGGGGACTCATGTGTTACTCAGTTGATGTCCAACTGAACACCTCATGTAGTGTTGAGAAACCTAAGCACCAC

CCGTGGTAATTTTGAAGCATATATTTTTTTT

>Sequence_608

GATCGACGCCCTCGCGCACATCCCTCAGCGCCGCCCTTGCATGGTCC

CGCGCCTGCTCGAGCGAATAGGCCTCCTCCACGCGACCGAGGGTCAATTTTCGCAGCTGTCCGCCACGCCGGTAGGTGAA

GATGAAGCTTTTGGCGCCCGACGGCCGGACGCGCACGCCGAGACC

>Sequence_609

GATCGATGTCGGCGCGCAGCGCGCAAGCGGCCGCTTCCGACAGCAG

CGCGATCGCGCCCAGCGACACGTAGTTGTGCAGCAGCTTCATGCGGTGGCCCGCGCCCACCGGGCCGGCGTGCGCGATGT

TCTCGGCAAAGCACGCCA

>Sequence_611

GATCCATCCACTACCTGATTGTGAGCATCCACTTATGTGTTTGCTAGGCCCCTGCAT

AGTCTCACTAGAGACAGCTATATCAGGGTCCTATCAGCAAAATCTTGCTAGCATATGCAATGGTGTCAGCATTTGGACAC

TGATTATGGGATGGATTCCTGGATATGGTAGTCTCTAGATGGTCCATCCTTTTGTCTCAGCTCCAAACTTTGTCTCTGTA

ACTCCTTCCATGGGTGTTTTGTTCCCAATTCTGAGAAGGGGCAAAATGTCCACACTTTGGTCTTTGTTCTTCTTGAGTTT

CATGTGTTTTGCAAATTGTATCTTATATCTTGG

>Sequence_612

GCCTTTCCTTCAGTCTCTGCTCCACACTTTGTTTCCATA

TTGCTTCCCTTGAGTATTTTCTTCCCTCTTCTAAGAAGGAGTGAAGCACCCACACTTTTGTCTTCCTTCTTAAGTTTTAT

GTGGTCTGTGAAATGTATGTTGGCTATTCCGAACTTCTAGGCTAATATCCACTTATCAGTGAGTGCATACCATGTGTGTT

CTCTTGTGATTGGGTTACCTCACTCAGGATGATGATTTCTA

>Sequence_613

GATTACAGACAAGAATTGCCATGCTCACTTTA

TCCCTTCCAGCATTTTCATTTAACTACATCACTTGGCTTCTACTTTGCCAAAACCACAATTTGCCAATGGCCTGAAGAGC

AAGCTTGATAGAATACAACAAGGCTCACAATTTGATGTGGCTTTTTAAAAAATTTATTGGTTATTTTATTCATTTACATT

TCAAATGTTATTCACCTTCCCAGTTCCCCTCCTCAAACCCCATAACCCACTCCGCATCACCCTTGCTTTTATGAGGGTGC

TTACCCACCCACCCACCCACTCCTCCCACACTGCCCTAGCATTCCCCTGGGCTGGGGAATTAAGCCCTTACAGGACCAAG

GGACTCCCCTCCCATTGATGCCAGATAAGGTCATCCTCTGACATGGAGCCATGGGTCCCTCTATTTGTATTCTTTGGTCC

CTGGGAGCTATGGGGGTCTGGTTAGTTGATTTTGTGGCTCTTCCTATAGGATTGCAAATCCCTTCCCCTAACTCCTCTAT

TGGGATTTCCATCCTCAGTCTGATGGTTGGCTGAAAGCATCTGCATCTGTATCGGTAAGTCTCTGGCAGAGCCTCTCAGA

AGACATCCATATCATGCTAATATCAGCAAGCACTTGTNGGCATCAGCAATAGTGACTGGTTTAAGTGGCTGCATATGGAA

TGAATCCCAGTT

>Sequence_614

CCTAGATTCTTTGCTCTTTTTCTATGACTGCTCCGGCAATCCCATCTCCGAGC

ATTTCCACCCCACAGTGATCGGGGAGAGTATGTACGGGGATTTTGAGGAAGCCTTTGACCATCTTCAGAACAGGCTGATC

GCCACCAAGAACCCTGAAGAAATCCGAGGTGGGGGTCTCCTTAAGTACAGCAACCTCCTTGTGCGGGACTTCAGGCCTGC

CGACCAGGAGGA

>Sequence_615

GATCCTCCGGTAGTACTATATCCAGTTTTCTGAGGAACCGCCAGACTGATTTCCAT

AGTGGTTGTACAAGCCTGCAATCCCACCAACAATGGAGGAGTGTTCCTCTTTCTCCACATCCACGCCAGCATCTGCTGTC

ACCTGAATTTTT

>Sequence_616

GATCCTGTGGGACAGGAGTTACGGATGGTTGTGAGCTACCATATGGTTGCTGG

GAATTGAACTCTGTGCCGGTGATGGCTGAGCCATCTCTCCACTCCTTGTTTCTTTCTCTTTAAAACAAAAACAAACCAAA

ACCCCCTTAACTGTCTATTTTTTGTTTTTGTTTTGTTTTTTGTTTTTTGTTTTTTCGAGACAGGGTTTCTCTGTATAGTC

CTGGCTGTCCTGGAACTCACTTTGTAGACCAGGCTGACCTCAAACTCAGAAATCCGCCTGCCTCTGCCTTCCAAGTGCTG

GGATTAAAGGTGTGTGCCACCATGCCTGGTTTAACTGTCTGTTCTTTACTTCAGCTAAAAAAGAGATGATTTCTGAGAGA

TGATAAACTGATAAAAACCAGGACAGCACGACAATCA

>Sequence_617

GATCCCGGCGTTCGCTTTCATGTTCTGGGGCTTTCCCCCAATGCGGCGCGGCT

CTCGGTGCGTTTTTACTGGGAAGACGATTTTGGCCAGCTGACCGCCAACTACCAAAAATATTTGAAGGAGATGGCGATAG

AGCCTGCGCCGGACGGCTGGCCGCCACTTTGGCACTATCTCAACGATCTGGCCGTTCTGGGCAAGCGCGACAACGTGCCG

CCAAATCTGGCTGGCGACTGGATGCGCTC

>Sequence_618

GATCCCCTCTTCTCCAATGAGACTAGCCTGTGTTACATGGGCGAAAGA

ACTAGCCACGTCCTGGTTAATTAGTGTTCACACGATTCCTCAAGTGTGTAGAATGGCTCAGCACACTTTAACTTCCTTCC

TCAGGCATTTAATGTGGGTAAATGGTGTGTTTCCAGGAACCTCACAGGCAG

>Sequence_619

GATCCCAGACAAAGGTGGAAGTAAGGCCACACTGCCACCGACTCAGAG

AACAGTAGGGGAGCCCATGCCTGGTAAACAGTCCCAGACTCTTCCACCTTGTGTTGGGGTGGGTTCACTCGATGTACTCT

GAACACTCCCCTCCTATTAGAATCTATGCCGGGGCCTAGCAAACACATAAGTGGATGCTCACAATCAGCTATTGGA

>Sequence_620

GATCGCCCCGCTGCCGGCGGCTTCCAGCGCCTGCACACGTGCTTGCAGCGCGT

TGGCGTTGGTCAGCGCCTTACGGGCGACAATGACAGCGGCGATGGCAATCAGCAGCGCGAGAAATTCCATAGGTCCTC

>Sequence_622

GATCACGGCCTCGGCCGGGCGTTCAGGCCGAATTGCGGCCTAAACCTC

AGACCCTGCCAAATCAGCGGGCGCTCTGCGTCTCGCCCGCACGCCACAGGCGATAGCGAACCTCGACGTCCTGCGGCGCA

TAGACCACCAGCGGCAGGCGGCTGTTGTAACGGACAAGCGGCTGTTCCGGCGCGGCGACAAACACCTCGCGCTCGCTGTT

GGCCGGGCATCCCATCAGCGTCCCCGCCATCGCGCCCAGCGCGGGCAGGACATAATAGTCATAGCCCCAGCCTTCCGCCG

TGCGCGTCTC

>Sequence_624

TTCTCAACCGGCGTTCGAGCAGGAAATGCGTA

ACACCCCGGAAGTGCTGGACTGCTGGCTGGTCAGCGGCAACTTCGATTTTCTGGTGCGTATTGGCTGCCGCAGCATGGAA

AACTACCGGCTGCTGGCCGATACCTGGCTGACCAGCAAGAAGTTCCGGGTGGATAAAATCGTCACCCTGACCGAACTACA

GTC

>Sequence_625

GATCACCAAGCTACCACTCCATGCCCAGAGGCCTTTTATTATAGGCTGTTACA

TACCTGCCAGGTGTCACCAGGAGGAAGGGCCGCAGTGACCGTGACCTATTTAAGA

>Sequence_626

GATCGTGGCCTTCAATCGTGGCAAGTCGGGTTCTGATCGGGTGCGCAC

CTACTTGCGCCTCCTCAAGGCGCAGCCCCATGCGGCGGAGGGTGTGTTACGGGCTTGCTGGCGCATGGCCTGGGCGGACG

GCAAGGCGGATGACGCCGAGCGCGACCT

>Sequence_627

GGGAATTCAAGGCTCATAACTAAACATAATAAAAAACAATCTACAG

CAAACCAGTAGCCAACATCAAACTAAATGGAGAGAAACTTGAAGCAATCCCACTAAAATCAGAGACTAGACAACGCTGCC

CAATTTCTCCCTAACTATTAAATATATTACTTAAACTCTTAGCCAGAGAAATTAGACAACAAAAAGA

>Sequence_628

GATCCAGGTGACAAGGGAGCGGTGACAAGGGAACTGTGCTACAAAGAGA

GAGCCAGCAGGATAAGGAATCAAATAAGAACTACACTACCAGCAGAACTACACTAGTCCTTTCACAAATAGTGTCTTACG

CATATTATAAGAAGCCTATGAAATGGGAGTCACTAAATTAATTAATTAATTAATTAATTAAAGGCCCTCAAGTCTTTTAC

TGATGTTACTGGTTTTTAATGGTTCCTGAAGCCTAGCCTCTAATGCATTGCTATTCACAGCTATTTGAAACATGACAAAG

TGAGACTGATTGGGTGTTTGTAAGTCATGGATTGCAGGGCATGTCATCAAAACAGATTTTAAACCCCGGCTTCTTCTTTC

TCTTTTACATTTCACATGAAAATGACGTTTGTTCTGTGGTGAGCAGAGGCAGCCCAGACTGAATGTGTACCGTTTGACAT

GGGCACAGCCATGTCAAGGACTATTGCCTCAGAGTCATGGGAAATTGTCAAATCCCACCATCCCACCCCCATCAGGCTCT

GAGGAGTGACAAGCCCTCTTCTGGTCCTTGTGCCAATATTGGAGGTATGTACAAAAATTAGAAACCTCAGCTTCCTCCCA

TATGTTTACAGCCTGAGACTGCTCACCTACAAAGACCACGCCTCTGGGACTGGCTAACTTCTCCTCCACTCTGTGCTGTA

CACAGTTGGTGCATCTCCATCAGAGAGTGCNCACCCTCN

>Sequence_629

GATCCCCGGCAAGGTGGTCTTCACCATCGACATCCGCACGCCGCACGAGAATG

TGCTGAACGAGATGCGCGCGCGCATCGAGGACGGCATCGCTACCATCGCCGAGGCGCTGGATATCGGCTTCGAGGTCGAG

GCCGTCGGCCACTTCGACCCTGTCACCTTCGATGAAGGCTGCGTAAAAGCCATTCGCGATGCCGCCGAACGGCTCGGCTA

TTCCCATCGCGACATCGTCTCCGGTGCCGGCCACGACGCCTGC
